# Multimodal microglial and kynurenine pathway alterations across the affective-psychosis spectrum: a systematic review of patterns, heterogeneity, and dimensional implications

**DOI:** 10.1038/s41380-026-03614-3

**Published:** 2026-04-21

**Authors:** Madeleine Nussbaumer, Paul C. Guest, Kolja Schiltz, Leon Dudeck, Leila Shokati Asl, Gabriela Meyer-Lotz, Henrik Dobrowolny, Stefan Leucht, Hans-Gert Bernstein, Thomas Nickl-Jockschat, Brisa S. Fernandes, Johann Steiner

**Affiliations:** 1 Department of Psychiatry and Psychotherapy, Otto-von-Guericke-University Magdeburg, Magdeburg, Germany; 2 Laboratory of Translational Psychiatry, Otto-von-Guericke-University Magdeburg, Magdeburg, Germany; 3 Laboratory of Neuroproteomics, Department of Biochemistry and Tissue Biology, Institute of Biology, University of Campinas (UNICAMP), Campinas, Brazil; 4 Department of Psychiatry and Psychotherapy, Ludwig-Maximilian-University, Munich, Germany; 5 Center for Behavioral Brain Sciences (CBBS), Magdeburg, Germany; 6 Department of Psychiatry and Psychotherapy, Klinikum Rechts der Isar, Technical University of Munich, TUM School of Medicine and Health, Munich, Germany; 7 German Center for Mental Health (DZPG), Partner Site Munich-Augsburg, Munich, Germany; 8 Departments of Psychiatry, Neuroscience, and Pharmacology, Iowa Neuroscience Institute, University of Iowa, Iowa City, Iowa USA; 9 German Center for Mental Health (DZPG), Partner Site Halle-Jena-Magdeburg, Magdeburg, Germany

**Keywords:** Schizophrenia, Depression, Bipolar disorder

## Abstract

Immune dysregulation is implicated in patient subgroups in major depressive disorder (MDD), bipolar disorder (BD) and schizophrenia (SCZ), but the role of microglia across affective and psychotic illnesses remains unclear. We propose an integrative framework linking systemic immune drivers to microglia-related circuit engagement, cellular phenotype, and kynurenine pathway (KP) branch balance. We systematically reviewed human TSPO-PET, cerebrospinal fluid (CSF) KP metabolite, and postmortem microglial and KP studies. Quantitative synthesis focused on MDD versus SCZ. BD was integrated descriptively due to limited data. MDD showed the most reproducible in vivo signal, with increased TSPO binding in frontolimbic regions (cingulate cortex, hippocampus, prefrontal cortex), and had lower heterogeneity and higher precision than SCZ. Postmortem MDD findings were largely null for diagnosis-level increases in microglial density or classical activation markers, but suggested subtle homeostatic shifts. KP findings were localized and regionally dissociated, including cingulate QUIN-related microglial signals and reduced hippocampal QUIN immunoreactivity in single cohorts. BD evidence was sparse as TSPO-PET comprised a single study reporting hippocampal increases, and postmortem microglial markers were mostly unchanged at the diagnosis level, with suicide or psychosis stratification revealing subgroup effects. Also, BD KP data suggested anterior cingulate upstream activation with a psychosis-linked downstream signal (reduced prefrontal KMO) in a subgroup. In SCZ, TSPO-PET findings were heterogeneous with small decreases or no change, and postmortem studies indicated either activation-marker increases or loss of homeostatic microglial signatures. In addition, the most consistent biochemical signal in SCZ was a shift toward the KYNA branch (increased CSF KYNA and cortical KYNA), consistent with KP-linked glutamatergic dysregulation. Overall, microglia-related alterations appear better explained by biological subgroups and symptom dimensions than by categorical diagnoses, motivating future transdiagnostic studies with dimensional phenotyping, subgroup stratification, longitudinal designs, and microglia-specific biomarkers. Limitations include the limited cellular specificity of TSPO-PET, small sample sizes, and postmortem studies focusing on few cortical/limbic regions rather than whole-brain coverage.

## Introduction

### Rationale for comparative review across the affective–psychosis spectrum (MDD, BD and SCZ)

Major depressive disorder (MDD), bipolar disorder (BD), schizoaffective disorder, and schizophrenia (SCZ) are conventionally defined categorical diagnoses, yet they share overlapping symptom domains and can be conceptualized along dimensions of affective psychopathology and psychosis [1, 2]. MDD is characterized primarily by persistent low mood and anhedonia, often precipitated by chronic stress or trauma [3, 4]. BD is defined by recurrent episodes of depression and mania/hypomania, frequently with mixed affective features, and psychotic symptoms in a substantial subgroup [3, 5, 6]. SCZ is characterized by hallucinations, delusions, disorganized thinking and cognitive impairments, with etiological contributions from neurodevelopmental and genetic risk factors [3, 7]. Across this affective–psychosis spectrum, immune-inflammatory alterations have been described in biologically distinct subgroups rather than uniformly at the diagnostic level [8–12], raising the possibility that microglial dysfunction may contribute to core symptom dimensions (e.g., depressive severity, psychosis burden, cognitive impairment) that cut across diagnostic boundaries [13–17].

While most reviews focus on a single disorder or methodology, this review integrates evidence across modalities that index complementary levels of microglia-related biology: translocator protein positron emission tomography (TSPO-PET; brain regional-level neuroimmune engagement in vivo), cerebrospinal fluid (CSF) profiling (soluble mediators and kynurenine (KYN) pathway balance in vivo), and postmortem studies (cellular phenotypes and regional enzyme/metabolite changes). Given that the extant human literature is predominantly organized around DSM/ICD diagnostic categories, our quantitative synthesis focuses on MDD and SCZ, with a supplementary PRISMA-guided qualitative synthesis of BD (due to the low number and heterogeneity of available BD studies). At the same time, we interpret diagnostic findings within an affective-psychosis spectrum-based and dimensional framework, emphasizing symptom- and subgroup-related moderators (e.g., suicidality, psychotic features, inflammatory subtypes) that are likely to contribute to inconsistency when heterogeneous clinical presentations are pooled under a single diagnostic label.

### Moving from a neuron-centric view to a glial perspective in the affective–psychosis spectrum

**Fig. 1:**
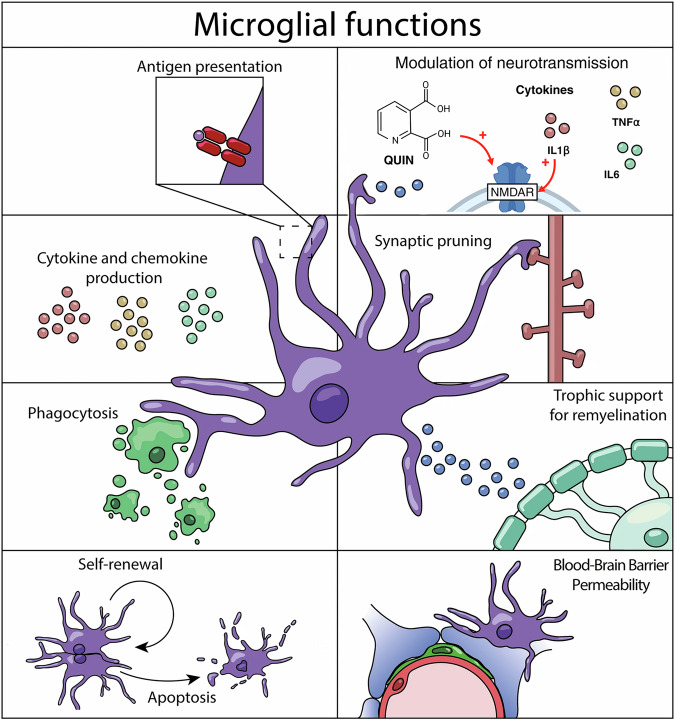
Microglial functions (adapted from Amor et al. [20]). Microglia perform diverse functions that are essential for immune defense, tissue homeostasis, and modulating neuronal circuits. The left column (top to bottom) illustrates: 1.) **Antigen presentation** via MHC class II and co-stimulatory molecules enables T-cell activation during neuroinflammation; 2.) **Cytokine and chemokine secretion** shapes immune responses and neuronal activity; 3.) **Phagocytosis** clears apoptotic cells, debris, and pathogens (bacteria, viruses, fungi, parasites); 4.) **Self-renewal and apoptosis** maintains microglial homeostasis. The right column (top to bottom) shows: 5.) **Modulation of neurotransmission** through proinflammatory cytokines (e.g., IL-1β, IL-6, TNF-α) and KYN pathway metabolites, such as quinolinic acid (QUIN), an N-methyl-D-aspartate (NMDA) receptor agonist; 6.) **Synaptic pruning** eliminates excess or weak synapses in order to refine neural circuits during development and plasticity; 7.) **Trophic support for remyelination** (after injury), **myelin maintenance, and primary myelination** (during early development of the central nervous system); 8.) **Regulation of blood–brain barrier (BBB) permeability** via interactions with endothelial cells and inflammatory mediators helps maintain or disrupt BBB integrity depending on physiological or pathological conditions.

Traditional models of MDD, BD and SCZ emphasized neuronal dysfunction, but growing evidence highlights the importance of glial cells [18, 19]. Microglia, the resident immune cells of the central nervous system (CNS), regulate synaptic plasticity, neurotransmission, neuroinflammation, and blood-brain barrier (BBB) integrity (Fig. 1) [20].

Across mood and psychotic disorders, neuroinflammatory alterations have been reported, including elevations of proinflammatory cytokines and immune signaling dysregulation in subsets of patients [9–11]. Longitudinal and high-risk studies suggest that immune activation can contribute to disease manifestation and progression [21–24]. Whether microglia are causal, compensatory, or reactive to broader immune and circuit disturbances remains unknown. Resolving this will require longitudinal and interventional human studies spanning affective and psychotic symptom dimensions.

### Microglial dysfunction, immune heterogeneity, and symptom dimensions

Postmortem studies revealed heterogeneity in microglial alterations across MDD, BD and SCZ. Some reported increased activation, but others did not [19, 25–27]. Importantly, immune and microglia-related findings are unlikely to map one-to-one onto categorical diagnoses. For example, high-inflammation subgroups have been described in SCZ and BD, characterized by elevated cytokines and immune gene expression [28–30], and inflammatory phenotypes were also reported in subsets of MDD [24, 27, 31]. Microglia respond to such disruption, potentially amplifying neuroinflammation and promoting immune cell infiltration.

Dimensional illness features – including severity and persistence of psychosis, affective symptom burden, cognitive impairment, and suicidality – may therefore moderate microglial readouts within and across diagnoses, potentially explaining some of the variability and inconsistency in the literature when samples differ in symptom severity, stage, or inflammatory subtype.

### Microglia at the interface of systemic and CNS immune dysregulation

Systemic immune challenges, such as chronic stress, infections, and environmental stressors, can compromise the BBB, activating microglia and allowing peripheral immune cells and inflammatory mediators to enter the CNS [32, 33]. In MDD, chronic stress is associated with activation of the hypothalamic-pituitary-adrenal (HPA) axis, which may in turn increase BBB vulnerability and thereby facilitate neuroinflammatory processes [33]. Prenatal infections, such as maternal immune activation (MIA), have been linked to an increased risk of SCZ in the offspring [34], potentially via abnormal synaptic pruning and disrupted neuroimmune signaling [35, 36]. Once activated, microglia alter their morphology and release proinflammatory molecules, affecting neural circuits regulating emotion and behavior  relevant to the pathophysiology of both affective disorders and SCZ [37].

### Focusing on human-based evidence

Although animal models provide valuable mechanistic insights under controlled conditions, species differences limit their direct applicability to human pathology [38]. For example, social stress alters microglial gene expression in primate dorsolateral prefrontal cortex (DLPFC) [39], and MIA models replicate schizophrenia-like dopamine dysregulation [34, 40]. While animal studies offer important translational clues, they represent a vast field beyond the scope of this review. We therefore focused on human-based evidence from cerebrospinal fluid (CSF) profiling, positron emission tomography (PET), and postmortem studies.

### Integrative conceptual framework and aims of the review

Although our synthesis summarizes findings within conventionally-defined diagnostic categories (MDD, BD and SCZ), these span overlapping dimensions of affective psychopathology and psychosis. Accordingly, we use an integrative, spectrum-based conceptual framework (Table 1) to organize how immune dysregulation may relate to microglia-related neuroimmune mechanisms and downstream brain dysfunction, and how these processes may link to symptom dimensions across disorders.

**Table 1:** Spectrum-based integrative conceptual framework (a priori; evidence-informed) linking immune dysregulation to microglia-related brain dysfunction across MDD, BD and SCZ.

Level	MDD	BD	SCZ
**1. Predominant systemic/clinical context (immune-relevant drivers/subgroups)**	• Chronic stress/HPA-axis dysregulation; immune activation in a subgroup [33, 207]. • Low-grade systemic inflammation on average, with substantial between-person heterogeneity [208].	• Mood episodes and sleep/circadian disruption are linked to immune activation; inflammatory markers show state and trait components [156, 209]. • Markers of inflammation and stress identify cross-diagnostic subgroups spanning BD and SCZ [28].	• Neurodevelopmental immune risk (e.g., maternal immune activation/infection) and complement/synaptic pruning hypotheses [210, 211]. • Inflammatory biotypes/subgroups documented across cohorts [28, 212].
**2. Microglia-related mechanisms (conceptual; heterogeneity & marker considerations)**	• Stress and peripheral inflammation can shape microglia-related signaling and neuroplasticity; effects likely state/subtype dependent [207, 213]. • Interpretation depends on cellular specificity of readouts (e.g., TSPO vs microglia markers) [159, 214].	• Microglia-related mechanisms are expected to be phase- and biotype-dependent, given state/trait inflammatory variability [28, 156]. • Psychotic features occur in many patients and motivate subgrouping by the psychosis dimension in microglia/KP studies [5, 28].	• Microglia implicated in synaptic pruning/circuit maturation and immune–brain interactions [210, 215]. • Microglia readouts are marker-dependent; TSPO-PET has limited cellular specificity and is influenced by TSPO polymorphisms [159].
**3. KYN pathway (KP) immune–neurotransmission interface (conceptual)**	• Inflammation can increase TRP → KP flux (IDO/TDO), influencing glutamatergic signaling and neuroplasticity [76, 207]. • QUIN is an NMDA agonist mainly generated by activated microglia/macrophages, whereas KYNA is an NMDA antagonist largely produced by astrocytes [73, 78].	• KP activation has been linked to mood disorders in systematic reviews/meta-analyses [216]. • KP may mediate immune-to-glutamate/NMDAR effects with potential cross-diagnostic relevance for psychosis and cognition [76, 217].	• Mechanistic models propose that KYNA-mediated NMDAR modulation may contribute to psychosis and cognitive symptoms [76, 217]. • Inflammation–KP coupling has become a major focus of immunopsychiatry biomarker and therapeutic research [159].
**4. Symptom dimensions emphasized in a spectrum view (cross-diagnostic)**	• Affective psychopathology varies dimensionally; cognitive impairment and suicidality contribute to heterogeneity [3].	• Affective psychopathology spans depression and mania/hypomania; psychotic symptoms occur in a substantial subset [3].	• Psychosis and negative symptom dimensions with prominent cognitive impairment; affective symptoms contribute to cross-diagnostic overlap [3, 218].

This table provides an evidence-informed, a priori framework and does not summarize the results of the present systematic review (synthesized separately in the Results and Discussion). TSPO-PET primarily supports macro-scale regional mapping of neuroimmune engagement (limited spatial resolution and cell-type specificity), enabling inference at the level of major regions/networks rather than microcircuits. CSF provides an in vivo window into soluble mediators and KP balance but does not localize brain sources. Postmortem studies provide cellular/phenotypic and region-specific resolution but reflect end-stage tissue and are sensitive to peri-mortem confounds. Neurotransmission mechanisms are only partly captured here, primarily via the KP–glutamate/NMDA interface. *BD* bipolar disorder, *CSF* cerebrospinal fluid, *HPA* hypothalamic-pituitary-adrenal, *IDO* indoleamine 2,3-dioxygenase, *KP* kynurenine pathway, *KYNA* kynurenic acid, *MDD* major depressive disorder, *NMDA* N-methyl-D-aspartate, *NMDAR* N-methyl-D-aspartate receptor, *PET* positron emission tomography, *QUIN* quinolinic acid, *SCZ* schizophrenia, *TDO* tryptophan 2,3-dioxygenase, *TSPO* translocator protein.

Within this framework, TSPO-PET is an in vivo macro-scale, brain regional index of neuroimmune engagement (limited spatial resolution; not circuit- and microglia-specific). CSF studies provide an in vivo window into soluble mediators and KP metabolite balance, while postmortem studies enable higher-resolution characterization of microglial markers and region-/compartment-specific KP enzyme and metabolite patterns. Because these modalities interrogate only a subset of relevant mechanisms, the framework should be viewed as a heuristic scaffold rather than a complete pathophysiological model. This is particularly true for neurotransmission, where available human CSF and postmortem data most directly inform the KP–glutamate/NMDA interface, whereas other neurotransmitter systems are not comprehensively captured by the modalities summarized here. The section entitled Comparative analysis of microglial involvement in MDD, BD and SCZ, Table 2 and Supplementary Figure 3 synthesize modality-specific results that populate each level of this framework (with BD integrated descriptively where evidence is limited), and the final section of this review returns to these integrated patterns to inform clinical translation and future research directions.

**Table 2:** Summary of all microglia-related brain and CSF findings in MDD, BD and SCZ presented in the comparative analysis.

Feature	MDD Findings	BD Findings	SCZ Findings	Cross-Diagnostic Comparison	Strength of Findings	Brain Regional Focus	Heterogeneity & precision (I², τ², v, rCIW)
**MICROGLIA IN TSPO-PET IMAGING STUDIES**							
**TSPO binding**	↑ Hippocampus (SMD = 0.69, 95% CI: 0.36–1.02; p < 0.001) [8] ↑ Cingulate cortex (SMD = 0.82, 95% CI: 0.45–1.19; p < 0.001) [8] ↑ PFC (SMD = 0.42, 95% CI: 0.11–0.72; p = 0.007) [8] ↑ Thalamus (n.s.; SMD = 0.57; p = 0.127) [8] ↑ Whole-brain cortical GM (n.s.; SMD = 0.53; p = 0.062) [8]	Single study [98]: ↑ right hippocampus [(11)C]-(R)-PK11195 binding potential (p = 0.033); trend ↑ left hippocampus. Evidence base too limited for meta-analysis or quantitative cross-diagnostic comparison (k = 1).	Overall ↓/↔ TSPO across ROIs [8]. Only significant pooled finding: ↓ whole-brain cortical GM (VT-based: SMD = −0.51, 95% CI: −0.92 to −0.10; p = 0.016) [8].	MDD > SCZ in cingulate (Z = 3.96; p < 0.001), hippocampus (Z = 3.06; p = 0.002), and thalamus (Z = 2.02; p = 0.044); trend for cortical GM (Z = 1.92; p = 0.055) [8]. BD: no quantitative comparison possible (single PET study) [98].	MDD: Moderate-Strong (Criteria 1 + 2) - replicated, moderate-to-large SMDs in frontolimbic ROIs [8]. BD: Insufficient evidence (k = 1) [98]. SCZ: Weak-Moderate (Criteria 1 + 2) - mostly non-significant decreases with substantial heterogeneity in several ROIs [8].	MDD: Hippocampus, cingulate cortex, PFC (frontolimbic circuits). BD: Hippocampus (single study). SCZ: Whole-brain cortical GM (VT-based ↓) and otherwise mixed/heterogeneous across regions.	MDD: Hippocampus/cingulate/PFC show low heterogeneity and narrow–moderate rCIW (I² = 0–37.6%; rCIW=0.90–1.47) [8]. Whole-brain cortical GM and thalamus show higher dispersion (I² = 72.2–75.8%; rCIW=2.10–2.57) [8]. BD: n.a. (k = 1) [98]. SCZ: Mostly moderate–high I² with wide rCIWs and higher v (lower precision) in several ROIs (e.g., VT thalamus I² = 83.0%, τ²=0.761; PFC rCIW=37.25) [8].
**MICROGLIA IN POSTMORTEM STUDIES**							
**Microglial density & marker expression** **(homeostatic vs. activated)**	↔ 7 of 9 studies: no diagnosis-level increase in density or activation markers [99–105]. Region-/phenotype-specific signals: ↑ amoeboid IBA1+ microglia in VLPFC [106]; ↑ primed/resting ratio in ACC white matter (no overall density change) [107]. Non-inflammatory/homeostatic signature: ↑ TMEM119, P2Y12, CX3CR1, CCR5 with no ↑ in classical inflammatory markers [104, 105]. Suicide-stratified: ↑ HLA-DR+ microglia in suicidal cases (DLPFC, ACC, thalamus) [100]; ↓ HLA-DR+ microglia in non-suicidal cases (dorsal raphe) [99].	↔ Predominant pattern (14 studies): no diagnosis-level increases across DLPFC, cingulate/ACC, thalamus, or hippocampus [100, 101, 111–116]. Exception: Rao et al. reported ↑ HLA-DR+ microglia (qualitative) and ↑ CD11b in frontal cortex [110]. Some studies reported ↓ activation-associated markers rather than increases (e.g., ↓ CD11b/CD68 [113]; ↓ CD68 and ↓ TREM2 mRNA in DLPFC [112]). Suicide-stratified: ↑ P2RY12+ microglia in hippocampus only in suicidal BD [116]; non-suicidal subgroup reductions reported in aMCC IBA1 [118] and DLPFC P2RY12 [112].	Meta-analytic divergence in density [25, 26]. Van Kesteren et al. [25]: ↑ microglial density (SMD = 0.69, 95% CI: 0.24–1.14; p = 0.003), especially temporal cortex (p = 0.033), with high heterogeneity (I² = 68.6%). Snijders et al. [26]: ↔ overall density (g = 0.14, 95% CI: −0.10 to 0.39; p = 0.250, I² = 18.9%); temporal cortex increase attenuated after outlier removal. Gene expression - Van Kesteren et al. [25]: ↑ pro-inflammatory cytokine expression (SMD = 0.37, 95% CI: 0.11–0.62; p = 0.005). Gene expression - Snijders et al. [26]: ↓ mature/ homeostatic microglial markers without classical immune activation (e.g., TMEM119 g = −0.42; p < 0.001). Symptom link: ↑ hippocampal HLA-DR+ associated with positive symptoms [117].	Across mood disorders (MDD/BD), postmortem microglial findings are predominantly negative at the diagnosis level, with region- and subgroup-specific effects (including suicide-related differences). SCZ shows more pronounced but heterogeneous alterations: either ↑ density/pro-inflammatory expression in some meta-analytic syntheses [25] or loss of homeostatic microglial signature without classical activation [26].	MDD: Weak-Moderate (Criterion 1) - mostly null findings; replicated evidence for a non-inflammatory/homeostatic signature [104, 105]. BD: Weak-Moderate (Criterion 1) - predominantly null; one positive study and several reports of decreases [110, 112, 113]. SCZ: Moderate (Criteria 1-2) - divergent meta-analyses but consistent evidence for altered microglial marker profiles [25, 26].	MDD/BD: No consistent significant regional changes. SCZ: Temporal cortex contributes most consistently; plus PFC/cingulate and hippocampus in symptom-linked studies.	MDD/BD: no pooled I²/τ²/v/rCIW available. SCZ: Density meta-analyses range from low to moderate heterogeneity (I² = 18.9–68.6%) with moderate rCIW (1.30–2.0) [25, 26]. Microglial gene-expression meta-analysis shows low I² = 26.7% with narrow rCIW=0.31 [26].
**MICROGLIA-RELATED KP: CSF STUDIES**							
**KP metabolites in CSF**	Inam et al. [134] (11 studies; 217 MDD, 254 controls): ↔ KYNA, KYN, TRP; Trend ↑ QUIN (SMD = 2.66, CI: −1.04 to 6.35; rCIW=2.78; p = 0.159; I² = 98%; k = 2; n = 136); 3-HK, 3-HAA, PIC not systematically analyzed.	Inam et al. [134] (6 studies; 246 BD, 340 controls): ↔ TRP; Trend ↑ KYNA (SMD = 1.40, CI: −0.28 to 3.09; rCIW=2.41; p = 0.103; I² = 98%; k = 2; n = 377); KYN and QUIN not systematically meta-analyzed in BD.	Inam et al. [134] (8 studies; 238 SCZ, 240 controls): ↑ KYNA (SMD = 2.64, 95% CI: 1.16–4.13; rCIW=1.13; p < 0.001; I² = 96%; k = 6). Rømer et al. [11]: ↑ KYNA (SMD = 1.58, 95% CI: 0.34–2.81; p = 0.013; I² = 96%); ↑ KYN (SMD = 1.00, 95% CI: 0.64–1.36; p < 0.001; I² = 17%).	SCZ: robust ↑ KYNA (and ↑ KYN in secondary meta-analysis) [11, 134]. MDD: no consistent CSF KP changes; QUIN signal inconclusive (k = 2) [134]. BD: trend ↑ KYNA based on k = 2 with extreme heterogeneity; insufficient KYN/QUIN synthesis [134]. Consistent with MRS patterns: ↓ glutamate/glutamine in MDD vs ↑ in SCZ [147, 148].	MDD: Weak (Criterion 1) - largely null; QUIN trend based on k = 2 [134]. BD: Weak (Criterion 1) - KYNA trend based on k = 2 with I² = 98% [134]. SCZ: Strong (Criteria 1 + 2) - replicated ↑ KYNA and ↑ KYN across meta-analyses [11, 134].	No (CSFKYN metabolite levels do not provide direct information on brain regional alterations.)	MDD: QUIN trend shows extreme heterogeneity (I² = 98%, rCIW=2.78; wide) [134]. BD: KYNA trend shows extreme heterogeneity (I² = 98%, rCIW=2.41; wide) [134]. SCZ: KYNA increases show extreme heterogeneity (I² = 96%, rCIW=1.13–1.56; moderate) [11, 134], whereas KYN shows low heterogeneity and narrow CI (I² = 17%, rCIW=0.72) [11].
**MICROGLIA-RELATED KP: POSTMORTEM BRAIN STUDIES**							
**KP metabolites & enzymes in brain**	6 postmortem studies. QUIN (IHC): ↑ QUIN+ microglia in sACC and aMCC (MDD subgroup) and ↔ in pACC [74]; ↓ QUIN+ microglia in hippocampal CA1 [135]. Upstream regulation: ↑ TDO2+ astrocytes in ACC white matter [136]; ACC metabolites largely ↔ (TRP, 3-HAA, NAM) [137]. VLPFC (depressive disorder NOS): ↓ QUIN, ↓ KYN/TRP, ↓ IDO1/2, ↓ TDO2 [106]. ACC (BA24): sex-specific ↓ KYNA in females and ↑ KAT II [138].	5 postmortem studies. QUIN (IHC): ↔ QUIN+ microglia in sACC/aMCC/pACC (BD subgroup) [74]; ↓ QUIN+ microglia in hippocampal CA1 [135]. ACC: ↑ KYN and ↑ TDO2+ astrocytes; ACC metabolites largely ↔ (TRP, 3-HAA, NAM) [136, 137]. PFC (psychotic BD): ↓ KMO expression [139].	10 postmortem studies. Cortical shift toward KYNA accumulation: ↑ KYNA in multiple cortical regions [80, 140–142] (with some ↔ findings [136, 143]); often with ↑ KYN and/or ↑ KYN/TRP ratio [80, 136, 140, 142]. QUIN is region-dependent: ↓ QUIN immunoreactivity in hippocampal CA1 [144] vs ↑ QUIN in DLPFC white matter [140]. Enzymes: ↑ upstream TDO2/TDO in anterior PFC WM [145], ACC [136], and DLPFC [141, 142]. Downstream constraint in some cohorts: ↓ KMO and/or ↓ 3-HAO activity with inflammation-state modulation [141, 142, 146].	Shared across mood disorders: ↓ hippocampal CA1 QUIN immunoreactivity in both MDD and BD [135]. Divergence within mood disorders: ↑ cingulate QUIN+ microglia in MDD but not BD [74]. Psychosis-related features: BD (psychotic subtype) shows ↓ KMO in PFC [139], paralleling SCZ patterns of KYNA/KMO pathway rebalancing [80, 140–142, 146]. SCZ-distinct: pervasive cortical KYNA accumulation with upstream enzyme upregulation and downstream constraints [80, 136, 140, 142, 145, 146].	MDD: Weak-Moderate (Criterion 1) - few studies, region-dependent effects; key QUIN findings based on single studies [74, 135]. BD: Weak-Moderate (Criterion 1) - limited number of studies; phenotype-specific (psychotic BD) downstream finding [139]. SCZ: Moderate (Criterion 1) - KYNA accumulation replicated across multiple cohorts, but not uniform (some ↔ findings) [80, 136, 140–143].	MDD: No consistent multi-region diagnosis-level signature; most informative signals are a cingulate–hippocampal dissociation ( ↑ QUIN+ microglia in sACC/aMCC vs ↓ QUIN+ microglia in hippocampal CA1; each based on single cohorts). BD: Evidence sparse and heterogeneous; signals cluster in ACC (upstream activation: KYN/TDO2 ↑) and hippocampal CA1 (QUIN+ microglia ↓), with a PFC KMO ↓ signal confined to psychotic BD (single study). SCZ: Most consistent pattern is prefrontal cortical involvement (DLPFC/anterior PFC; often including white matter) with a shift toward the KYNA branch (KYNA ↑ with KYN ↑ and/or upstream TDO/TDO2 ↑.	No pooled I²/τ²/v/rCIW available.

**a) Criteria for “strength of findings” (note which criterion was applied: 1 = consistency; 2 = effect size):**

1. Consistency Across Studies

• Strong: Replicated across multiple independent studies.

• Moderate: Some replication; findings inconsistent or limited in scope.

• Weak: Inconsistent findings or based on few/small studies.

2. Effect Size

• Strong (large magnitude): ∣effect size∣ > 0.7.

• Moderate (medium magnitude): ∣effect size∣ 0.3–0.69.

• Weak (small magnitude): ∣effect size∣ < 0.3.

Notes: We use ∣SMD∣ to classify magnitude; direction (↑/↓) is shown elsewhere in the table. This mapping aligns the table’s Strong/Moderate/Weak labels with the Supplement’s Large/Moderate/Small categories to avoid confusion.

**b) Between-study heterogeneity**

I²: Between-study heterogeneity (in %); interpreted as low (<40%), moderate (40–75%), or high (>75%). Not applicable when k = 1; interpret cautiously when k ≤ 3.

τ²: approximated absolute between-study variance of true effects (see Supplementary Methods).

**c) Precision of meta-analytic estimates**

v (SMD²): approximated typical within-study sampling variance (see Supplementary Methods); higher v = lower precision.

rCIW: Relative width of the 95% confidence interval (CI); calculated as (CI_upper_ – CI_lower_)/|SMD|. Interpretation: narrow (≤ 1.0), moderate (1.0–2.0), wide (>2.0).

**d) Cross-diagnostic comparison of effect sizes**

Pooled SMDs for MDD vs SCZ were compared with two-sample Z-tests for independent estimates. Positive Z indicates MDD > SCZ. BD was not included in Z-tests due to insufficient or non-comparable pooled estimates (e.g., TSPO-PET: k = 1); BD comparisons are therefore descriptive where possible.

- BD TSPO-PET: single study [98] precludes meta-analysis; hippocampal increase suggests possible overlap with MDD pattern but evidence remains insufficient for definitive conclusions.

- BD CSF KP: KYNA trend based on k = 2 studies with extreme heterogeneity (I² = 98%); KYN and QUIN were not systematically meta-analyzed in BD [134].

- Suicide stratification: important moderator across diagnoses; effects are region- and marker-dependent rather than a uniform ‘activated’ state.

- Methodological factors in SCZ postmortem microglial meta-analyses: Van Kesteren et al. [25] used markers like HLA-DR and CD68, which label both microglia and infiltrating macrophages, potentially overestimating microglial activation. In contrast, Snijders et al. [26] included original data and applied microglia-specific markers (e.g., TMEM119) and addressed outliers, highlighting altered microglial identity without classical activation.

*3-HAA* 3-hydroxyanthranilic acid, *3-HK* 3-hydroxykynurenine, *ROI* region of interest, *ACC* anterior cingulate cortex, *aMCC* anterior midcingulate cortex, *BA* Brodmann area, *BD* bipolar disorder, *CI* confidence interval; *CSF* cerebrospinal fluid, *DLPFC* dorsolateral prefrontal cortex, *g* Hedges’ g, *GM* gray matter, *I*², inconsistency index, *k* number of studies, *KAT* kynurenine aminotransferase, *KMO* kynurenine 3-monooxygenase, *KP* kynurenine pathway, *KYN* kynurenine, *KYNA* kynurenic acid, *MDD* major depressive disorder, *NAM* nicotinamide, *PFC* prefrontal cortex, *QUIN* quinolinic acid, *rCIW* relative confidence interval width; *RT* reference tissue, *sACC* subgenual anterior cingulate cortex, *SCZ* schizophrenia, *SMD* standardized mean difference, *TDO* tryptophan 2,3-dioxygenase, *TRP* tryptophan, *TSPO* translocator protein, *v* within-study variance, *VLPFC* ventrolateral prefrontal cortex, *VT* volume of distribution, *WM* white matter, *τ*², between-study variance; ↑, increased; ↓, decreased; ↔, no significant change.

Our review addresses the following questions:

1. Across TSPO-PET, CSF KP metabolites, and postmortem microglial/KP markers, which signals are most consistently reported in MDD and SCZ, and – where evidence permits –BD?
2. Do mood disorders (MDD/BD) and SCZ show distinct microglia-related profiles across modalities, and which candidate signals appear cross-diagnostic across the affective–psychosis spectrum (considering strength of evidence by modality and diagnosis)?
3. At the spatial scale supported by these methods (regional PET patterns and postmortem ROIs), which brain regions are most consistently implicated, and where available, how do findings relate to symptom dimensions (affective psychopathology, psychosis, cognition) rather than diagnosis alone?
4. How consistent are findings within each diagnosis, and to what extent does heterogeneity and inconsistency reflect differences in symptom severity, illness stage, medication exposure, suicidality, and inflammatory subtypes, noting that moderator evidence is often descriptive rather than formally tested?
5. What is the potential for multimodal microglia-related measures to contribute to biologically informed stratification across diagnostic boundaries, and what validation gaps remain?
6. Which microglia- or KP-targeted therapies have been tested in mood and psychotic disorders, and what do current data suggest about subgroup-specific promise and limitations?

## Methods

### Literature search and study selection

We conducted a comprehensive literature search last updated on July 5, 2025 (MDD/SCZ) and January 13, 2026 (BD), using two complementary strategies:

For this narrative overview, we performed a targeted, unsystematic search via PubMed and Google Scholar, using iterative keyword refinements and backward/forward citation chasing. Selection of sources prioritized broad scope, mechanistic relevance, and recency; this narrative stream was not intended to be exhaustive.

For the quantitative synthesis of microglial effects, we conducted a systematic search and study selection following a PRISMA-compliant workflow (Supplementary Figure 1 for MDD/SCZ; Supplementary Figure 2 for BD). We searched PubMed, Scopus, and Web of Science for human studies of microglial alterations in MDD and SCZ (see Supplementary Methods for full details). From eligible sources, we extracted standardized effect sizes (standardized mean differences [SMD] or Hedges’ g) with 95% confidence intervals (CI) for comparisons of MDD or SCZ versus controls. For meta-analyses, we also recorded heterogeneity estimates (I²) and precision metrics (relative CI width, rCIW), and we approximated τ² (between-study variance on the SMD² scale) and v (typical within-study variance) when these were not directly reported. We also conducted a systematic literature search on BD (Supplementary Methods) and integrated BD TSPO-PET, postmortem microglial, and KP findings descriptively, as study numbers were insufficient for meta-analytic testing. This review was not prospectively registered, and no separate protocol was prepared. No formal assessment of individual-study risk of bias, reporting bias, or certainty of evidence was undertaken. Methodological limitations are discussed narratively in the Methodological considerations section, and qualitative strength-of-findings ratings are summarized in the Table 2 footnotes.

### Effect size comparisons

Within each diagnostic group, we compared standardized mean differences (SMDs) across brain regions to identify patterns of region-specific microglial alterations. To test for cross-diagnostic differences in effect size magnitude, we used two-sample Z-tests on matched regional effects between MDD and SCZ (Supplementary Methods). BD was not included in Z-tests because comparable pooled estimates were unavailable.

### Assessment of reproducibility, precision, and heterogeneity

To minimize methodological confounds when comparing variability between disorders using published summary data, we employed a two-tier design: (1) analyses of volume-of-distribution (VT)–based PET studies only (a method-matched subset), and (2) overall analyses including mixed imaging methodologies. Full details are provided in the Supplementary Methods. Cross-diagnostic comparisons of heterogeneity/precision were treated as descriptive and interpreted cautiously, particularly when the number of studies was small.

### Structured vote counting for non-meta-analyzed outcomes

For outcomes lacking pooled effect size estimates, we employed a structured vote-counting approach. Individual study findings were grouped by brain region and marker type, then categorized by direction of effect (increase, decrease, or no change) to qualitatively assess the consistency of findings across studies. This approach also captured outcomes where pooling was not feasible in BD.

## Microglial functions and influence on neuronal networks

Microglia are the primary immune cells in the brain, playing a key role in homeostasis and response to pathology. Originating from embryonic yolk sac precursors [41], microglia migrate into the CNS during development and renew throughout life [42, 43]. They interact with neurons, astrocytes, oligodendrocytes, and endothelial cells, contributing to CNS immunity by detecting and responding to pathogens, cellular debris, and damaged cells [20, 44–46].

### Classical immune functions

Microglia perform multiple classical immune functions (Fig. 1):

- Antigen presentation - Expression of MHC class II molecules and other antigen-presenting markers, enabling T-cell activation during neuroinflammation [20].
- Cytokine and chemokine secretion - Release of both pro- and anti-inflammatory cytokines and chemokines, influencing immune responses, synaptic plasticity, and neuronal activity [20].
- Phagocytosis - Clearance of apoptotic cells, debris, and pathogens (bacteria, viruses, fungi, parasites), limiting inflammation and maintaining CNS integrity.

### BBB modulation

Microglia regulate BBB integrity through cross-talk with endothelial cells, astrocytes, and pericytes [47] (Fig. 1). Under physiological conditions, they release transforming growth factor (TGF)-β and may express claudin-5 to stabilize tight junctions. In pathological states, activated microglia secrete tumor necrosis factor (TNF)-α, interleukin (IL)-1β, IL-6, reactive oxygen species, and matrix metalloproteinases (MMPs), disrupting tight junctions and enabling immune cell infiltration. This bidirectional interaction can perpetuate neuroinflammation. In MDD and SCZ, BBB disruption may expose microglia to peripheral immune signals, exacerbating neuroinflammatory responses in susceptible individuals [48, 49]. Therefore, BBB breakdown may link systemic immune dysregulation and microglial activation in affective and psychotic disorders.

### Microglial influence on neuronal circuits

#### Structural modulation

Microglia shape brain architecture through synaptic pruning, extracellular matrix (ECM) remodeling, and support of oligodendrocytes and myelination (Fig. 1).

- Synaptic pruning and ECM regulation - Microglia eliminate excess synapses via complement proteins (e.g., C1q, C3) and CX3C motif chemokine ligand 1 (CX3CL1)-CX3C motif chemokine receptor 1 (CX3CR1) signaling [50–53]. They also remodel perineuronal nets (PNNs) and ECM via secretion of MMPs, A disintegrin and metalloproteinase with thrombospondin motifs (ADAMTS), cathepsins, and phagocytosis of components like aggrecan [54–57]. Excessive microglial activation may destabilize neuronal circuits by over-pruning and PNN degradation in SCZ, whereas in MDD it appears to primarily affect synaptic plasticity and signaling rather than developmental synaptic elimination [58, 59].
- Oligodendrocyte and myelin support - Microglia support oligodendrocyte precursor cell maturation and myelination via insulin-like growth factor (IGF)-1 and TGF-β activity [60, 61], and promote remyelination by clearing myelin debris, crucial for preserving brain connectivity [18, 62]. Dysregulation of these microglia-mediated functions may therefore contribute to white matter abnormalities and disrupted structural connectivity reported in SCZ and, to a lesser extent, MDD [18, 62].

#### Functional modulation of neurotransmission

Microglia influence neuronal signaling via cytokine release and modulation of KP metabolism (Fig. 1).

**Fig. 2:**
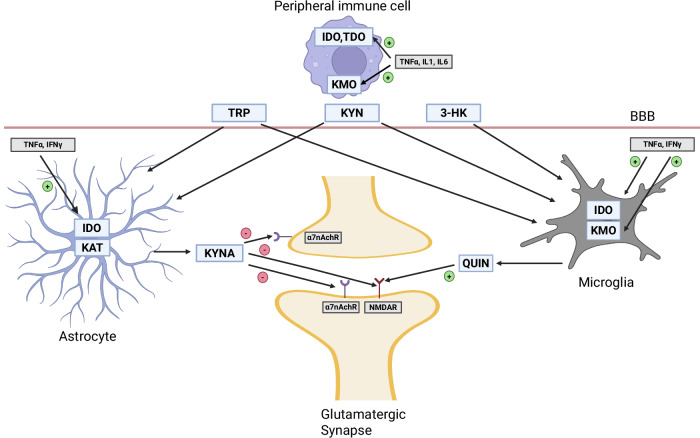
KP and quadripartite synapse (figure was conceptually inspired by Schwarcz et al. [150]). The KP metabolizes the amino acid tryptophan (TRP), which enters the brain from the blood along with its intermediate metabolites KYN and 3-hydroxykynurenine (3-HK). These substrates, as well as locally synthesized ones, are processed primarily by glial cells. In microglia, the enzyme kynurenine 3-monooxygenase (KMO) converts KYN into 3-HK, 3-hydroxyanthranilic acid (3-HAA), and ultimately quinolinic acid (QUIN), a neuroactive metabolite and N-methyl-D-aspartate (NMDA) receptor agonist [219]. In contrast, astrocytes lack KMO but express kynurenine aminotransferase (KAT), which converts KYN into kynurenic acid (KYNA), an antagonist of NMDA and α7 nicotinic acetylcholine receptors (α7nAChRs) [220, 221]. QUIN and KYNA are released into the extracellular space, where they modulate synaptic signaling at pre- and postsynaptic sites by acting on NMDA and α7nACh receptors. Inflammatory conditions enhance KP activation both centrally and peripherally: cytokines upregulate indoleamine 2,3-dioxygenase (IDO) in brain immune cells, while in peripheral tissues (especially the liver and certain immune cells) TRP is also converted to KYN by tryptophan 2,3-dioxygenase (TDO) and IDO [71, 150, 222]. This leads to an increased influx of KP metabolites formed in the periphery that cross the blood-brain barrier (BBB), in parallel with direct cytokine-mediated stimulation of the kynurenine pathway in glial cells [141]. In addition to influencing glutamatergic signaling, KP activation depletes TRP available for serotonin synthesis (not shown in this figure) [13]. *3-HAA* 3-hydroxyanthranilic acid; *3-HK* 3-hydroxykynurenine; *α7nAChR* α7 nicotinic acetylcholine receptor; *BBB* blood–brain barrier; *IDO* indoleamine 2,3-dioxygenase; *KYN* kynurenine; *KYNA* kynurenic acid; *KMO* kynurenine 3-monooxygenase; *KAT* kynurenine aminotransferase; *NMDA* N-methyl-D-aspartate; *QUIN* quinolinic acid; *TDO* tryptophan 2,3-dioxygenase; *TRP* tryptophan (Created in BioRender. Steiner, J. (2026) https://BioRender.com/6nlhh0m).
  - ◦ The microglial enzyme kynurenine monooxygenase (KMO) converts kynurenine to 3-HK and QUIN [75, 76].
  - ◦ If KMO is inhibited or absent, kynurenine is converted by kynurenine aminotransferase (KAT) to kynurenic acid (KYNA), an NMDA receptor antagonist [77].
  - ◦ Primarily produced by astrocytes [78], KYNA may reflect an underlying metabolic shift with the potential to contribute to NMDA receptor hypofunction and the emergence of psychosis [79, 80].

- Cytokine-induced neurotransmitter dysregulation
  - ◦ Glutamate: TNF-α promotes astrocytic glutamate release and IL-1β enhances N-methyl-D-aspartate (NMDA) receptor signaling via prostaglandin synthesis [63, 64]. Glutamergic dysregulation is implicated in both MDD and SCZ, with region- and symptom-specific contributions [65, 66].
  - ◦ Dopamine: IL-6 and TNF-α suppress dopamine release, possibly contributing to depressive and negative symptoms [67, 68].
  - ◦ GABA: IL-1β and IL-6 impair interneuron function, disrupting excitatory/inhibitory balance [63], which is central to cortical circuit dysfunction in SCZ and may similarly modulate stress-sensitive affective circuits in MDD [69, 70].
  - ◦ Serotonin: Inflammatory stimuli shift tryptophan (TRP) metabolism toward the KP via indoleamine 2,3-dioxygenase (IDO) in microglia, astrocytes, and neurons, and tryptophan 2,3-dioxygenase (TDO) in neurons [71, 72].

- KP-mediated modulation of glutamate and serotonin (Figs. 1, 2)
  - ◦ Microglia/macrophage-derived quinolinic acid (QUIN), an NMDA agonist, can drive glutamatergic hyperactivity [73], which could lead to affective psychopathology [74].

This concept extends the tripartite synapse model, which recognizes astrocytes as modulators of synaptic transmission [81], to a quadripartite model including microglia. Here, microglia influence synaptic function through phagocytosis, cytokine release, and extracellular signaling [82].

### Microglial phenotyping and marker expression

**Fig. 3:**
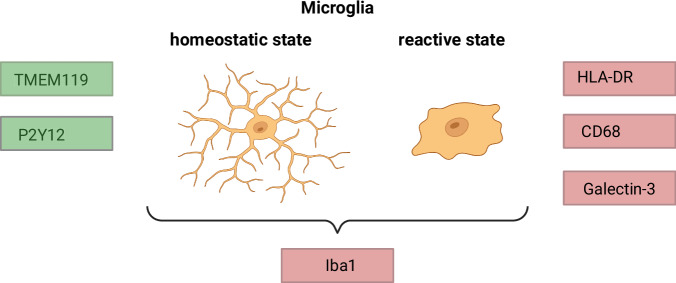
Microglial markers. This figure shows frequently used markers for microglia and their suitability for detecting resting, homeostatic, or activated microglia. Markers highlighted in green are specific to microglia in brain tissue. Markers highlighted in red are also expressed in other immune cells, such as macrophages, neutrophils, and monocytes (Created in BioRender. Steiner, J. (2026) https://BioRender.com/w6nvq0z).

Microglial phenotypes vary along a dynamic continuum shaped by the CNS microenvironment (Fig. 3). Upon activation, they shift from ramified to amoeboid morphology with altered cytokine profiles [20]. The traditional proinflammatory (IL-1, IL-6, CD86, CCR7) vs. anti-inflammatory (IL-10, TGF-β, CD163, CD206) classification [83] has been replaced by a more nuanced spectrum-based model [84, 85].

#### General markers

- Ionized calcium-binding adapter molecule 1 (Iba1) - A pan-microglial marker expressed across all microglial phenotypes, and in macrophages [20].
- Transmembrane protein 119 (TMEM119) and purinergic receptor P2Y12 (P2RY12) - Homeostatic markers^1^specific to microglia under physiological conditions, downregulated upon activation [86, 87].
  - ◦ TMEM119 may co-express with histocompatibility complex (MHC) II and CD68 during inflammation (e.g., spinal cord injury) [88], but is absent in multiple sclerosis or stroke lesions [86].
  - ◦ P2RY12 supports microglia recruitment to injury sites [89], and subsequent downregulation leads to reduced size of microglial processes [90].

#### Activation markers

- MHCII (e.g., HLA-DR) and CD68, but these are also expressed in monocytes/macrophages [91, 92].
- Galectin-3 is upregulated in reactive microglia but is also present in various immune cells, including macrophages, neutrophils, mast cells, and T cells [93].

Given marker overlap, accurate microglial phenotyping requires combining multiple markers to distinguish activation states across physiological and pathological conditions.

## Comparative analysis of microglial involvement in MDD, BD and SCZ

The specific involvement of microglia in MDD, BD and SCZ remains an area of active study. Microglial alterations may vary across brain regions, disease stages, and symptom dimensions, contributing to heterogeneity in findings.

In line with the integrative framework outlined in the Introduction (Table 1), we structured the Results to move from in vivo brain regional-level neuroimmune engagement (TSPO-PET), to tissue-resolved microglial phenotypes (postmortem marker studies), and finally to downstream immune–neurotransmitter interfaces indexed by KP metabolites and enzymes (CSF and postmortem). Where available, we highlight moderators (e.g., suicidality, psychotic features, inflammatory subtypes) that may drive within-diagnosis variability. In the final section we will integrate these modality-specific findings into the conceptual framework.

### Microglial PET studies

The 18 kDa translocator protein (TSPO) is a mitochondrial membrane protein involved in steroidogenesis, energy metabolism, and cellular stress responses. Although not specific to microglia, TSPO-PET imaging remains a key method for assessing glial activation in vivo.

To ensure a comprehensive synthesis, we compared the original studies included in prior meta-analyses [27, 94–97] with those covered by De Picker et al. [8]. This confirmed that De Picker et al. subsumed all relevant diagnostic TSPO PET studies, and we therefore used this meta-analysis as the primary data source (see Suppl. Methods).

#### TSPO-PET findings in MDD

**Fig. 4:**
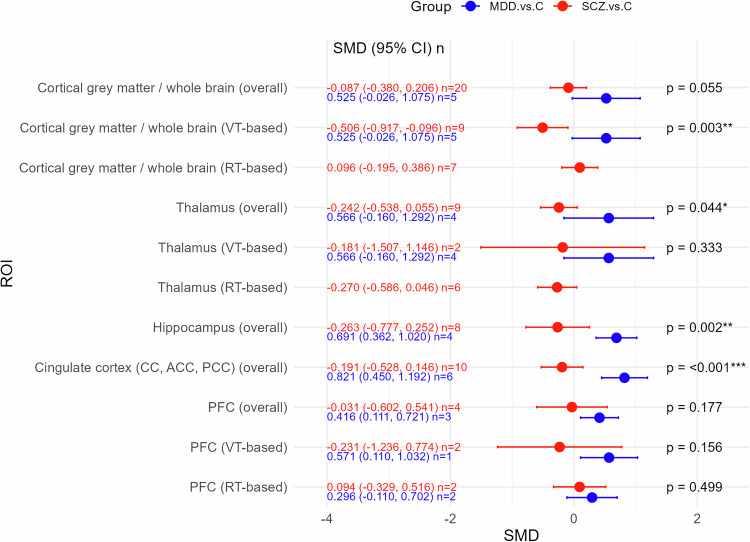
TSPO-PET signal across brain regions in MDD or SCZ vs. controls. This forest plot presents standardized mean differences (SMDs) and 95% confidence intervals (CIs) for TSPO-PET binding in patients with MDD (blue) and SCZ (red), each compared to healthy controls. Values represent meta-analytically derived effect sizes, separately calculated for each disorder and brain region. VT-based and RT-based quantification methods are shown where available. Most data were derived from De Picker et al. [8]. This meta-analysis did not cover the PFC. Therefore, we conducted a targeted PubMed search and calculated the respective original studies’ pooled effect sizes for the PFC. **Annotations:** p-values on the right reflect the statistical cross-diagnostic group comparisons (MDD vs. SCZ) for each ROI based on Z-tests. CI widths reflect precision, while directional differences highlight disorder-specific neuroimmune profiles. Asterisks denote significance levels: *p  <  0.05, **p  <  0.01, ***p  <  0.001.

Robust and reproducible increases in TSPO binding in MDD were found in frontolimbic structures (Fig. 4, Table 2, Suppl. Table 1, and Suppl. Figure 3). The hippocampus showed a moderate to large increase (SMD = 0.69, 95% CI: 0.36–1.02, p < 0.001), accompanied by low heterogeneity (I² = 18.4%, τ² = 0.021) and high precision (*v* = 0.092, rCIW=0.95). Similarly, a large increase was observed in the cingulate cortex (SMD = 0.82, 95% CI: 0.45–1.19, p < 0.001; I² = 37.6%, τ² = 0.081, *v* = 0.134, rCIW=0.90). A moderate increase was also observed in the PFC (SMD = 0.42, CI: 0.11–0.72, p = 0.007; I² = 0.0%, τ² = 0.000, *v* = 0.073, rCIW=1.47). In contrast, while SMDs for the thalamus and whole-brain cortical gray matter were numerically increased (0.57 and 0.53, respectively), these did not reach statistical significance and showed higher heterogeneity and wider rCIWs (thalamus overall: I² = 75.8%, τ² = 0.417, v = 0.133, rCIW = 2.57; cortical grey matter/whole brain overall: I² = 72.2%, τ² = 0.285, v = 0.110, rCIW = 2.10). VT-based quantification dominated the available MDD data and revealed consistently increased findings across brain regions. For example, PFC (VT‑based) had k = 1 with a significant increase (SMD = 0.57, p = 0.015, v = 0.055), while τ² was not estimable (k = 1). Reference tissue (RT)-based estimates were largely unavailable, precluding direct comparison of quantification methods within MDD.

These findings consistently implicate neuroinflammation in the hippocampus, cingulate cortex, and PFC, regions central to mood regulation and executive function.

#### TSPO-PET findings in BD

We identified only one PET study investigating bipolar disorder [98], which showed a significantly increased [(11)C]-(R)-PK11195 binding potential in the right hippocampus compared to healthy controls (p = 0.033) as well as a trend-level increase in the left hippocampus. Although the study provides evidence of a possible overlap in the brain circuits affected by neuroinflammation between MDD and BD, the TSPO-PET evidence base in BD remains too limited to draw broader conclusions.

#### TSPO-PET findings in SCZ

In SCZ, TSPO-PET imaging revealed a qualitatively distinct pattern (Fig. 4, Table 2, Suppl. Table 1, and Suppl. Figure 3). Across all examined brain regions, pooled effect sizes combining VT- and RT-based studies were consistently decreased but mostly non-significant. The only significant finding was a moderate reduction in TSPO binding in whole-brain cortical gray matter in VT-based studies (SMD= –0.51, 95% CI: -0.92 to -0.10, p = 0.016; I² = 66.5%, τ² = 0.264, v = 0.133, rCIW=1.62).

#### Cross-Diagnostic comparison of TSPO-PET findings

##### Between-group comparison of SMDs

Given that TSPO-PET evidence in BD is currently limited to a single study, quantitative cross-diagnostic comparisons were restricted to MDD and SCZ. To assess diagnostic TSPO differences between MDD and SCZ, we directly compared effect sizes across matched brain regions using Z-tests (Fig. 4, Table 2, Suppl. Table 1, and Suppl. Figure 3). This revealed significantly greater TSPO binding in MDD than in SCZ in the cingulate cortex (Z = 3.96, p < 0.001), hippocampus (Z = 3.06, p = 0.002), and thalamus overall (Z = 2.02, p = 0.044), with a trend for whole-brain cortical gray matter overall (Z = 1.92, p = 0.055). No significant differences were observed in the PFC overall (Z = 1.35, p = 0.177). Method‑matched contrasts were consistent: cortical grey matter (VT‑based) favored MDD (Z = 2.94, p = 0.003); thalamus (VT‑based) did not differ (Z = 0.97, p = 0.333); PFC showed no diagnostic differences in either VT‑ or RT‑based subsets (VT: Z = 1.42, p = 0.156; RT: Z = 0.68, p = 0.499).

##### Heterogeneity and within‑study precision (Descriptive)

To compare variability independent of quantification method, we first examined VT‑based data. Cortical grey matter (VT) showed broadly similar dispersion between diagnoses (SCZ: τ² = 0.264, I² = 66.5%, *v* = 0.133; MDD: τ² = 0.285, I² = 72.2%, *v* = 0.110). In the thalamus (VT), SCZ exceeded MDD (SCZ: τ² = 0.761, I² = 83.0%, *v* = 0.156; MDD: τ² = 0.417, I² = 75.8%, *v* = 0.133). In the PFC (VT), SCZ again showed markedly greater dispersion and lower precision (SCZ: τ² = 0.417, I² = 79.4%, v = 0.108; MDD: k = 1, τ² n.a., *v* = 0.055). Considering the overall (mixed‑method) pools, SCZ exceeded MDD by τ² in 4/5 ROIs (cortical grey matter, hippocampus, cingulate, PFC), while thalamus was the exception (MDD > SCZ by τ²); within‑study variance (*v*) was consistently higher in SCZ across VT‑based ROIs and in the overall pools, indicating lower study‑level precision in SCZ. Because τ² and *v* are approximated from I², k, and the pooled SE (see Supplementary Methods), these contrasts are descriptive and should be interpreted cautiously when k is small. Overall, the pattern suggests greater dispersion of true effects in SCZ than in MDD.

##### Interpretation and clinical relevance

Together with the SMD contrasts, the heterogeneity profile (τ²/I²) and the typical within‑study variance (*v*) indicate more variable TSPO-PET findings in SCZ than in MDD, particularly in VT‑matched PFC and thalamus. This is consistent with our framework in which microglial alterations represent one contributing, but not ubiquitous, pathway to psychosis and cognitive dysfunction, likely confined to specific subgroups rather than the entire diagnostic category. By contrast, MDD shows comparatively reproducible elevations in hippocampus and cingulate, aligning with a preferential engagement of microglia-related neuroinflammatory pathways in frontolimbic circuits. Within our framework, this pattern is compatible with stress‑ and mood‑related circuit vulnerability rather than global microglial activation. Nonetheless, residual methodological and sampling factors likely contribute and warrant cautious interpretation. Taken together, TSPO-PET findings map onto the brain regional level of our framework: they indicate where in the brain microglia‑related neuroinflammatory processes are most consistently involved, but do not, by themselves, specify whether microglia adopt a neurotoxic, neuroprotective, or primarily homeostatic phenotype. CSF and postmortem data are therefore essential to further constrain the underlying mechanisms.

### Microglial postmortem studies

Postmortem analyses resolve microglial phenotypes and regional marker expression with cellular specificity. Within our framework, this modality is critical for distinguishing altered microglial activation from changes in homeostatic identity (which can yield divergent results depending on marker choice), and for evaluating subgroup effects (e.g., suicidality) that may not be detectable in imaging or CSF studies.

#### Microglial postmortem findings in MDD

##### Microglial density and marker expression

Two systematic reviews summarized postmortem studies of microglia in MDD but did not conduct meta-analyses due to limited sample sizes and methodological heterogeneity [19, 27]. We extracted individual study data [19, 27], supplemented these with additional studies identified through our systematic search, and summarized all findings in Supplementary Table 2.

Across nine included studies, seven did not report a diagnosis-level increase in microglial density or activation in MDD [99–105]. Clark et al. [106] reported increased density of amoeboid IBA1+ microglia in the ventrolateral PFC, while Torres-Platas et al. [107] observed an increased primed/resting microglia ratio in the ACC despite no overall density change (findings related to suicidality in MDD are addressed later in the postmortem comparison).

##### Homeostatic and activated microglia

Two recent studies using brain tissue from the Netherlands Brain Bank [104, 105] reported elevated expression of homeostatic markers such as TMEM119, P2Y12, CX3CR1, and CCR5, but found no increase in classical inflammatory markers (e.g., HLA-DR, CD68, IL-6, IL-1β) [104, 105]. Snijders et al. observed downregulation of CD163 and CD14, suggesting preserved or even enhanced homeostatic microglial function in MDD [105].

##### Cytomorphology

The systematic reviews mentioned above [19, 27] lacked a systematic assessment of microglial morphology in MDD. Instead, most studies focused on density or gene expression.

#### Microglial postmortem findings in BD

##### Microglial density and marker expression

Three systematic reviews have summarized postmortem microglial findings in BD, but none performed quantitative meta-analyses [19, 108, 109]. We extracted and synthesized individual study data and summarized them in Supplementary Table 3. Among the fourteen eligible studies, only Rao et al. reported higher microglial markers in the frontal cortex (↑HLA‑DR+ microglia by qualitative assessment and higher CD11b expression) [110]. The remaining studies predominantly reported no diagnosis-level increases in microglial activation markers across key regions, including dorsolateral prefrontal cortex [100, 101, 111, 112], anterior cingulate/cingulate regions [100, 112–114], thalamus [100, 115], and hippocampus [100, 116] (Suicide-related findings are discussed later in the postmortem comparison).

##### Homeostatic and activated microglia

In BD, homeostatic microglial markers, including P2RY12, TMEM119, and CX3CR1, were generally unchanged at the cohort level [111, 112, 115, 116].

Several studies reported reduced expression of activation-associated markers rather than increases, including Seredenina et al. [113] (↓CD11b and CD68 microglia; ↔ IBA1) and Zhang et al. [112] (↓CD68 and ↓ TREM2 mRNA in DLPFC).

##### Cytomorphology

The systematic reviews of BD microglia did not systematically evaluate microglial morphology. Instead, most primary studies prioritized assessments of microglial density and/or marker expression, leaving cytomorphological phenotypes largely unexplored.

#### Microglial postmortem findings in SCZ

##### Microglial density and marker expression

Two meta-analyses addressed microglial density in SCZ with different conclusions (Supplementary Table 4). Van Kesteren et al. (11 studies; 181 SCZ, 159 controls) reported significantly increased microglial density (SMD = 0.69, 95% CI: 0.24–1.14, p = 0.003), especially in the temporal cortex (p = 0.033), but results were heterogeneous (I² = 68.6%) [25]. Conversely, Snijders et al. (12 studies; 238 SCZ, 252 controls) found no overall increase (g = 0.14, 95% CI: 0.10–0.39, p = 0.250, I² = 18.9%) [26]. In that meta-analysis, a significant increase in microglial density in the temporal cortex (Hedges’ g = 1.256, 95% CI: 0.55–1.96, p < 0.001) was only observed before outlier removal [26].

Although most studies focused on diagnostic group differences, limited evidence exists linking microglial alterations to clinical symptoms. One study reported that increased hippocampal HLA-DR+ microglial density was associated with greater severity of positive symptoms in SCZ [117].

##### Homeostatic and activated microglia

Van Kesteren et al. (14 studies; 330 SCZ, 323 controls) reported increased expression of proinflammatory cytokines (SMD = 0.37, 95% CI: 0.11–0.62, p = 0.005), particularly for IL-1β, IL-6, TNF-α, IL-10 and TGF-β, and cyclooxygenase (COX)-2, whose expression may reflect microglial activation [25]. In contrast, Snijders et al. (seven studies; 255 SCZ, 261 controls) found significant downregulation of microglial gene expression (e.g., TMEM119: g = –0.42, p < 0.001), suggesting loss of homeostatic function rather than classical activation [26].

##### Cytomorphology

Findings were inconsistent [26]. Some studies reported no changes [99, 118, 119], while others found inconsistent directional changes [26]. Additionally, some studies identified specific alterations, such as increased densities of both branched and amoeboid microglia [120] or more highly branched microglial projections [121]. Uranova et al. noted dystrophic microglial changes, particularly near oligodendrocytes in the prefrontal gray matter, including reduced mitochondrial density and increased lipofuscin granules, hallmarks of microglial degeneration [122].

##### Factors contributing to meta-analytic discrepancies

Methodological differences likely account for the different findings of the two SCZ meta-analyses. Van Kesteren et al. [25] used markers like HLA-DR and CD68, which label both microglia and infiltrating macrophages, potentially overestimating microglial activation. In contrast, Snijders et al. [26] included original data and applied microglia-specific markers (TMEM119, CX3CR1), finding downregulation of homeostatic genes, suggesting altered rather than activated microglial function. Van Kesteren et al. reported high heterogeneity (I² = 68.6%) [25], whereas Snijders et al. addressed potential outliers, which reduced heterogeneity (I² = 18.9%) [26].

#### Cross-Diagnostic comparison of microglial postmortem studies

Direct meta-analytic comparisons were not possible due to lack of pooled effect sizes in MDD and BD. However, qualitative synthesis reveals distinct profiles (Table 2, Suppl. Figure 3). In MDD, the overall picture of postmortem studies is marked by heterogeneity and lack of consistent microglial activation, and recent gene expression studies pointed to preserved or enhanced homeostatic function with limited evidence for overt immune activation [104, 105].

In contrast, SCZ shows either increased microglial activation or disrupted homeostasis, particularly in the temporal cortex and PFC [25, 26]. This includes increased density, proinflammatory gene expression, or downregulation of core microglial genes.

Across nine MDD studies, most did not report a diagnosis-level increase in microglial density or activation markers, with exceptions indicating region- or phenotype-specific alterations (e.g., increased amoeboid Iba1+ microglia in ventrolateral PFC and increased primed/resting microglia ratio in ACC). In addition, two recent studies suggested a predominantly non-inflammatory / homeostatic microglial profile in MDD, characterized by higher expression of homeostatic markers without increased classical inflammatory markers [104, 105].

In BD, a similar overall pattern emerged across fourteen studies: only Rao et al. [110] reported increased microglial activation markers in frontal cortex, whereas the remaining studies predominantly reported no diagnosis-level increases across key regions including the PFC, cingulate, thalamus, and hippocampus. In contrast, SCZ meta-analyses indicate either increased microglial density and inflammatory gene expression [25] or reduced expression of mature/homeostatic microglial markers without increased classical activation markers [26], with region-specific signals particularly discussed for temporal cortex and prefrontal/cingulate regions.

These findings indicate that microglial alterations in MDD and BD are subtle and often non-inflammatory at the diagnosis level, whereas SCZ shows more pronounced and/or divergent microglial alterations (activation and/or loss of homeostatic signature), possibly contributing to synaptic pruning deficits and circuit dysconnectivity as key elements in SCZ pathophysiology. This divergence supports disorder-specific microglial phenotypes within our framework: subtle homeostatic/compensatory adaptations in MDD and BD versus dysfunctional/activated states in SCZ, although standardized, multi-regional studies with single-cell resolution are needed to definitively characterize these profiles.

An additional cross-diagnostic topic is the influence of suicidality on microglial findings. Several studies indicate that microglial differences in mood disorders become more apparent after stratifying by suicide status (Supplementary Tables 2–4). For example, Steiner et al. [100] reported no overall diagnosis-level difference in HLA‑DR+ microglial density in MDD, BD and SCZ, but an increase in suicidal cases in the DLPFC, ACC, and mediodorsal thalamus. Brisch et al. [99] reported no overall diagnosis-level difference in dorsal raphe HLA‑DR+ microglial density, but a decrease specifically in non-suicidal patients with MDD or BD. In BD, suicide-stratified findings were region- and marker-specific. Naggan et al. [116] observed increased hippocampal P2RY12+ microglial density only in suicidal BD, whereas Petrasch‑Parwez et al. [118] and Zhang et al. [112] reported reductions confined to non-suicidal subgroups (e.g., lateralized/region-specific Iba1 effects in aMCC and reduced P2RY12 in non-suicidal BD in DLPFC, respectively). Notably, the major SCZ postmortem meta-analyses did not examine suicide as a moderator, limiting cross-diagnostic conclusions for SCZ [25, 26].

Overall, the available evidence supports suicide status as an important moderator of postmortem microglial findings, with effects that appear to be region- and marker-dependent rather than uniformly reflecting a single “activated” microglial state across diagnoses.

### Microglia-related KYN pathway metabolites and enzymes

The KP links immune activation to glutamatergic neurotransmission, with microglia and astrocytes regulating the balance between endogenous NMDA receptor agonists (e.g., QUIN) and antagonists (e.g., KYNA) [13]. Microglia primarily generate QUIN and 3-HK [73], while astrocytes synthesize KYNA [78].

We prioritized CSF studies over blood-based ones due to more direct reflection of brain-specific KP activity. Peripheral metabolites have limited relevance to microglial pathology [123–125]. For instance, only KYN and 3-HK reliably correlated between peripheral and central compartments, whereas KYNA and TRP showed inconsistent correlations [126]. In our review, we excluded several meta-analyses due to methodological limitations, such as mixing diagnostic categories [127, 128], combining postmortem brain and CSF data (which precludes region-specific conclusions) [126–131], or failing to separate central and peripheral compartments when analyzing KP metabolites [132, 133].

#### Kynurenine pathway studies in MDD

##### CSF findings

Inam et al. (11 studies; 217 MDD, 254 controls) found no significant changes in CSF levels of KYNA, KYN, or TRP. For QUIN, a non-significant trend towards higher levels was observed (SMD = 2.66, CI: −1.04 to 6.35, rCIW=2.78, p = 0.159, I² =98%, k = 2; n = 136), but the small evidence base precludes firm conclusions (Supplementary Table 5) [134]. Other metabolites (3-HK, 3-HAA, PIC) were not systematically analyzed.

##### Postmortem brain findings

Six postmortem studies met inclusion criteria, with inconsistent regional findings and methodological heterogeneity limitations (Supplementary Table 6). QUIN immunoreactivity showed a regional dissociation: Steiner et al. analysed acutely depressed suicide cases (MDD and BD combined) and reported increased QUIN-immunoreactive microglia in the subgenual ACC (sACC) and anterior midcingulate cortex (aMCC) versus controls; post‑hoc analyses indicated that these increases were driven by the MDD subgroup (and were not significant in BD) [74]. In the hippocampus, Busse et al. reported reduced QUIN‑immunoreactive microglia in depressed cases, with post‑hoc tests showing decreased right CA1 QUIN‑positive microglia in both MDD and BD [135]. Beyond QUIN, evidence was limited and partly depended on diagnostic coverage. In the ACC, Miller et al. reported increased numbers of TDO2-positive astrocytes in white matter [136], while a follow-up metabolite analysis in the same ACC cohort found no differences in TRP, 3-HAA, or nicotinamide (NAM) [137]. In ventrolateral PFC tissue from individuals with depressive disorder not otherwise specified, Clark et al. reported decreased QUIN, a reduced KYN/TRP ratio, and lower expression of IDO1, IDO2, and TDO2 [106]. Brown et al. reported sex-specific alterations in ACC (BA24), with reduced KYNA in females and increased KAT II expression [138].

Taken together, postmortem KP findings in depressive disorders point to potentially region-dependent QUIN/KYNA alterations involving both upstream enzyme regulation (IDO/TDO2) and astrocytic KYNA synthesis, but the small number of studies and diagnostic heterogeneity limit firm conclusions.

#### Kynurenine pathway studies in BD

##### CSF findings

Inam et al. (6 studies; 246 BD, 340 controls) reported no significant differences in CSF TRP; KYNA showed a non-significant trend towards higher levels (SMD = 1.40, CI: −0.28 to 3.09, rCIW=2.41, p = 0.103, I² =98%, k = 2; n = 377), but the evidence was limited (Supplementary Table 5) [134]. KYN and QUIN were not systematically analyzed.

##### Postmortem brain findings

Five postmortem studies met inclusion criteria, marked by variable regional results and methodological differences (Supplementary Table 7). Although Steiner et al. reported increased QUIN‑immunoreactive microglia in the sACC and aMCC when analysing all depressed cases together (see above, MDD + BD vs controls), post‑hoc analyses did not show a significant difference between the BD subgroup and controls [74]. By contrast, Busse et al. reported a reduction in the number of QUIN-immunoreactive microglia in the hippocampus, not only in MDD but also in BD (most clearly in right CA1) [74, 135].

In addition, Miller et al. detected elevated KYN levels and increased TDO2-positive astrocytes were found in the ACC, whereas other metabolites, including TRP, 3-HAA, and NAM, did not differ between BD and control samples [136, 137]. Finally, Lavebratt et al. [139] reported reduced KMO expression in the prefrontal cortex of BD patients with psychotic features, implicating reduced microglial conversion of KYN to QUIN in a clinically relevant subgroup.

Overall, the BD postmortem literature suggests regionally and phenotype-dependent KP alterations. While evidence for QUIN alteration in BD is limited and inconsistent, there is support for upstream activation (elevated KYN/TDO2) alongside potential downstream constraints at KMO in psychotic BD.

#### Kynurenine pathway studies in SCZ

##### CSF findings

CSF studies in SCZ show more robust KP alterations (Supplementary Table 5). Inam et al. (8 studies; 238 SCZ, 240 controls) reported significantly increased KYNA levels (SMD = 2.64, 95% CI: 1.16–4.13, rCIW=1.13, p < 0.001, I² = 96%, k = 6, n = 384), indicating a large effect but extreme heterogeneity [134]. Similarly, Rømer et al. found increased KYNA (SMD = 1.58, 95% CI: 0.34–2.81, rCIW=1.56, p = 0.013, I² = 96%, k = 6, n = 549) and KYN (SMD = 1.00, 95% CI: 0.64–1.36, rCIW=0.72, p < 0.001, I² = 17%, k = 3, n = 113), while TRP and other KP metabolites were not systematically analyzed [11].

##### Postmortem brain findings

Ten postmortem studies met inclusion criteria (Supplementary Table 8), spanning hippocampus, ACC, and several prefrontal cortical regions. Across cortical regions, multiple studies reported elevated KYNA levels in SCZ [80, 140–142], whereas two studies found no significant differences [136, 143]. KYN elevations were reported in ACC [136], in BA9/BA19 (but not BA10) [80], and in DLPFC gray and white matter [140]. Additional metabolite changes were reported in specific regions (e.g., increased TRP and 3-HAA in ACC [137]; increased 3-HK, anthranilic acid, and 3-HAA in DLPFC gray and white matter [140]).

QUIN findings were region-dependent: Gos et al. reported decreased QUIN immunoreactivity in hippocampal CA1 [144], Antenucci et al. observed increased QUIN selectively in DLPFC white matter (alongside increased KYNA in white matter) [140], and Afia et al. found a similar trend in the DLPFC grey matter, although this was not significant (p = 0.07) [143].

Enzyme measures suggest dysregulation at multiple steps. Upstream, TDO2/TDO expression was increased in anterior PFC white matter [145], ACC [136], and DLPFC (including the high-inflammation subgroup) [141, 142]. Findings for KMO were mixed: reduced KMO activity/expression was reported in BA9/BA10 [142] and BA6 [146], whereas Afia et al. reported increased KMO protein in DLPFC [143]. Notably, the only study assessing 3-HAO activity reported a reduction in BA9 (but not BA10) [142]. KAT enzymes also showed context-dependent changes, with increased KAT I and KAT II in the high-inflammation subgroup [141] but reduced KAT II in another DLPFC cohort [143].

In sum, postmortem SCZ studies support a broadly dysregulated KP with region- and inflammation-dependent shifts that often favor KYNA accumulation, while QUIN changes appear dissociated across hippocampal versus cortical compartments.

#### Cross-Diagnostic comparison of Kynurenine pathway studies

##### CSF findings

SCZ is characterized by increased KYNA and KYN [11, 134], while MDD shows no consistent alterations (Table 2, Suppl. Figure 3). In BD, CSF TRP was unchanged and KYNA showed a non-significant trend towards higher levels; KYN and QUIN have not been systematically meta-analysed in BD [134]. These findings align with magnetic resonance spectroscopy (MRS) data showing reduced glutamate/glutamine in MDD [147] and increased levels in SCZ [148], supporting disorder-specific NMDA receptor modulation.

Mapping onto our framework, elevated CSF KYNA/KYN in SCZ indicates sustained activation of the astrocyte-dominated KP branch favoring KYNA synthesis, which may contribute to NMDA receptor hypofunction, a core element of psychosis pathophysiology [79, 149]. The relative absence of CSF KP alterations in MDD and BD suggests that any microglial QUIN production occurs locally within circuits rather than systemically, consistent with the region-specific TSPO elevations observed in frontolimbic structures.

##### Postmortem brain findings

While MDD, BD, and SCZ all show KP alterations, the pattern is diagnosis-, region-, and phenotype-dependent [74, 135] (Table 2, Suppl. Figure 3).

Across mood disorders, hippocampal CA1 QUIN immunoreactivity is reduced in both MDD and BD [135]. By contrast, QUIN-immunoreactive microglia are increased in the sACC and aMCC in MDD but not BD [74]. Notably, ACC upstream KP activation emerges as a shared MDD-BD feature, with increased TDO2 and elevated KYN [136, 137]. In BD, this pattern is amplified in psychotic cases and paired with reduced KMO expression in the prefrontal cortex [136, 139], limiting downstream QUIN production.

In SCZ, the most replicated postmortem signature is a cortical shift toward KYNA accumulation: several studies report elevated KYNA (often with increased KYN and/or KYN/TRP ratio) [80, 140–142] and upregulated upstream enzymes (TDO/TDO2) [136, 141, 142, 145]. QUIN findings in SCZ remain region-specific, with decreased hippocampal CA1 QUIN immunoreactivity reported alongside increased QUIN in DLPFC white matter [140, 143, 144].

These patterns map directly onto framework predictions: MDD shows circuit-specific, microglia-mediated QUIN elevations in stress-relevant regions (ACC), potentially contributing to local glutamatergic excitotoxicity and affective symptoms [70], while reduced hippocampal QUIN may reflect compensatory processes [135]. In SCZ, and potentially in psychotic BD, more pervasive astrocyte-KP dysregulation favoring KYNA accumulation, aligns with prefrontal NMDA hypofunction and cognitive/psychotic features, suggesting a KP phenotype that may track psychosis-related biology across diagnostic boundaries [58, 66, 150].

However, the postmortem evidence base remains limited by small and heterogeneous samples, medication or suicide effects, underscoring the need for harmonized, higher-powered studies that stratify by inflammatory phenotypes and clinical subtypes while profiling KP metabolites and enzymes across matched gray and white matter regions.

## Methodological considerations

### Microglial PET studies

PET imaging enables in vivo assessment of neuroinflammatory activity, offering a perspective that complements the spatial detail of postmortem studies. However, TSPO-targeted PET methods face interpretability and generalizability challenges. Although widely used to infer microglial activation, TSPO is also expressed in astrocytes, endothelial cells, and sometimes neurons [151], making it a marker of general neuroinflammation rather than microglia-specific changes. Elevated binding may also reflect myeloid proliferation or peripheral monocyte recruitment, adding interpretive uncertainty [152, 153].

Across MDD, BD and SCZ, TSPO-PET studies are further limited by small sample sizes and methodological heterogeneity, including tracer and quantification differences. First-generation tracers have high non-specific binding and low sensitivity; second-generation tracers improve signal-to-noise but require genetic stratification due to TSPO rs6971 polymorphisms. In MDD and SCZ, some datasets include overlapping cohorts, and for BD, the TSPO PET evidence base currently comprises only one study [98]. These factors contribute to study variability, especially in SCZ, where findings are more inconsistent, possibly due to variation in disease stage, antipsychotic use, subgroups or technical factors. TSPO-PET cannot distinguish microglial activation states or resolve subregional/layer-specific changes due to limited spatial resolution.

To improve specificity and interpretability, future PET studies should: (1) develop tracers distinguishing activation states; (2) combine PET with complementary modalities (e.g., MRS, diffusion tensor imaging (DTI)), validated via postmortem studies; and (3) standardize acquisition and analysis protocols. Meta-analyses aid synthesis [8, 27, 94, 96, 97], but heterogeneity remains a major limitation. Overcoming these challenges is key to advancing diagnostics and mechanistic understanding in MDD, BD and SCZ.

### Postmortem studies: microglial and kynurenine pathway markers

Postmortem studies enable precise cellular localization of neuroimmune and metabolic changes using techniques such as immunohistochemistry, in situ hybridization, transcriptomics, and mass spectrometry, offering insights not achievable in vivo. These methods complement PET by capturing chronic-stage pathology in MDD, BD and SCZ.

However, the reviewed postmortem studies face reproducibility, generalizability, and interpretability issues. Limited study numbers and marker/region/method heterogeneity mean meta-analyses exist only for microglial changes in SCZ [25, 26], and none exist for MDD and BD. Likewise, KP alterations in MDD, BD and SCZ lack quantitative synthesis due to variability in metabolites, enzymes, and brain regions studied.

Most studies are small, single-center, and vary in markers and methods. Regional bias is common, with most focusing on predefined areas (e.g., ACC, PFC, hippocampus), differing by disorder. MDD studies often focus on ACC and medial PFC, while BD studies have similarly prioritized PFC and cingulate regions. SCZ studies more often target DLPFC, reflecting historical trends rather than comprehensive assessments. This limits whole-brain inference and may obscure disorder-overlapping patterns. Most use single-marker approaches; few apply high-dimensional methods like spatial transcriptomics or multiplex immunophenotyping.

Confounders (postmortem interval, pH, medication, chronicity, mood state and clinical subtype - e.g., psychotic features, suicide status) are inconsistently controlled and may mask disease-specific signals. Suicidality may independently affect microglial markers and should be considered. Postmortem tissue also reflects late-stage pathology, complicating causal inference.

To improve interpretability, future studies should: (1) adopt wholebrain approaches to reduce bias; (2) use multi-marker strategies to improve phenotypic resolution; (3) harmonize tissue protocols, markers, and analysis pipelines across centers; and (4) stratify by sex, suicide status, illness stage and clinical subtype (e.g., psychotic features) where available.

### CSF studies on kynurenine pathway metabolites

CSF analysis enables evaluation of central KP metabolites, bridging peripheral and CNS-specific immune changes. Unlike postmortem studies, CSF sampling permits in vivo and longitudinal assessments.

Despite these advantages, current CSF studies are limited by small sample sizes, low replication, and narrow metabolite coverage. Meta-analyses exist for key KP metabolites across MDD, BD and SCZ (e.g., KYNA, QUIN, TRP), but data on intermediates like 3-HK, 3-hydroxyanthranilic acid (3-HAA), and picolinic acid (PIC) remain sparse [134].

Variability in CSF collection, storage, quantification, and assay sensitivity complicates comparisons. Patient-related confounders like medication, disease stage, moodpsychosis state, inflammation, demographics, were inconsistently controlled, obscuring disease-specific effects.

Longitudinal CSF studies are lacking, limiting insights into whether KP alterations reflect stable traits, state-dependent shifts, or treatment/inflammation responses.

To advance the field, future CSF studies in MDD, BD and SCZ should: (1) increase sample sizes and replicate findings, especially for underexplored metabolites; (2) broaden metabolic scope and integrate with enzyme/pathway measures; (3) standardize protocols to reduce variability; (4) control key confounders systematically; and (5) incorporate longitudinal designs to capture temporal dynamics.

## Clinical translation of findings

### Microglia-specific in vivo biomarkers for precision diagnostics

Identifying subgroups of patients with affective or psychotic symptoms who may benefit from microglia-targeted treatments requires better understanding of disease heterogeneity [154]. Roughly 30% of MDD patients show low-grade peripheral inflammation [31], with melancholic and atypical subtypes showing distinct immune profiles [155], suggesting heterogeneous peripheral-to-central immune signaling that may differentially engage frontolimbic microglia. In BD, peripheral inflammatory markers (e.g., C-reactive protein [CRP], TNF-α, IL-6) are also elevated and show mood-state associated variability [156]. Similar inflammatory subgroups exist in SCZ [157, 158], likely reflecting heterogeneous developmental trajectories and variable microglial dysregulation in prefrontal and temporal circuits. Yet, more specific in vivo methods are needed to detect CNS-specific microglial activation or changes in phenotype:

- PET imaging – While TSPO tracers lack microglial specificity and bind astrocytes and other cells, they remain the primary tool for assessing circuit-level neuroinflammation [159]. Development is ongoing for more specific targets, such as COX-1/2, P2X purinoceptor 7, cannabinoid receptor 2, β-glucuronidase, inducible nitric oxide synthase, and colony-stimulating factor 1 receptor [159, 160], as well as KP enzymes [161] and P2Y12, a microglia-specific receptor [162]. However, these remain largely untested in MDD, BD or SCZ. In BD, TSPO-PET evidence is currently limited to a single small study reporting increased hippocampal binding [98]. New PET ligands targeting synaptic proteins [e.g., synaptic vesicle glycoprotein 2 A (SV2A)] show promise; SV2A was significantly reduced in SCZ patients, possibly reflecting excess synaptic pruning [163]. Such tracers may enable early intervention in prodromal stages.
- Extracellular vesicles (EVs) – Microglia-derived EVs in CSF and blood carry microglia-specific proteins (e.g., TMEM119, P2RY12), miRNAs, and signaling molecules reflective of activation states [164–166]. Specific isolation of microglia-derived EVs is possible using markers like CD11b, TMEM119, and CD45low [165, 167]. Despite their potential to discriminate between homeostatic/compensatory (MDD) and activated/dysregulated (SCZ and potentially psychotic BD subgroups) microglia states, systematic studies in MDD, BD or SCZ are lacking.
- CSF KP metabolite alterations - show strongest evidence for KYNA in SCZ, whereas MDD and BD findings are smaller and more heterogeneous (e.g., trend-level QUIN increases in MDD and KYNA increases in BD), consistent with variable microglial/astroglial KP engagement across diagnoses [10, 11, 134], but require systematic validation.

Clinical features may aid patient stratification. Individuals with treatment-resistant depression (including bipolar depression) or SCZ or comorbid immune conditions may benefit more from immunomodulatory approaches [168–170].

### Potential microglia-targeted therapies

Our findings highlight that microglial and immune alterations may not align strictly with diagnostic labels, but rather with certain psychopathological dimensions or subgroups. For clinical translation, this means that interventions targeting microglia-mediated inflammation might benefit patients across diagnostic categories (e.g., patient subsets with high inflammatory biomarkers or prominent cognitive symptoms, regardless of MDD, BD or SCZ diagnosis). Adopting a dimensional or precision psychiatry approach could enhance development of microglia-targeted therapies by focusing on biologically defined subgroups instead of broad diagnoses [171, 172]. With this in mind, some treatments target microglia more directly than general anti-inflammatory drugs:

- Minocycline reduces pro-inflammatory cytokines and microglial activation. A meta-analysis demonstrated that minocycline improved negative symptoms in SCZ, but not positive symptoms or cognition [173, 174]. In MDD it had modest antidepressant effects without improving anxiety or quality of life [173]. In BD, early proof-of-concept work suggested possible benefit in an inflammation-related subgroup, but a larger randomized trial found no evidence for efficacy of adjunctive minocycline in bipolar depression. However, there were indications of a better response at higher IL-6 levels [175, 176].
- COX-2 inhibitors (e.g., celecoxib) have shown promise due to microglia-driven COX-2 and prostaglandin E2 upregulation [177, 178]. Meta-analyses support potential use in some psychiatric disorders. In SCZ, celecoxib improved symptoms in first-episode cases but not in chronic ones [179]. It has shown antidepressant effects in MDD as adjunct therapy [180, 181]. In BD, adjunct celecoxib has shown mixed results across mood states, with early small trials suggesting benefit in mania or depressive/mixed episodes, while a larger bipolar depression trial was negative [176, 182, 183]. However, since COX-1 is also involved in neuroinflammation [178], further research is needed to determine which type of COX inhibitor is most effective.
- TNF-α inhibitors (e.g., infliximab, etanercept) reduce microglial activation by targeting TNF-α. Treatment-resistant MDD patients with elevated CRP, TNF-α, and TNF soluble receptors benefited from infliximab [184]. In BD, adjunctive infliximab did not reduce depressive symptoms overall, but secondary analyses suggested possible benefit in a trauma-exposed subgroup, again underscoring the importance of stratification [185]. Adalimumab improved negative and general symptoms in SCZ, but not positive symptoms [186], possibly by preserving cortical connectivity.
- KP modulators (e.g., KAT II inhibitor PF-04859989) improved cognition in rodent SCZ models [187]. IDO and TDO inhibitors (e.g., TRP analogs, amino heterocycles) reduced QUIN and 3-HK formation in animal models [188–191], potentially effective in patients with MDD.
- Peroxisome Proliferator-Activated Receptor (PPAR)-γ is highly expressed in microglia and shows potent anti-inflammatory effects [192], and PPAR agonists have demonstrated positive effects in MDD and SCZ [193, 194], and in bipolar depression in a small adjunctive trial [195].
- N-acetylcysteine likely suppresses microglial inflammation [196] and has reduced symptoms in SCZ and bipolar depression when used as augmentation therapy [174, 197].
- CSF1R inhibitors, which regulate microglial proliferation, are under investigation [198].
- The P2X7 receptor antagonist JNJ-54175446 had no effect on mood but reduced anhedonia in MDD patients [199].
- Sphingosine-1-phosphate (S1P) receptor modulators (e.g., fingolimod) inhibit microglial activity; a trial in SCZ showed tolerability but no efficacy, which may reflect the need for patient stratification [200].
- EVs may eventually be used to reprogram microglial phenotypes in subgroups of patients with MDD away from QUIN-driven states or in SCZ and BD (particularly psychosis- or inflammation-associated subgroups) toward phenotypes that restore cortical circuit support and KP balance, as early animal studies suggest [201–203].

As our framework emphasizes that microglial changes are not uniform across brain regions, targeted delivery is essential. These include intranasal drug administration for cortical and limbic modulation [204], and engineered EV-based delivery approaches that can modulate microglial phenotypes[201–204]. These therapies hold promise, but clinical efficacy remains uncertain. Future research should focus on identifying the subgroups most likely to benefit.

## Limitations, conclusions and future directions

### Limitations

While we included supplementary analyses on BD across TSPO-PET, postmortem microglial markers, and KP alterations, the available evidence base remains sparse and heterogeneous, particularly for TSPO-PET (k = 1 study). In addition, many BD and MDD studies do not consistently report mood state (euthymic vs depressed vs manic/mixed), psychotic features, medication exposure, or inflammatory status at sampling, limiting phase-specific and subgroup-specific inference.

A major limitation of the extant human literature, and thus of this review, is the predominant reliance on categorical DSM/ICD diagnoses. Most studies compare “patients vs controls” within a single diagnostic category and rarely quantify symptom dimensions (e.g., severity of psychosis, affective symptom burden, cognition) in a way that enables testing whether microglia-related alterations track continuous levels of psychopathology across disorders. This limitation likely contributes to inconsistency in SCZ findings, where variability in psychosis severity, illness stage, and inflammatory subtype may differ markedly across cohorts. Future research should therefore adopt transdiagnostic designs across the affective–psychosis spectrum, with dimensional phenotyping and biologically informed stratification.

Another limitation concerns sex differences. MDD is more prevalent in females, and there is evidence that sex may influence stress responsivity and immune–microglia signaling [205, 206]. However, the majority of studies included in this review did not disaggregate results by sex (or gender-related variables), limiting our ability to evaluate sex-specific patterns across MDD, BD and SCZ. We therefore encourage future research to incorporate sex-stratified analyses and to consider hormonal status and other sex-related moderators when examining microglial and immune phenotypes, as this may inform more personalized therapeutic approaches.

Finally, even a multimodal synthesis cannot resolve all mechanisms by which immune dysfunction shapes brain function. TSPO is not specific to microglia, CSF KP measurements provide only indirect evidence regarding cellular sources, and postmortem tissue reflects cross-sectional end-stage snapshots that can be influenced by agonal state and treatment exposure. Moreover, with respect to neurotransmission, the reviewed human CSF and postmortem evidence primarily informs the KP–glutamate/NMDA interface; other neurotransmitter systems implicated in affective psychopathology and psychosis (e.g., dopamine or GABA) are not directly interrogated by the modalities summarized here. Future studies should therefore combine microglia biomarkers with transmitter-specific approaches (e.g., dopaminergic PET, magnetic resonance spectroscopy) and longitudinal designs to better constrain mechanism.

### Conclusions and future directions

Building on the a priori integrative framework (Table 1), we synthesized TSPO-PET, CSF KP, and postmortem microglial/KP evidence in the section on Comparative analysis of microglial involvement in MDD, BD and SCZ, and summarized the modality-specific findings in Table 2. A graphical overview of this cross-diagnostic multimodal synthesis is provided in Suppl. Figure 3. Mapping these results back onto the framework closes the loop and highlights a small set of evidence-weighted patterns across the affective–psychosis spectrum, while underscoring substantial heterogeneity that is more consistent with dimensional and subgroup effects than categorical diagnosis alone.

At the macro-scale level of brain regional neuroimmune engagement (TSPO-PET; Fig. 4), MDD shows the most reproducible in vivo signal, with increased TSPO binding in frontolimbic regions (cingulate cortex, hippocampus, prefrontal cortex). In SCZ, TSPO-PET findings are substantially more heterogeneous, with pooled estimates that generally suggest either no clear case–control difference or small decreases, depending on the region and analytic approach. For BD, the TSPO PET evidence is currently limited to a single small study. At the tissue level, postmortem studies in mood disorders (MDD/BD) most often report no diagnosis-level differences in microglial density or classical activation markers, although emerging MDD data point to subtle, non-classical and/or homeostatic shifts rather than robust inflammatory activation. In SCZ, postmortem signatures are divergent and marker-dependent, ranging from activation-marker increases to reduced expression of homeostatic microglia markers, consistent with microglial dysregulation rather than a unitary “activated” state. At the immune–neurotransmission interface, the most consistent biochemical phenotype appears in SCZ, where CSF studies converge on elevated KYNA (and KYN) and postmortem studies frequently indicate cortical KYNA accumulation. By contrast, KP findings in MDD and BD remain sparse and sometimes regionally dissociated (including cingulate versus hippocampal QUIN differences in single cohorts).

**Fig. 5:**
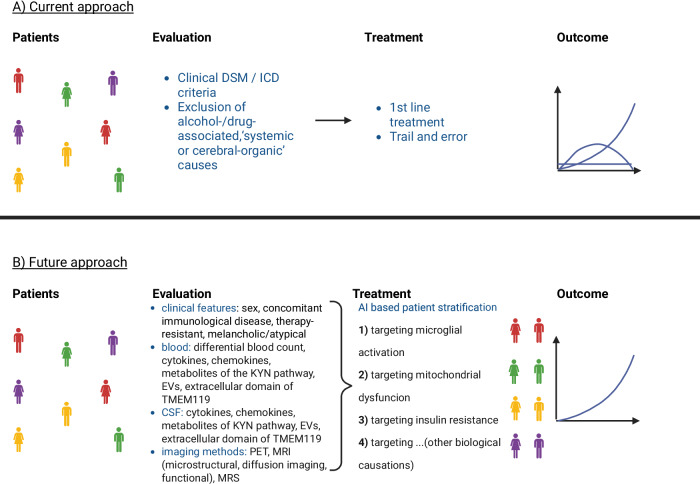
Current and future diagnostic and therapeutic approaches in psychiatry. Panel **A**: In current clinical practice, patients with major depressive disorder (MDD) and schizophrenia often receive similar standard-of-care treatments based on DSM/ICD criteria, following the exclusion of secondary causes. This approach relies heavily on first-line interventions and trial-and-error strategies, resulting in highly variable outcomes [169, 170, 223]. Panel **B**: In contrast, a future model may leverage a combination of clinical phenotyping (e.g., treatment resistance, subtype, comorbidities), biomarker-based stratification (blood and CSF markers such as cytokines, kynurenine pathway metabolites, extracellular vesicles, TMEM119), and advanced neuroimaging (e.g., PET, MRI, MRS). These data could be integrated using artificial intelligence to identify biologically defined subgroups. Targeted interventions might then address specific pathophysiological mechanisms, such as microglial activation, mitochondrial dysfunction, or insulin resistance, potentially leading to more robust and consistent treatment outcomes [172] (Created in BioRender. Steiner, J. (2026) https://BioRender.com/4wk8slb).

Taken together, these findings suggest two cross-diagnostic organizing principles: First, substantial clinical and biological heterogeneity (including psychosis burden, suicidality, illness stage, medication exposure, and inflammatory biotypes) likely explains a meaningful portion of between-study inconsistency, consistent with the marked dispersion and variable effect patterns evident in the TSPO-PET summary (Fig. 4). Second, microglia- and KP-related alterations may align more closely with symptom dimensions and biologically defined subgroups than with diagnosis. Realizing the translational potential of this field will require transdiagnostic, dimensionally phenotyped cohorts, longitudinal and interventional designs, and validated microglia-selective biomarkers that can be deployed in biomarker-enriched, mechanism-focused trials. Figure 5 provides a pragmatic roadmap for transdiagnostic stratification and treatment matching, an essential step for advancing precision immunopsychiatry in affective and psychotic disorders [171].

## Supplementary information

Supplementary Material

## Data Availability

Online supplementary material.

## Footnotes

1 In this context *“homeostatic“* refers to the state in which microglia maintain their normal, balanced functions like routine surveillance essential for CNS health, without being activated by injury, disease, or other pathological conditions.
