# Supplementary material for: Multimodal microglial and kynurenine pathway alterations across the affective-psychosis spectrum: a systematic review of patterns, heterogeneity, and dimensional implications
Source: Mol Psychiatry. 2026 Apr 21;31(9):5242–64. doi: 10.1038/s41380-026-03614-3 (PMC13442045; doi:10.1038/s41380-026-03614-3)
Supplement: Supplementary file 1 [file 41380_2026_3614_MOESM1_ESM.docx]

**Supplementary Methods**

## 1. Literature Search Strategies, Screening, Study Selection, and Data Extraction

**a) Overview**

We conducted a comprehensive literature search using two complementary strategies. First, we performed an unsystematic, targeted literature scan to inform the narrative sections of the main article. Second, we carried out a systematic, PRISMA-guided search and review process to support the quantitative synthesis.

**b) Systematic Literature Search for the Quantitative Synthesis**

Our systematic search and study selection followed a PRISMA-compliant workflow across PubMed, Scopus, and Web of Science, focusing exclusively on human studies examining microglial alterations in MDD and SCZ. The PRISMA flow diagram is provided as **Supplementary Figure 1**, detailing the study inclusion process for major depressive disorder (MDD) and schizophrenia (SCZ).

The Boolean search string used was: *("schizophreni*" OR "psychosis") OR ("depress*" OR "suicid*")) AND ("microglia*" OR "translocator protein" OR "TSPO" OR "PK11195" OR "kynuren*" OR "quinolin*") AND ("positron emission tomography" OR "PET" OR "brain tissue" OR "postmortem" OR "post-mortem" OR "immunohistochem*" OR "morphomet*" OR "cerebrospinal fluid" OR "CSF").*

All searches were last updated on July 5, 2025. This search yielded 142 original case–control studies [1-142] and 31 relevant systematic reviews or meta-analyses [143-173].

We performed an additional supplementary systematic literature search on bipolar disorder (BD) with the following Boolean search string: *("bipolar disorder" OR "bipolar depress*" OR "affective psychos*" OR mania OR manic OR "bipolar I" OR "bipolar II" OR cyclothymi*) AND ("microglia*" OR "translocator protein" OR "TSPO" OR "PK11195" OR "kynuren*" OR "quinolin*") AND ("positron emission tomography" OR "PET" OR "brain tissue" OR "postmortem" OR "post-mortem" OR "immunohistochem*" OR "morphomet*" OR "cerebrospinal fluid" OR "CSF").* The PRISMA flow diagram for the BD search is provided as **Supplementary Figure 2**.

During screening of BD studies, we systematically evaluated whether identified papers also included MDD or SCZ cohorts; such studies were cross-referenced with the primary MDD/SCZ search results to ensure complete ascertainment. These supplementary searches were performed on January 13, 2026, yielding 37 eligible publications (27 original case-control studies and 10 systematic reviews/meta-analyses; **Supplementary Figure 2**).

**c) Eligibility Criteria and Reasons for Exclusion**

*Inclusion criteria for the systematic review* were human case–control studies of MDD or SCZ, and BD in the supplementary literature search, that reported quantitative data on at least one of the following: TSPO PET imaging, postmortem microglial immunohistochemistry or morphometry, or cerebrospinal fluid (CSF) kynurenine measurements.

*We excluded studies for the following reasons (with counts recorded):* animal-only studies; case reports; articles lacking original data; focus on pediatric or adolescent psychiatry; studies not investigating MDD, SCZ or BD; mixed-diagnosis samples; in vitro/ex vivo designs; cross-sectional comparisons of multiple psychiatric disorders without a control group; absence of analysis or quantification of microglia, TSPO, or kynurenines; quantification of kynurenines only in peripheral blood; and studies assessing only cytokines.

**d) Screening Workflow and Adjudication**

Four reviewers (MN, LD, LSA, GML) independently performed the title/abstract and full-text screening in duplicate, with a fifth author (JS) supervising. Discrepancies were resolved in adjudication meetings until consensus was achieved. For each included study, two reviewers independently extracted key study descriptors and outcomes, which were then cross-checked by a third reviewer. We prioritized meta-analyses covering TSPO PET imaging, postmortem microglial data, and CSF kynurenine markers. Important original studies published after the most recent meta-analyses were also included to ensure up-to-date coverage.

**e) Targeted Unsystematic Search for the Narrative Sections of the Main Article**

For the narrative portions of the review – which introduce the topic, contextualize the quantitative findings, and discuss mechanisms, methods, and clinical translation – we conducted an iterative, topic-scoped literature search. This narrative search was not intended to be exhaustive; rather, it prioritized foundational and state-of-the-art publications relevant to the review’s aims. We queried PubMed and Google Scholar, performed backward and forward citation chaining of key papers and guidelines, and consulted authoritative narrative reviews and position statements. Representative keyword families included: microglia; glia; histopathology; postmortem; morphology; TMEM119; Iba1; HLA-DR; cerebrospinal fluid; inflammation; cytokines; chemokines; biomarkers; kynurenine; neuroplasticity; neurogenesis; synaptic pruning; extracellular vesicles/microvesicles; miRNA/microRNA; meta-analysis; positron emission tomography; radioligands; TSPO; brain imaging; precision medicine/psychiatry; microglial inhibition/modulation; anti-inflammatory drugs; and cyclooxygenase.

## 2. Meta-Analytic Extraction and Effect Size Interpretation

## **a) Data Extraction for Quantitative Synthesis**

For the studies and meta-analyses contributing to the quantitative synthesis, we extracted standardized mean differences (SMDs; Hedges’ g) and their 95% confidence intervals (CI) from published reports comparing MDD or SCZ with healthy controls. For meta-analyses, we additionally recorded heterogeneity metrics (I²) and precision metrics (relative CI width, rCIW), and we approximated τ² (between-study variance on the SMD² scale) and the typical within-study variance (*v*) when these were not provided. This extraction covered four key domains of evidence: (a) microglial PET imaging studies, (b) postmortem microglial marker studies, (c) kynurenine pathway metabolites in CSF, and (d) kynurenine pathway markers in postmortem brain tissue. For TSPO PET studies, we noted details such as the radioligand generation used (e.g., [11C]PK11195 vs. second-generation tracers), the outcome quantification method (e.g., total volume of distribution [VT] vs. reference tissue binding measures like BPND, DVR, or SUVr), and any stratification by TSPO binding-affinity genotype (high-/mixed-/low-affinity binders) if reported. For postmortem studies, we recorded the microglial markers assessed (e.g., Iba1, HLA-DR, TMEM119) and the morphometric or densitometric outcomes. For CSF studies, we extracted concentrations of kynurenine pathway analytes (e.g., kynurenine [KYN], kynurenic acid [KYNA], quinolinic acid [QUIN]) and related enzymatic markers (e.g., indoleamine 2,3-dioxygenase [IDO], tryptophan 2,3-dioxygenase [TDO], kynurenine 3-monooxygenase [KMO]) when available.

## **b) Cross-Referencing Study Coverage Across TSPO PET Meta-Analyses**

## We cross-referenced the sets of original TSPO PET studies included in prior meta-analyses with those in the recent meta-analysis by De Picker et al. [148]. This confirmed a largely overlapping coverage, as summarized here:

**Enache et al. [165]:** All six MDD PET studies reported in Enache et al. (Richards et al. [74], Holmes et al. [78], Setiawan et al. [72], Su et al. [88], Li et al. [76], Hannestad et al. [174]) were included in De Picker et al. [148].

**Gritti et al. [175]:** Eight of nine studies were included in De Picker et al.. The only missing study (Attwells et al. [176]) was a pharmacological intervention trial (celecoxib add-on) rather than a baseline diagnostic comparison, explaining its exclusion.

**Marques et al. [164]:** All twelve SCZ PET studies listed by Marques et al. were also present in De Picker’s dataset [148], including foundational studies such as van Berckel et al. [123], Doorduin et al. [121], and Coughlin et al. [91].

**Plavén-Sigray et al. [157]:** De Picker et al. [148] included all seven TSPO PET studies from the Plavén-Sigray individual participant data (IPD) meta-analysis on psychosis, including Collste et al. [177], Hafizi et al. [83], and Laurikainen et al. [178].

**Eggerstorfer et al. [150]:** All primary PET studies relevant to SCZ or MDD cited by Eggerstorfer were included in the De Picker synthesis [148].

In conclusion, **De Picker et al. [148] encompassed all the relevant original TSPO PET studies** reported in these earlier meta-analyses. The only exception was a single pharmacological study that was not relevant for baseline diagnostic contrasts [176]. Additionally, they discriminated between total volume‑of‑distribution (**VT)**–based models vs. reference tissue (**RT**)-based indices such as BPND/DVR/SUVr. Together, these observations confirmed that no key TSPO PET studies were missing from our review, and they justified our reliance on the De Picker dataset as a unified, non-redundant foundation for analysis.

According to the Methods section of De Picker et al. [148], their illness-category label ‘MOOD’ refers to MDD only. BD is listed separately as ‘OTHER’ and was not included as a separate diagnostic category in their meta-analysis. During data extraction, we noted a discrepancy in the MDD cingulate cortex meta-analysis values reported by De Picker et al. [148]. In Supplementary Table 4 of their paper, the cingulate cortex had an SMD of 0.821 with a 95% CI of 0.036–0.450, which is mathematically inconsistent with the given standard error (SE = 0.189). After correspondence with the authors (L. De Picker and J. Ottoy, 1 Oct 2025), the discrepancy was traced to a copy-and-paste error that misaligned the confidence interval columns during table revision. A re-analysis using the Comprehensive Meta-Analysis software confirmed the correct values: SMD = 0.821 (variance = 0.036; SE = 0.189), with a 95% CI of 0.450–1.192 and a 95% prediction interval of –0.123 to 1.765. All references to this region of interest in our review use these corrected values (**Supplementary Table 1**).

**MDD – Published versus corrected cingulate cortex data**

| Measure | Published value (Suppl. Table 4) [148] | Corrected value (CMA re‑analysis after email correspondence) | Note |
| --- | --- | --- | --- |
| k (comparisons) | 6 | 6 | Unchanged. |
| SMD | 0.821 | 0.821 | Unchanged. |
| SE | 0.189 | 0.189 | Unchanged. |
| Variance | — (not shown) | 0.036 | Value mistakenly placed in the “Lower 95% CI” column. |
| Lower 95% CI | 0.036 | 0.450 | Published lower bound was the variance; corrected lower CI = 0.450. |
| Upper 95% CI | 0.450 | 1.192 | Published upper bound was the true lower CI; corrected upper CI = 1.192. |
| Z | 1.192 | 4.34 | Published “Z” cell contained the true upper CI; correct Z = SMD/SE ≈ 4.34. |
| p-value | < 0.001 | < 0.001 | Unchanged (consistent with corrected Z). |
| I² | 37.6% | 37.6% | Unchanged. |

In addition, because De Picker et al. [148] did not report a meta-analytic summary for the prefrontal cortex (PFC), we gathered data from the relevant original TSPO PET studies [52, 72, 78, 83, 90, 99, 179] and calculated pooled effect size estimates for the PFC in each disorder. These supplemental analyses for PFC in MDD and SCZ are provided in **Supplementary Table 1**.

**MDD – Supplementation of prefrontal cortex data**

| Study | Radioligand | Region | Outcome Measure | MDD Mean | MDD SD | Control Mean | Control SD | Sample Size  MDD / Controls |
| --- | --- | --- | --- | --- | --- | --- | --- | --- |
| Holmes et al. [78] | [11C](R)-PK11195 | PFC | RT-based | 0.116 | 0.085 | 0.09 | 0.048 | 14/13 |
| Schubert et al. [179] | [11C]PK11195 | PFC | RT-based | 1.204 | 0.035 | 1.194 | 0.04 | 51/25 |
| Setiawan et al. [72] | [18F]FEPPA | PFC | VT-based | 11.65 | 2.9 | 10 | 2.8 | 50/30 |

**SCZ – Supplementation of prefrontal cortex data**

| Study | Radioligand | Region | Outcome Measure | SCZ Mean | SCZ SD | Control Mean | Control SD | Sample Size  SCZ / Controls |
| --- | --- | --- | --- | --- | --- | --- | --- | --- |
| Conen et al. [52] | [11C]PK11195 | PFC | RT-based | 0.061 | 0.076 | 0.071 | 0.05 | 41/21 |
| Hafizi et al. [83] | [18F]FEPPA | PFC | VT-based | 9.64 | 0.64 | 10.26 | 0.64 | 19/20 |
| Holmes et al. [90] | [11C]PK11195 | PFC | RT-based | 0.101 | 0.081 | 0.065 | 0.051 | 16/16 |
| Kenk et al. [99] | [18F]FEPPA | PFC | VT-based | 9.83 | 0.76 | 9.51 | 0.57 | 16/27 |

## **c) Interpretation of Effect Sizes**

Effect sizes were classified as [180]:

- Large: > 0.7

- Moderate: 0.3–0.69

- Small: < 0.3

## **d) Cross-Disorder Comparison of Effect Sizes**

We extracted the pooled random-effects SMDs and their standard errors (SE) for MDD and SCZ from each region-of-interest (ROI) reported by De Picker et al. [148]. We supplemented these with the pooled estimates we calculated for the PFC, as described above. For each ROI with estimates available for both diagnoses, we then compared the MDD and SCZ effects using an independent two-sample Z-test [181]:

Z = (g_MDD – g_SCZ) / √(SE_MDD² + SE_SCZ²)

**g** = Effect size

**SE** = Standard error of g

A positive Z indicates larger case–control effects in MDD than in SCZ. Given that the quantification method (VT-based vs. RT-based) is a known moderator in TSPO PET studies, we repeated the MDD-versus-SCZ comparisons within method-matched subgroups whenever possible (i.e. comparing VT-based outcomes between disorders, and separately comparing RT-based outcomes). All tests were two-sided, and in light of the exploratory objectives of this review, p-values were not adjusted for multiple comparisons.

## 3. Precision and Variability Metrics

*Rationale for the chosen approach:* Our primary aim was to determine which disorder shows greater true biological variability in microglial PET findings using only published summary statistics. A direct meta-analytic comparison of variance (e.g., via log-transformed variance ratios or coefficient of variation ratios) was not feasible, as individual study standard deviations and sample sizes were unavailable. Furthermore, comparing pooled meta-analytic SEs via an F-test would be inappropriate because a meta-analytic SE reflects both study precision and the number of studies, rather than solely underlying heterogeneity. We therefore focused on established meta-analytic heterogeneity metrics and mitigated methodological confounds by stratifying analyses into method-matched (VT-only) subsets versus overall mixed-method pools.

**a) Between‑Study absolute Variance (τ²) – Primary Heterogeneity Metric**

For each diagnosis and ROI, we approximated τ² from the reported I², number of studies (*k*), and the pooled SMD standard error, using the standard I²–τ² conversion under an equal-precision assumption:

Here 𝑣 denotes the typical within‑study sampling variance for a study in that meta‑analysis. We flagged these τ² values as approximate, with particular caution when *k* was small (≤3) or I² was near zero (if *k*=1, heterogeneity was not estimable and τ² was not applicable). We emphasized τ² as the primary heterogeneity metric because it reflects the absolute between-study dispersion on the effect-size scale, directly aligning with our objective of assessing potential biological heterogeneity.

**b) Relative Heterogeneity (I²) – Complementary Descriptor**

We also reported I², which represents the percentage of observed variance attributable to between-study heterogeneity rather than sampling error. We interpreted I² using conventional thresholds: low (<40%), moderate (40–75%), and high (>75%). For analyses with very few studies (*k*≤3) or single-study meta-analyses (*k*=1), I² estimates were treated with caution or considered not applicable.

**c) Within‑Study Variance (v) – Secondary Precision Indicator**

In parallel, we approximated the typical within-study variance (*v*) for each meta-analysis, consistent with the equal-precision assumption described above.

*v* served as an indicator of average study precision (with a higher *v* implying lower precision). We considered these *v* estimates to be approximate. Because *v* was computed within method-homogeneous subsets (separately for VT-only studies and for RT-only studies), it should be less influenced by between-study methodological differences; however, *v* remains sensitive to sample size and measurement noise.

**d) Internal Precision (relative CI Width; rCIW)**

As a measure of the precision of the pooled effect size estimates, we calculated the relative confidence interval width, rCIW=(CI_upper_−CI_lower_)/∣effect size∣. We qualitatively categorized the rCIW as narrow (≤1.0), moderate (1.0–2.0), or wide (>2.0) to aid interpretation of estimate accuracy.

**e) Two‑Tier, Method‑Matched Design**

To minimize methodological confounding (e.g., differences in PET outcome measures or radioligand generation), we evaluated heterogeneity at two levels: (1) using only VT-based studies for each disorder and ROI (a method-matched subset), and (2) using the overall pool of studies (mixed VT/RT methods) for completeness. We treated any observed cross-diagnostic differences in τ², I², or *v* as descriptive; no direct statistical tests of heterogeneity between MDD and SCZ were performed, given the lack of study-level data to support such comparisons.

**f) Assumptions, Advantages and Limitations**

*Assumptions:* The τ² and v calculations assume relatively similar within-study variances across studies in each meta-analysis. Moreover, heterogeneity estimates (I², τ²) become unstable when the number of studies is small, and are not defined when *k*=1.

*Advantages:* Our approach leverages standard heterogeneity constructs (τ², I²) and uses method-matched (VT- or RT-based) subsets to reduce confounds from varying PET quantification methods; it is also feasible given only summary published data.

*Limitations:* The cross-diagnostic heterogeneity comparisons are descriptive rather than inferential. I² values depend on typical study precision and the number of studies (*k*). Additionally, residual methodological differences (e.g., ligand generation, modeling choices, sample characteristics) may still influence heterogeneity even within VT-matched subsets.

**Supplementary Tables**

**Supplementary Table 1:** TSPO PET Results in MDD and SCZ.

The table reports meta-analytic results from TSPO PET studies in MDD and SCZ across key brain regions, along with direct statistical comparisons of effect sizes and between-study heterogeneity between the two diagnostic groups. Where available, both overall effects and subgroup analyses based on quantification method (volume‑of‑distribution (VT)–based models vs. reference tissue (RT)-based indices such as BPND/DVR/SUVr) are shown. Data were extracted from De Picker et al. [148], including corrected cingulate cortex data for MDD vs. controls. Additionally, we conducted our own meta-analytic evaluation of prefrontal cortex data [52, 72, 78, 83, 90, 99, 179]. Significant p-values are highlighted in bold.

| **Brain region** | **MDD vs. Controls** | | | | | | | **SCZ vs. Controls** | | | | | | | **MDD- vs. SCZ-studies** | |
| --- | --- | --- | --- | --- | --- | --- | --- | --- | --- | --- | --- | --- | --- | --- | --- | --- |
|  | k studies | SMD | p-value | I² | τ² | *v* | rCIW | k studies | SMD | p-value | I² | τ² | *v* | rCIW | Z | Z-test  p-value |
| **Whole-brain cortical grey matter (overall)*** | 5 | 0.525 | 0.062 | 72.2 | 0.285 | 0.110 | 2.10 | 20 | -0.087 | 0.561 | 66.4 | 0.299 | 0.151 | 6.74 | 1.921 | 0.055 |
| Whole-brain cortical grey matter (VT-based)* | 5 | 0.525 | 0.062 | 72.2 | 0.285 | 0.110 | 2.10 | 9 | -0.506 | **0.016** | 66.5 | 0.264 | 0.133 | 1.62 | 2.939 | **0.003** |
| Whole-brain cortical grey matter (RT-based)* | n.a. | n.a. | n.a. | n.a. | n.a. | n.a. | n.a. | 7 | 0.096 | 0.520 | 2.0 | 0.003 | 0.150 | 6.05 | n.a. | n.a. |
| **Thalamus (overall)*** | 4 | 0.566 | 0.127 | 75.8 | 0.417 | 0.133 | 2.57 | 9 | -0.242 | 0.110 | 19.8 | 0.041 | 0.165 | 2.45 | 2.017 | **0.044** |
| Thalamus (VT-based)* | 4 | 0.566 | 0.127 | 75.8 | 0.417 | 0.133 | 2.57 | 2 | -0.181 | 0.790 | 83.0 | 0.761 | 0.156 | 14.66 | 0.968 | 0.333 |
| Thalamus (RT-based)* | n.a. | n.a. | n.a. | n.a. | n.a. | n.a. | n.a. | 6 | -0.270 | 0.094 | 0.0 | 0.000 | 0.156 | 2.34 | n.a. | n.a. |
| **Hippocampus (overall)*** | 4 | 0.691 | **<0.001** | 18.4 | 0.021 | 0.092 | 0.95 | 8 | -0.263 | 0.317 | 74.3 | 0.411 | 0.142 | 3.91 | 3.057 | **0.002** |
| **Cingulate cortex (overall) ^#^** | 6 | 0.821 | **<0.001** | 37.6 | 0.081 | 0.134 | 0.90 | 10 | -0.191 | 0.267 | 48.0 | 0.142 | 0.154 | 3.53 | 3.960 | **<0.001** |
| **Prefrontal cortex (overall) ^#^** | 3 | 0.416 | **0.007** | 0.0 | 0.000 | 0.073 | 1.47 | 4 | -0.031 | 0.916 | 70.0 | 0.238 | 0.102 | 37.25 | 1.351 | 0.177 |
| Prefrontal cortex (VT-based)^#^ | 1 | 0.571 | **0.015** | n.a. | n.a. | 0.055 | 1.62 | 2 | -0.231 | 0.653 | 79.4 | 0.417 | 0.108 | 8.72 | 1.420 | 0.156 |
| Prefrontal cortex (RT-based)^#^ | 2 | 0.296 | 0.153 | 0.0 | 0.000 | 0.086 | 2.75 | 2 | 0.094 | 0.664 | 0.4 | 0.000 | 0.093 | 9.03 | 0.676 | 0.499 |

**Column Definitions:**

- **Brain region**: Region of interest examined.
- **k studies**: Number of studies included in the meta-analysis.
- **SMD**: Standardized mean difference in TSPO binding between patients and healthy controls (positive = increase in patients).
- **p-value**: Statistical significance of the SMD.
- **I²**: Between-study heterogeneity (in %); interpreted as low (< 40%), moderate (40–75%), or high (> 75%). Not applicable when k=1; interpret cautiously when k≤3.
- **τ²:** approximated absolute between-study variance of true effects (see Supplementary Methods).
- ***v*:** approximated typical within-study sampling variance (see Supplementary Methods); higher v = lower precision).
- **rCIW**: Relative width of the 95% confidence interval (CI); calculated as (CI_upper_ – CI_lower_)/|SMD|.
  - Interpretation: narrow (≤ 1.0), moderate (1.0–2.0), wide (> 2.0).
- **Z and Z-test p-value**: Z-value and p-value from Z-test comparing MDD and SCZ SMDs for the same brain region. Positive Z means: MDD > SCZ.

**Sources:**

- *: pooled estimates from De Picker et al. [148];
- #: pooled estimates from our own meta-analytic evaluation for prefrontal cortex and cingulate cortex data [52, 72, 78, 83, 90, 99, 179].

**Supplementary Table 2:** Summary and vote counting overview of microglial density in MDD postmortem studies.

| **Analyzed Brain Region(s)** | **Markers Used** | **Sample Size (MDD / Controls)** | **Findings (MDD vs. Controls)** | **Reported Increase in Microglial Activation Markers** | **No Change / Decrease in Microglial Activation Markers** |
| --- | --- | --- | --- | --- | --- |
| Dorsal raphe nucleus | HLA-DR | 15/22 | Brisch et al. (2017) [86]: ↔ No diagnosis-related difference in HLA-DR+ microglial density; ↓ Decrease only in non-suicidal patients | 0 | 1 (overall no difference, ↓ only in non-suicidal patients) |
| DLPFC, hippocampus, ACC, Mediodorsal thalamus | HLA-DR | 9/10 | Steiner et al. (2008) [124]: ↔ No diagnosis-related difference in HLA-DR+ microglial density; ↑ Increase only in suicidal patients | 0 | 1 (↑ only suicidal patients) |
| VLPFC | IBA1 | 45/36 | Clark et al. (2016) [92]: ↑ Increased density of amoeboid (activated) IBA1+ microglia in MDD | 1 | 0 |
| ACC | IBA1, CD45 | 24/17 | Torres-Platas et al. (2014) [103]: ↔ No overall difference in microglial density; ↑ Increased primed/resting ratio | 1 (ratio ↑) | 1 (density same) |
| DLPFC | Calprotectin | 15/15 | Foster et al. (2006) [182]: ↔ No difference in calprotectin expressing microglia | 0 | 1 |
| BA 24; BA 46 | CD11b | 10/30 | Dean et al. (2013) [183]: ↔ No difference in CD11b | 0 | 1 |
| Amygdala | HLA; Nissl | 8/10 | Hamidi et al. (2004) [133]: ↔ No difference in HLA and Nissl-stained microglia | 0 | 1 |
| Medial frontal gyrus; superior temporal gyrus; thalamus; subventricular zone | CD11b; CD45; HLA-DR; CD68; P2Y12; TMEM119; CCR5 | 6/5 | Böttcher et al. (2020) [64]: ↔ No induction of classical microglial activation markers (no upregulation of CD11b, CD45, HLA-DR or CD68); ↑ increased levels of homeostatic markers (P2Y12, TMEM119, CCR5) in MDD | 0 | 1 |
| Medial frontal gyrus; superior temporal gyrus; thalamus; subventricular zone | CX3CR1; TMEM119; CD163; CD14; HLA-DRA; IL-6; IL-1β | 19/25 | Snijders et al. (2021) [8]: ↑ Increased expression of homeostatic microglial genes (CX3CR1, TMEM119 mRNA); ↓ decreased CD163 mRNA and CD14 protein; ↔ no change in classical activation markers (HLA-DRA, IL-6, IL-1β); overall non-inflammatory microglial profile in MDD | 0 | 1 |

**Note:** Two further studies were identified that examined microglial markers in depressive disorders. However, these could not be included because a) Loef et al. [184] lacked separate analysis for MDD versus BD, and b) Wesseling et al. [102] measured coronin 1A by mass spectrometry in bulk tissue, a method that cannot distinguish microglial from non-microglial sources (e.g., T-cells, macrophages, neutrophils, and astrocytes [185, 186]).

**Definitions:**

**Reported Increase in Microglial Activation Markers:** Any statistically significant increase (↑) in microglial cell density or gene/protein expression of activation markers in MDD compared to controls.
**No Change or Decrease in Microglial Activation Markers:** Includes studies reporting no statistically significant difference (↔) or a significant decrease (↓) in microglial-related measures in MDD compared with controls, as well as findings restricted to suicidal subgroups without specific without an overall diagnosis-level effect.

**Abbreviations:** **ACC,** Anterior Cingulate Cortex; **DLPFC,** Dorsolateral Prefrontal Cortex; **VLPFC,** Ventrolateral Prefrontal Cortex; **BA**, Brodmann's area.

**Supplementary Table 3:** Summary and vote counting overview of microglial density in BD postmortem studies.

| **Analyzed Brain Region(s)** | **Markers Used** | **Sample Size (BD / Controls)** | **Findings (BD vs. Controls)** | **Reported Increase in Microglial Activation Markers** | **No Change / Decrease in Microglial Activation Markers** |
| --- | --- | --- | --- | --- | --- |
| Dorsal raphe nucleus | HLA-DR | 12/22 | Brisch et al. (2017) [86]: ↔ No diagnosis-related difference in HLA-DR+ microglial density; ↓ Decrease only in non-suicidal patients | 0 | 1 (overall no difference, ↓ only in non-suicidal affective disorder) |
| DLPFC, hippocampus, ACC, Mediodorsal thalamus | HLA-DR | 5/10 | Steiner et al. (2008) [124]: ↔ No diagnosis-related difference in HLA-DR+ microglial density; ↑ Increase only in suicidal affective disorder | 0 | 1 (↑ only suicidal affective disorder) |
| DLPFC | Calprotectin | 15/15 | Foster et al. (2006) [182]: ↔ No difference in calprotectin expressing microglia | 0 | 1 |
| BA 24; BA 46 | CD11b | 10/30 | Dean et al. (2013) [183]: ↔ No difference in CD11b | 0 | 1 |
| Amygdala | HLA; Nissl | 9/10 | Hamidi et al. (2004) [133]: ↔No difference in HLA and Nissl-stained microglia | 0 | 1 |
| Prefrontal white matter | IBA1 | 20/20 | Hercher et al. (2014) [105]: ↔No difference in IBA1+ microglia | 0 | 1 |
| DLPFC | CX3CR1 | 34/35 | Hill et al. (2021) [49]: ↔No difference in CX3CR1 protein and mRNA expression | 0 | 1 |
| Hippocampus | P2RY12; MHC II | 15/12 | Naggan et al. (2023) [187]: ↔ No overall difference in microglial density; ↑ Increase only in suicidal BD | 0 | 1 (↑ only suicidal BD) |
| aMCC | IBA1 | 13/17 | Petrasch-Parwez et al. (2020) [188]: ↔ No overall difference in IBA1+ microglial density; density was significantly lateralized to the right aMCC, which was not observed in controls ↓ Decrease only in non-suicidal BD | 0 | 1 (↓ only in non-suicidal BD) |
| Frontal cortex | HLA-DR  CD11b | 10/10 | Rao et al. (2010) [189]: ↑ HLA-DR+ microglia (qualitative evaluation), higher CD11b protein and mRNA levels | 1 | 0 |
| Cingulate Cortex; PFC | CD11b; CD68; IBA1 | 35/35 | Seredenina et al. (2017) [190]: ↓ decreased CD11b+ and CD68+ microglia, ↔ unchanged IBA1+ microglia | 0 | 1 |
| Medial frontal gyrus; superior temporal gyrus; thalamus | IBA1 P2RY12; TMEM119; CD68; CX3CR1; CD11b, HLA-DR | 16/12 | Sneeboer et al. (2019) [191]: ↔ no significant difference in IBA1+ microglia, no significant difference in assessed microglial markers | 0 | 1 |
| DLPFC; ACC | CD68; CX3CR1; HLA-DR; IBA1; P2RY12; TREM2; TSPO | 30/34 | Zhang et al. (2020) [192]: ↓ CD68, TREM2 mRNA levels in DLPFC, ↓ P2RY12 in non-suicidal patients in DLPFC, ↔ no change in other microglial mRNA expression in the ACC | 0 | 1 |
| Cingulate white matter | IBA1 | 15/21 | Connor et al. (2009) [193]: ↔ no difference in IBA1+ immunoreactivity | 0 | 1 |

**Note:** Three further studies were identified that examined microglial markers in bipolar disorder. However, these could not be included because a) Loef et al. [184] lacked separate analysis for MDD versus BD, b) Wesseling et al. [102] measured coronin 1A by mass spectrometry in bulk tissue, a method that cannot distinguish microglial from non-microglial sources (e.g., T-cells, macrophages, neutrophils, and astrocytes [185, 186]), and c) Zheng et al. [19] primarily examined CMV-associated microglial activation without providing diagnosis-specific statistical comparisons.

**Definitions:**

**Reported Increase in Microglial Activation Markers:** Any statistically significant increase (↑) in microglial cell density or gene/protein expression of activation markers in BD compared to controls.
**No Change or Decrease in Microglial Activation Markers:** Includes studies reporting no statistically significant difference (↔) or a significant decrease (↓) in microglial-related measures in BD vs. controls, as well as findings restricted to suicidal subgroups without an overall diagnosis-level effect.

**Abbreviations:** **ACC,** Anterior Cingulate Cortex; **PFC**, Prefrontal Cortex; **DLPFC,** Dorsolateral Prefrontal Cortex; **VLPFC,** Ventrolateral Prefrontal Cortex; **BA**, Brodmann's area; **aMCC**, Anterior midcingulate cortex.

**Supplementary Table 4:** Summary of meta-analyses on postmortem microglial alterations in SCZ compared to controls [155, 169].

| **Author of Meta-Analysis (Year)** | **Analyzed Brain Regions** | **Markers / Technique Used** | **Parameter** | **Brain Regional Focus of Significant Results** | **Significant Diagnosis-related Differences between SCZ and Controls** |
| --- | --- | --- | --- | --- | --- |
| **Van Kesteren et al. (2017) [169]** | PFC, ACC, Temporal Cortex / White Matter, Hippocampus | **IHC:** HLA-DR, CD68, Iba1 | Microglial Density | **Temporal Cortex** | **Overall:** **↑ Microglial density** (SMD = 0.69, 95% CI:0.24–1.14, rCIW = 1.30, p = 0.0028), high heterogeneity (I² = 68.6%)  **Temporal cortex:** strongest ↑ in microglial density (p = 0.033; but did not provide SMD and CI) |
|  |  | **PCR:** IL-1β, IL-6, TNF-α, COX-2, IL-10, TGF-β | Pro- and Anti-Inflammatory Gene Expression  *(not specific for microglia!)* | **PFC and hippocampus most frequently studied** here.  Other brain regions (e.g., white matter, temporal cortex) were underrepresented, meaning region-specific effects remained unclear. | **Overall:** **↑ Proinflammatory gene expression** (SMD = 0.37, 95% CI: 0.11–0.62, rCIW = 1.38, p = 0.0052), I² n.a.; ↑Increased expression of IL-1β, IL-6, TNF-α, COX-2; ↔no significant changes in anti-inflammatory markers (IL-10, TGF-β). |
| **Snijders et al. (2021) [155]** | PFC, ACC and MCC, Temporal Cortex, Hippocampus | **IHC:** HLA-DR, Iba1, CD68, NOX2  (original study part of the paper: TMEM119) | Microglial Density | **Temporal Cortex** (before outlier correction) | **Overall:** **↔No significant increase in microglial density** (g = 0.144, 95% CI: 0.102–0.390, rCIW = 2.0, p = 0.250), low heterogeneity (I² = 18.9%).  **Temporal cortex:** ↑ Microglial density (g = 1.256, 95% CI: 0.55–1.96, rCIW = 1.12, p < 0.001) disappeared after *removing outlier studies*.  (original study part of the paper: ↓ co-localization of Iba1 and TMEM119 revealed a lower percentage of TMEM119+/Iba1+ microglia in SCZ) |
|  |  | **PCR:** Iba1 (=AIF1), CD68, CSF1R, HLA-DRA, HLA-DRB, ITGAM, NOX2, TGFB1  (original study part of the paper: TMEM119) | Microglial Gene Expression | **PFC, ACC and MCC, and hippocampus most frequently studied** here. | **↓ Microglial gene expression** (g = -0.417, 95% CI: -0.417 to -0.546, rCIW = 0.31, p < 0.0001), low heterogeneity (I² = 26.7%); downregulation of homeostatic microglial markers TMEM119, CX3CR1, CSF1R, IRF8, OLR1. ↔ **No increase in classical activation markers** (HLA-DR, CD68, NOX2), suggesting absence of neuroinflammatory response.  (original study part of the paper: ↓ TMEM119 gene expression) |

**Abbreviations:**
**ACC**, Anterior Cingulate Cortex; **AIF1**, Allograft Inflammatory Factor 1 (synonym for Iba1); **CD68**, Cluster of Differentiation 68, a marker for activated microglia/macrophages; **CI**, Confidence Interval, indicating statistical certainty; **COX-2**, Cyclooxygenase-2, an enzyme involved in inflammation; **CSF1R**, Colony Stimulating Factor 1 Receptor, involved in microglial survival; **CX3CR1**, Fractalkine Receptor, involved in neuron-microglia communication; **g**, Hedges g, effect size measure, similar to Cohen’s d but adjusted for small samples; **HLA-DRA**, Major Histocompatibility Complex, Class II, DR Alpha; **HLA-DRB**, Major Histocompatibility Complex, Class II, DR Beta; **HLA-DR**, Human Leukocyte Antigen – DR isotype, a microglial activation marker; **Iba1**, Ionized Calcium Binding Adapter Molecule 1, a marker for microglia; **IHC**, Immunohistochemistry; **IL-10**, Interleukin 10, an anti-inflammatory cytokine; **IL-1β**, Interleukin 1 Beta, a pro-inflammatory cytokine; **IL-6**, Interleukin 6, a cytokine involved in inflammation; **IRF8**, Interferon Regulatory Factor 8, involved in microglial development; **I²**, I-squared, a measure of heterogeneity in meta-analyses; **ITGAM**, Integrin Subunit Alpha M, also known as CD11b, a macrophage/microglial marker; **MCC**, Midcingulate Cortex; **NOX2**, NADPH Oxidase 2, an enzyme involved in reactive oxygen species production; **OLR1**, Oxidized Low-Density Lipoprotein Receptor 1, associated with microglial function; **PCR**, Polymerase Chain Reaction; **PFC**, Prefrontal Cortex; **QUIN**, Quinolinic Acid, a neurotoxic metabolite associated with microglial activation; **rCIW,** relative Confidence Interval Width; **SMD**, Standardized Mean Difference, a statistical measure used in meta-analyses; **TGFB1**, Transforming Growth Factor Beta 1, involved in immune regulation; **TGF-β**, Transforming Growth Factor Beta, a regulatory cytokine; **TMEM119**, Transmembrane Protein 119, a marker specific to homeostatic microglia; **TNF-α**, Tumor Necrosis Factor Alpha, a pro-inflammatory cytokine.

**Supplementary Table 5:** Summary of meta-analyses on CSF kynurenine metabolites in MDD, BD and SCZ.

| **Author of Meta-Analysis (Year)** | **Studies (k)** | **Sample Size (Patients/Controls)** | **Tested Kynurenine Metabolites** | **Outcome** | **Significant Diagnosis-related Differences between Patients and Controls** |
| --- | --- | --- | --- | --- | --- |
| **Inam et al. (2023) [147]** | 11 | 217/254 | QUIN, KYNA, KYN, TRP  (3-HK, 3-HAA, PIC not systematically analyzed) | SMD | **MDD:** Trend towards **QUIN increase** (SMD =  2.66, CI: -1.04-6.35, rCIW = 2.78, p = 0.1594, I^2^= 98%), but small sample size (k=2, n=136) prevents firm conclusions. |
|  | 8 | 238/240 | KYNA, KYN, TRP  (QUIN, 3-HK, 3-HAA, PIC not systematically analyzed) | SMD | **SCZ:** ↑ KYNA (SMD = 2.64, 95% CI: 1.16-4.13, rCIW = 1.13, p = 0.0005, I^2^= 96%, k = 6, n=384). |
|  | 6 | 246/340 | KYNA, TRP  (KYN, QUIN not systematically analyzed) | SMD | **BD:** Trend towards KYNA increase (SMD =1.40, CI: -0.28-3.09, rCIW = 2.41, p = 0.1032, I2= 98%), but small sample size (k=2, n=377) prevents firm conclusions. |
| **Rømer et al. (2023) [146]** | 6 | 282/303 | KYNA, KYN  (QUIN, 3-HK, 3-HAA, TRP, PIC not systematically analyzed) | SMD | **SCZ:** ↑ KYNA (SMD = 1.58, 95% CI: 0.34-2.81, rCIW = 1.56, p = 0.013, I^2^= 96%, k = 6, n=549). ↑ KYN (SMD = 1.00, 95% CI: 0.64-1.36, rCIW = 0.72, p < 0.001, I^2^= 17%, k=3, n=113). |

**Abbreviations:**
**3-HAA**, 3-Hydroxyanthranilic Acid; **3-HK**, 3-Hydroxykynurenine; **BD**, Bipolar Disorder; **CI**, Confidence Interval; **CSF**, Cerebrospinal Fluid; **I²,** Inconsistency Index; **KYN**, Kynurenine; **KYNA**, Kynurenic Acid; **MDD**, Major Depressive Disorder; **PIC**, Picolinic Acid; **QUIN**, Quinolinic Acid; **rCIW,** relative Confidence Interval Width; **SCZ**, Schizophrenia; **SMD**, Standardized Mean Difference; **TRP**, Tryptophan.

**Supplementary Table 6:** Summary of kynurenine metabolites and related enzymes in MDD postmortem studies.

| **Analyzed Brain Region(s)** | **Analyzed Markers / Enzymes** | **Sample Size (MDD/Controls)** | **Findings (MDD vs. Controls)** |
| --- | --- | --- | --- |
| aMCC, sACC, pACC | QUIN (IHC) | 7/10 post-hoc  (primary analysis: Depression (MDD+BD: n=12) vs. controls n=10) | Steiner et al. (2011) [116]: post-hoc MDD vs. controls ↑ QUIN+ microglia in sACC, aMCC, ↔ in pACC |
| Hippocampus | QUIN (IHC) | 6/10 post-hoc  (primary analysis: Depression (MDD+BD: n=12) vs. controls n=10) | Busse et al. (2015) [86]: post-hoc MDD vs. controls ↓ QUIN+ microglia in hippocampal right CA1 |
| ACC (unspecified subregion) | KYN, KYNA (HPLC)  & TDO2 (IHC and RT-PCR) | 14/14 | Miller et al. (2006) [128]: ↑ TDO2+ astrocytes in the white matter of the ACC |
| ACC (unspecified subregion) | TRP, 3-HAA, NAM (HPLC) | 14/14  (continuation of the Miller et al. (2006) [3] study, using the same samples) | Miller et al. (2008) [125]: ↔No significant differences |
| VLPFC (BA 45/47) | KYNA, 3-HK (HPLC);  TRP, KYN, QUIN (GC/MS)  & IDO1, IDO2, TDO2, KAT II, KMO (RT-PCR) | 45/36  (not MDD, but depressive disorder not otherwise specified) | Clark et al. (2016) [92]: ↓ QUIN, ↓ Kynurenine:TRP ratio & ↓ IDO1, ↓ IDO2, ↓ TDO2 |
| ACC (BA 24) | Trp, KYN, 3-HK, 3-HAA, XA, QUIN, KYNA, formic acid (LCMS)  & KMO, KAT I, KAT II, KYNU, HAAO, QPRT (RT-PCR) | 44/36 | Brown et al. (2024) [16]: ↓ KYNA only in females &  ↑ KAT II |

**Annotation:** Only **Brown et al. [16]** assessed **HAAO (3‑HAO)** transcript; none of the **MDD** postmortem studies assessed **3‑HAO/HAAO enzyme activity.**

**Abbreviations: 3-HAA**, 3-Hydroxyanthranilic Acid; **3-HK**, 3-Hydroxykynurenine; **ACC**, Anterior Cingulate Cortex; **aMCC**, Anterior Midcingulate Cortex; **BA**, Brodmann Area; **GC/MS**, Gas Chromatography–Mass Spectrometry; **HAAO**, 3-Hydroxyanthranilate 3,4-Dioxygenase (enzyme converting 3-HAA to QUIN); **HPLC**, High-Performance Liquid Chromatography; **IDO1**, Indoleamine 2,3-Dioxygenase 1 (mainly expressed in microglia); **IDO2**, Indoleamine 2,3-Dioxygenase 2 (less active isoform of IDO1); **IHC**, Immunohistochemistry; **KAT I / II**, Kynurenine Aminotransferase I / II (enzymes converting KYN to KYNA); **KMO**, Kynurenine 3-Monooxygenase (mainly expressed in microglia); **KYNA**, Kynurenic Acid (primarily astrocyte-derived); **KYNU**, Kynureninase (enzyme involved in downstream KYN metabolism); **LCMS**, Liquid Chromatography–Mass Spectrometry; **MDD**, Major Depressive Disorder; **NAM**, Nicotinamide; **pACC**, Pregenual Anterior Cingulate Cortex; **QPRT**, Quinolinate Phosphoribosyltransferase (enzyme converting QUIN to NAD⁺); **QUIN**, Quinolinic Acid (primarily produced by activated microglia); **RT-PCR**, Reverse Transcription Polymerase Chain Reaction; **sACC**, Subgenual Anterior Cingulate Cortex; **TDO2**, Tryptophan 2,3-Dioxygenase (liver- and brain-expressed enzyme producing KYN); **TRP**, Tryptophan; **VLPFC**, Ventrolateral Prefrontal Cortex; **XA**, Xanthurenic Acid.

**Supplementary Table 7:** Summary of kynurenine metabolites and related enzymes in BD postmortem studies.

| **Analyzed Brain Region(s)** | **Analyzed Markers / Enzymes** | **Sample Size (BD/Controls)** | **Findings (BD vs. Controls)** |
| --- | --- | --- | --- |
| aMCC, sACC, pACC | QUIN (IHC) | 5/10 post-hoc  (primary analysis: Depression (MDD+BD: n=12) vs. controls n=10) | Steiner et al. (2011) [116]: post-hoc BD vs. controls ↔ QUIN+ microglia (sACC, aMCC, pACC) |
| Hippocampus | QUIN (IHC) | 6/10 post-hoc | Busse et al. (2015) [86]: post-hoc BD vs. controls ↓ QUIN+ microglia in hippocampal right CA1 |
| ACC (unspecified subregion) | KYN, KYNA (HPLC)  & TDO2 (IHC and RT-PCR) | 14/14 | Miller et al. (2006) [128]: ↑ KYN and ↑ TDO2+ astrocytes in the ACC |
| ACC (unspecified subregion) | TRP, 3-HAA, NAM (HPLC) | 14/14  (continuation of the Miller et al. (2006) [3] study, using the same samples) | Miller et al. (2008) [125]: ↔No significant differences |
| PFC | KMO (microarray-based gene expression) | 34/35 | Lavebratt et al. (2014) [194]: ↓ KMO in BD patients with psychotic features |

**Annotation:** Unfortunately, none of these studies analyzed the expression of the enzyme 3-hydroxyanthranilate 3,4-dioxygenase (3-HAO), which is responsible for the microglial synthesis of QUIN.

**Abbreviations: 3-HAA**, 3-Hydroxyanthranilic Acid; **ACC**, Anterior Cingulate Cortex; **aMCC**, Anterior Midcingulate Cortex; **HPLC**, High-Performance Liquid Chromatography; **IHC**, Immunohistochemistry; **KMO**, Kynurenine 3-Monooxygenase (mainly expressed in microglia); **KYNA**, Kynurenic Acid (primarily astrocyte-derived); **BD**, Bipolar Disorder; **NAM**, Nicotinamide; **pACC**, Pregenual Anterior Cingulate Cortex; **PFC**, Prefrontal Cortex; **QUIN**, Quinolinic Acid (primarily produced by activated microglia); **RT-PCR**, Reverse Transcription Polymerase Chain Reaction; **sACC**, Subgenual Anterior Cingulate Cortex; **TDO2**, Tryptophan 2,3-Dioxygenase (liver- and brain-expressed enzyme producing KYN); **TRP**, Tryptophan.

**Supplementary Table 8:** Summary of kynurenine metabolites and related enzymes in SCZ postmortem studies.

| **Analyzed Brain Region(s)** | **Analyzed Markers / Enzymes** | **Sample Size (SCZ/Controls)** | **Findings (SCZ vs. Controls)** |
| --- | --- | --- | --- |
| Hippocampus | QUIN (IHC) | 13/12 | **Gos et al. (2014) [106]**: ↓ QUIN in CA1 |
| Anterior prefrontal cortex (BA10) | TDO2 (IHC, Western blot), TDO2, IDO, KMO, KYNU, KAT I (RT-PCR) | 15/17 | **Miller et al. (2004) [132]:** ↑ TDO2+ astroglial cell density in white matter, ↑ TDO2 mRNA |
| **ACC (unspecified subregion)** | KYN, KYNA (HPLC)  & TDO2 (IHC and RT-PCR) | 12/14 | **Miller et al. (2006) [128]:** ↑ KYN & ↑ TDO2 mRNA, ↑ TDO2+ astroglial cell density in the white matter of the ACC |
| **ACC (unspecified subregion)** | TRP, 3-HAA, NAM (HPLC) | 12/14 (continuation of the **Miller et al. (2006) [128]** study, using the same samples) | **Miller et al. (2008) [125]:** ↑ Trp, ↑ 3-HAA |
| DLPFC (BA9) & Cerebellum | KYNA, QUIN (ELISA) & IDO1, IDO2, TDO, KMO, KATII (Western blot) | 15/14 DLPFC, 15/13 Cerebellum | Afia et al. (2021) [195]: DLPFC: ↓ IDO2, ↑ TDO, ↑ KMO (p = 0.005), ↓ KATII  Cerebellum: ↔No significant differences between SCZ and controls |
| DLPFC (BA46, 9) - Gray & White Matter | TRP, KYN, KYNA, 3-HK, AA, 3-HAA, QUIN (HPLC) | 21/24 | Antenucci et al. (2024) [17]: ↑ KYN, ↑ 3-HK, ↑ AA, ↑ 3-HAA in both gray & white matter  ↑ KYNA & ↑ QUIN (p = 0.0027) only in white matter |
| DLPFC (BA46, 9) | TRP, KYN, KYNA, QUIN (HPLC)  & KAT I, KAT II, TDO, KMO (RT-PCR) | 71/72  (Cytokine measurements were used to divide the entire SCZ cohort into 28 high and 43 low inflammation cases) | Kindler et al. (2020) [60]: ↑ KYN/TRP ratio, ↑ KYNA in the high-inflammation SCZ subgroup  & ↑ TDO, ↑ KAT I and ↑ KAT II in the high-inflammation SCZ subgroup |
| **DLPFC (BA9) & Anterior prefrontal cortex** (BA10) | KYNA (HPLC)  & KMO, 3-HAO, QPRT, KAT II (Enzyme Activity Assays) | 15/15 | Sathyasaikumar et al. (2011) [118]: ↑ KYNA (BA9 and BA10)  & ↓ KMO (BA9 and BA10), ↓ 3-HAO (BA9, but BA10 not significant) |
| **DLPFC (BA9) & Anterior prefrontal cortex** (BA10) & Occipital lobe (BA19) | KYN, KYNA, 3-HK (HPLC) | 30/31 | Schwarcz et al. (2001) [136]: ↑ KYN (BA9 and BA19, but BA10 not significant), ↑ KYNA (BA9, but BA10 and BA19: not significant) |
| Frontal Eye Field (BA6) | KMO (RT-PCR, Enzyme Activity Assay) | 32/32 | Wonodi et al. (2011) [115]: ↓ KMO mRNA & enzyme activity |

**Annotation:** Only one schizophrenia study (Sathyasaikumar et al. [118]) assessed 3-HAO, a key enzyme in QUIN synthesis, and reported a reduction in BA9 (p = 0.01) but not BA10.

**Abbreviations: 3-HAA**, 3-Hydroxyanthranilic Acid; **3-HAO**, 3-Hydroxyanthranilate 3,4-Dioxygenase, enzyme converting 3-HAA to QUIN; **3-HK**, 3-Hydroxykynurenine; **AA**, Anthranilic Acid; **ACC**, Anterior Cingulate Cortex; **BA**, Brodmann Area; **CB**, Cerebellum; **DLPFC**, Dorsolateral Prefrontal Cortex; **ELISA**, Enzyme-Linked Immunosorbent Assay; **HPLC**, High-Performance Liquid Chromatography; **IDO1**, Indoleamine 2,3-Dioxygenase 1 (mainly expressed in microglia, also present in infiltrating macrophages and endothelial cells); **IDO2**, an isoform of IDO1 with similar but less active function; **IHC**, Immunohistochemistry; **KAT I/II**, Kynurenine Aminotransferases I-II, enzymes converting KYN to KYNA; **KMO**, Kynurenine Monooxygenase (predominantly expressed in microglia); **KYN**, Kynurenine; **KYNA**, Kynurenic Acid (mainly produced by astrocytes); **KYNU**, Kynureninase (expressed in both microglia and astrocytes); **NAM**, Nicotinamide; **QPRT**, Quinolinate Phosphoribosyltransferase, enzyme degrading QUIN to NAD+; **QUIN**, Quinolinic Acid (mainly produced by activated microglia); **RT-PCR**, Reverse Transcription Polymerase Chain Reaction; **SCZ**, Schizophrenia; **TDO2**, Tryptophan 2,3-Dioxygenase, enzyme involved in KYN production; **TRP**, Tryptophan.

**Supplementary Figure 1:** Flow diagram according to the PRISMA (Preferred Reporting Items for Systematic Reviews and Meta‑Analyses) criteria showing the process of study selection for human studies of microglia‑related markers in major depressive disorder (**MDD**) and schizophrenia (**SCZ**).

*Identification:* The database search yielded 4,942 records: Web of Science (n = 1,611), PubMed (n = 1,066), and Scopus (n = 2,265).

*Screening:* After removing duplicates (n = 1,925), 3,017 unique records remained. We then excluded narrative or off‑topic systematic reviews/meta‑analyses (n = 580), leaving 2,437 records for title/abstract and methods screening.

*Eligibility:* Of these, 2,264 records were excluded based on prespecified criteria, including: animal studies (n = 1,268), case reports (n = 18), no original data (n = 180), pediatric/adolescent psychiatry (n = 3), not SCZ or MDD (n = 543), mixed diagnoses (n = 19), in vitro/ex vivo only (n = 17), cross‑disorder comparisons without a control group (n = 22), no analysis of microglia/TSPO/kynurenines (n = 151), no quantification of these markers (n = 10), kynurenines reported only in blood/plasma (n = 28), and cytokine‑only studies (n = 5).

*Included:* 173 publications met eligibility and were included: 142 case‑control studies [1-142] and 31 systematic reviews/meta‑analyses [143-173].

(The Boolean search string and full inclusion/exclusion details are provided in the figure and Supplementary Methods.)

**Supplementary Figure 2:** Flow diagram according to the PRISMA (Preferred Reporting Items for Systematic Reviews and Meta‑Analyses) criteria showing the process of study selection for human studies of microglia‑related markers in bipolar disorder (**BD**).

*Identification:* The database search yielded 565 records: Web of Science (n = 221), PubMed (n = 101), and Scopus (n = 243).

*Screening:* After removing duplicates (n = 192), 373 unique records remained. We then excluded narrative or off topic systematic reviews/meta analyses (n = 149), leaving 224 records for title/abstract and methods screening.

*Eligibility:* Of these, 187 records were excluded based on prespecified criteria, including: animal studies (n = 40), case reports (n = 4), articles without original data (n = 29), pediatric/adolescent psychiatry (n = 1), studies not investigating BD (n = 38), patients with mixed diagnoses (n = 1), studies using in vitro/ex vivo techniques (n = 8), cross sectional comparisons of multiple psychiatric disorders without a control group (n = 5), no analysis of microglia/TSPO/kynurenines (n = 42), no quantification of microglia/TSPO/kynurenines (n = 4), kynurenines quantified only in blood/plasma (n = 13), and cytokine only studies (n = 2).

*Included:* 37 publications met eligibility and were included: 27 case control studies [19, 49, 86, 100, 102, 105, 116, 124, 125, 128, 133, 187-190, 192-194, 196-204] and 10 systematic reviews/meta‑analyses [145, 149, 153, 154, 158, 166, 205-208].

(The Boolean search string and full inclusion/exclusion details are provided in the figure and Supplementary Methods.)

**Supplementary Figure 3: Graphical overview of multimodal microglia-related evidence across the affective–psychosis spectrum.**

Three evidence pillars are compared across major depressive disorder (MDD), bipolar disorder (BD), and schizophrenia (SCZ): TSPO PET, postmortem microglial phenotyping, and the kynurenine pathway (KP). In MDD, the most reproducible signal is frontolimbic TSPO-PET elevation, whereas postmortem microglial and KP findings are subtler or region-dependent. In BD, evidence remains limited overall, with sparse PET data, predominantly null diagnosis-level postmortem findings, and mixed upstream/downstream KP alterations. In SCZ, TSPO-PET findings are heterogeneous, postmortem microglial phenotypes are marker-dependent, and the most consistent biochemical pattern is a shift toward the KYNA branch of the KP. Dashed borders and reduced opacity indicate limited evidence; question marks indicate heterogeneous or inconsistent findings. Overall, biological subgroups and symptom dimensions better explain the pattern than categorical diagnoses alone.

**References**

1.	Wakuda T, Yokokura M, Magata Y, Suzuki C, Murayama C, Goto T *et al.* α7 nicotinic acetylcholine receptor, activated glia, and cognitive impairment in schizophrenia: a dual-tracer PET study. *Molecular Psychiatry* 2025.

2.	Uranova NA, Vikhreva OV, Rakhmanova VI. Ultrastructural disturbances in microglia-neuron interactions in the head of the caudate nucleus in schizophrenia. *Eur Arch Psychiatry Clin Neurosci* 2025; **275**(3)**:** 823-838.

3.	Szabó A, Akkouh I, Requena-Osete JR, De Assis DR, Kondratskaya E, Hughes T *et al.* NLRP3 inflammasome mediates astroglial dysregulation of innate and adaptive immune responses in schizophrenia. *Brain, Behavior, and Immunity* 2025; **124:** 144-156.

4.	Smith RL, Mihalik A, Akula N, Auluck PK, Marenco S, Raznahan A *et al.* A neuro-immune axis of transcriptomic dysregulation within the subgenual anterior cingulate cortex in schizophrenia. *bioRxiv* 2025.

5.	Sager REH, North HF, Weissleder C, Clearwater MS, Walker AK, Fullerton JM *et al.* Divergent changes in complement pathway gene expression in schizophrenia and bipolar disorder: Links to inflammation and neurogenesis in the subependymal zone. *Schizophrenia Research* 2025; **275:** 25-34.

6.	Maccioni L, Brusaferri L, Barzon L, Schubert JJ, Nettis MA, Cousins O *et al.* A novel blood-free analytical framework for the quantification of neuroinflammatory load from TSPO PET Imaging. *Res Sq* 2025.

7.	Eiff B, Bullmore ET, Clatworthy MR, Fryer TD, Pariante CM, Mondelli V *et al.* Extra-axial inflammatory signal and its relationship to peripheral and central immunity in depression. *Brain* 2025; **148**(2)**:** 635-646.

8.	Vikhreva OV, Rakhmanova VI, Uranova NA. [Microglia-neuron interactions in the caudate nucleus in different course of schizophrenia]. *Zh Nevrol Psikhiatr Im S S Korsakova* 2024; **124**(7)**:** 154-164.

9.	Scheepstra K, Mizee M, Wever D, Hsiao CC, Zhang L, Swaab D *et al.* Reporting Psychiatric Disease Characteristics in Post-Mortem- and Biological Research. *Neurosci Insights* 2024; **19:** 26331055241252632.

10.	Reichert Plaska C, Heslegrave A, Bruno D, Ramos-Cejudo J, Han Lee S, Osorio R *et al.* Evidence for reduced anti-inflammatory microglial phagocytic response in late-life major depression. *Brain Behav Immun* 2024; **120:** 248-255.

11.	Rahimian R, Perlman K, Fakhfouri G, Mpai R, Richard VR, Hercher C *et al.* Proteomic evidence of depression-associated astrocytic dysfunction in the human male olfactory bulb. *Brain, Behavior, and Immunity* 2024; **122:** 110-121.

12.	Popovacki ES, Vogrinc D, Fuller HR, Horvat LL, Mayer D, Kopic J *et al.* Increased NLRP1 mRNA and Protein Expression Suggests Inflammasome Activation in the Dorsolateral Prefrontal and Medial Orbitofrontal Cortex in Schizophrenia. *Biomolecules* 2024; **14**(3).

13.	Nisha Aji K, Cisbani G, Weidenauer A, Koppel A, Hafizi S, Da Silva T *et al.* Neurofilament light-chain (NfL) and 18 kDa translocator protein in early psychosis and its putative high-risk. *Brain Behav Immun Health* 2024; **37:** 100742.

14.	Mendez-Victoriano G, Zhu YT, Middleton F, Ajulu K, Webster MJ, Weickert CS. Increased Parenchymal Macrophages are associated with decreased Tyrosine Hydroxylase mRNA levels in the Substantia Nigra of people with Schizophrenia and Bipolar Disorder. *Psychiatry Research* 2024; **340**.

15.	Gober R, Dallmeier J, Davis D, Brzostowicki D, de Rivero Vaccari JP, Cyr B *et al.* Increased inflammasome protein expression identified in microglia from postmortem brains with schizophrenia. *J Neuropathol Exp Neurol* 2024; **83**(11)**:** 951-966.

16.	Brown SJ, Christofides K, Weissleder C, Huang XF, Shannon Weickert C, Lim CK *et al.* Sex- and suicide-specific alterations in the kynurenine pathway in the anterior cingulate cortex in major depression. *Neuropsychopharmacology* 2024; **49**(3)**:** 584-592.

17.	Antenucci N, D'Errico G, Fazio F, Nicoletti F, Bruno V, Battaglia G. Changes in kynurenine metabolites in the gray and white matter of the dorsolateral prefrontal cortex of individuals affected by schizophrenia. *Schizophrenia (Heidelb)* 2024; **10**(1)**:** 27.

18.	Zhu Y, Webster MJ, Walker AK, Massa P, Middleton FA, Shannon Weickert CS. Increased prefrontal cortical cells positive for macrophage/microglial marker CD163 along blood vessels characterizes a neuropathology of neuroinflammatory schizophrenia. *Brain, Behavior, and Immunity* 2023; **111:** 46-60.

19.	Zheng H, Webster MJ, Weickert CS, Beasley CL, Paulus MP, Yolken RH *et al.* Cytomegalovirus antibodies are associated with mood disorders, suicide, markers of neuroinflammation, and microglia activation in postmortem brain samples. *Mol Psychiatry* 2023; **28**(12)**:** 5282-5292.

20.	Uranova NA, Vikhreva OV, Rakhmanova VI. Characteristics of the Interaction of Microglia and Oligodendrocytes in the White Matter in Continuous Schizophrenia. *Neuroscience and Behavioral Physiology* 2023; **53**(6)**:** 979-987.

21.	Uranova NA, Vikhreva OV, Rakhmanova VI. Microglia-neuron interactions in prefrontal gray matter in schizophrenia: a postmortem ultrastructural morphometric study. *Eur Arch Psychiatry Clin Neurosci* 2023; **273**(8)**:** 1633-1648.

22.	Uranova NA, Vikhreva OV. Microglial Reactivity in the Prefrontal Cortex in Schizophrenia. *Psychiatry (Moscow)* 2023; **21**(5)**:** 25-39.

23.	Sheikh MA, O'Connell KS, Lekva T, Szabo A, Akkouh IA, Osete JR *et al.* Systemic Cell Adhesion Molecules in Severe Mental Illness: Potential Role of Intercellular CAM-1 in Linking Peripheral and Neuroinflammation. *Biological Psychiatry* 2023; **93**(2)**:** 187-196.

24.	Scheepstra KWF, Mizee MR, van Scheppingen J, Adelia A, Wever DD, Mason MRJ *et al.* Microglia Transcriptional Profiling in Major Depressive Disorder Shows Inhibition of Cortical Gray Matter Microglia. *Biol Psychiatry* 2023; **94**(8)**:** 619-629.

25.	Sanadgol N, Miraki Feriz A, Lisboa SF, Joca SRL. Putative role of glial cells in treatment resistance depression: An updated critical literation review and evaluation of single-nuclei transcriptomics data. *Life Sciences* 2023; **331**.

26.	Jenkins AK, Lewis DA, Volk DW. Altered expression of microglial markers of phagocytosis in schizophrenia. *Schizophr Res* 2023; **251:** 22-29.

27.	Zhu Y, Webster MJ, Murphy CE, Middleton FA, Massa PT, Liu C *et al.* Distinct Phenotypes of Inflammation Associated Macrophages and Microglia in the Prefrontal Cortex Schizophrenia Compared to Controls. *Front Neurosci* 2022; **16:** 858989.

28.	Vikhreva OV, Uranova NA. Microglial Reactivity in the Prefrontal Cortex in Schizophrenia with Different Types of Course. *Neuroscience and Behavioral Physiology* 2022; **52**(5)**:** 639-644.

29.	Vikhreva OV, Rakhmanova VI, Uranova NA. Microglia-Oligodendrocyte Interactions in White Matter of the Prefrontal Cortex in Attack-Like Schizophrenia. *Psychiatry (Moscow)* 2022; **20**(4)**:** 84-95.

30.	Uranova NA, Vikhreva OV, Rakhmanova VI. [Specific interactions between microglia and oligodendrocytes in white matter in continuous schizophrenia]. *Zh Nevrol Psikhiatr Im S S Korsakova* 2022; **122**(12)**:** 128-137.

31.	Sakai M, Yu Z, Hirayama R, Nakasato M, Kikuchi Y, Ono C *et al.* Deficient Autophagy in Microglia Aggravates Repeated Social Defeat Stress-Induced Social Avoidance. *Neural Plast* 2022; **2022:** 7503553.

32.	Paul ER, Schwieler L, Erhardt S, Boda S, Trepci A, Kämpe R *et al.* Peripheral and central kynurenine pathway abnormalities in major depression. *Brain Behav Immun* 2022; **101:** 136-145.

33.	North HF, Weissleder C, Fullerton JM, Webster MJ, Weickert CS. Increased immune cell and altered microglia and neurogenesis transcripts in an Australian schizophrenia subgroup with elevated inflammation. *Schizophr Res* 2022; **248:** 208-218.

34.	Gober R, Ardalan M, Shiadeh SMJ, Duque L, Garamszegi SP, Ascona M *et al.* Microglia activation in postmortem brains with schizophrenia demonstrates distinct morphological changes between brain regions. *Brain Pathol* 2022; **32**(1)**:** e13003.

35.	Cakmak JD, Liu L, Poirier SE, Schaefer B, Poolacherla R, Burhan AM *et al.* The functional and structural associations of aberrant microglial activity in major depressive disorder. *J Psychiatry Neurosci* 2022; **47**(3)**:** E197-e208.

36.	Althubaity N, Schubert J, Martins D, Yousaf T, Nettis MA, Mondelli V *et al.* Choroid plexus enlargement is associated with neuroinflammation and reduction of blood brain barrier permeability in depression. *Neuroimage Clin* 2022; **33:** 102926.

37.	Zhang L, Verwer RWH, Zhao J, Huitinga I, Lucassen PJ, Swaab DF. Changes in glial gene expression in the prefrontal cortex in relation to major depressive disorder, suicide and psychotic features. *Journal of Affective Disorders* 2021; **295:** 893-903.

38.	Vikhreva OV, Uranova NA. [Microglial reactivity in the prefrontal cortex in different types of schizophrenia]. *Zh Nevrol Psikhiatr Im S S Korsakova* 2021; **121**(12)**:** 77-83.

39.	Uranova NA, Vikhreva OV, Rakhmanova VI. Abnormal microglial reactivity in gray matter of the prefrontal cortex in schizophrenia. *Asian J Psychiatr* 2021; **63:** 102752.

40.	Tzioras M, Stevenson AJ, Boche D, Spires-Jones TL. Microglial contribution to synaptic uptake in the prefrontal cortex in schizophrenia. *Neuropathol Appl Neurobiol* 2021; **47**(2)**:** 346-351.

41.	Turkheimer FE, Althubaity N, Schubert J, Nettis MA, Cousins O, Dima D *et al.* Increased serum peripheral C-reactive protein is associated with reduced brain barriers permeability of TSPO radioligands in healthy volunteers and depressed patients: implications for inflammation and depression. *Brain Behav Immun* 2021; **91:** 487-497.

42.	Teipel S, Bruno D, Plaska CR, Heslegrave A, Ramos-Cejudo J, Osorio RS *et al.* Association of CSF sTREM2, a marker of microglia activation, with cholinergic basal forebrain volume in major depressive disorder. *J Affect Disord* 2021; **293:** 429-434.

43.	Snijders G, Sneeboer MAM, Fernández-Andreu A, Udine E, Boks MP, Ormel PR *et al.* Distinct non-inflammatory signature of microglia in post-mortem brain tissue of patients with major depressive disorder. *Mol Psychiatry* 2021; **26**(7)**:** 3336-3349.

44.	Shimamoto-Mitsuyama C, Nakaya A, Esaki K, Balan S, Iwayama Y, Ohnishi T *et al.* Lipid Pathology of the Corpus Callosum in Schizophrenia and the Potential Role of Abnormal Gene Regulatory Networks with Reduced Microglial Marker Expression. *Cereb Cortex* 2021; **31**(1)**:** 448-462.

45.	Schubert JJ, Veronese M, Fryer TD, Manavaki R, Kitzbichler MG, Nettis MA *et al.* A Modest Increase in (11)C-PK11195-Positron Emission Tomography TSPO Binding in Depression Is Not Associated With Serum C-Reactive Protein or Body Mass Index. *Biol Psychiatry Cogn Neurosci Neuroimaging* 2021; **6**(7)**:** 716-724.

46.	Sager REH, Walker AK, Middleton F, Robinson K, Webster MJ, Weickert CS. Trajectory of change in brain complement factors from neonatal to young adult humans. *J Neurochem* 2021; **157**(3)**:** 479-493.

47.	Laurell GL, Plavén-Sigray P, Jucaite A, Varrone A, Cosgrove KP, Svarer C *et al.* Nondisplaceable Binding Is a Potential Confounding Factor in (11)C-PBR28 Translocator Protein PET Studies. *J Nucl Med* 2021; **62**(3)**:** 412-417.

48.	Joo YH, Lee MW, Son YD, Chang KA, Yaqub M, Kim HK *et al.* In Vivo Cerebral Translocator Protein (TSPO) Binding and Its Relationship with Blood Adiponectin Levels in Treatment-Naïve Young Adults with Major Depression: A [(11)C]PK11195 PET Study. *Biomedicines* 2021; **10**(1).

49.	Hill SL, Shao L, Beasley CL. Diminished levels of the chemokine fractalkine in post-mortem prefrontal cortex in schizophrenia but not bipolar disorder. *World J Biol Psychiatry* 2021; **22**(2)**:** 94-103.

50.	De Picker LJ, Victoriano GM, Richards R, Gorvett AJ, Lyons S, Buckland GR *et al.* Immune environment of the brain in schizophrenia and during the psychotic episode: A human post-mortem study. *Brain Behav Immun* 2021; **97:** 319-327.

51.	Da Silva T, Guma E, Hafizi S, Koppel A, Rusjan P, Kennedy JL *et al.* Genetically Predicted Brain C4A Expression Is Associated With TSPO and Hippocampal Morphology. *Biol Psychiatry* 2021; **90**(9)**:** 652-660.

52.	Conen S, Gregory CJ, Hinz R, Smallman R, Corsi-Zuelli F, Deakin B *et al.* Neuroinflammation as measured by positron emission tomography in patients with recent onset and established schizophrenia: implications for immune pathogenesis. *Mol Psychiatry* 2021; **26**(9)**:** 5398-5406.

53.	Afia AB, Vila È, MacDowell KS, Ormazabal A, Leza JC, Haro JM *et al.* Kynurenine pathway in post-mortem prefrontal cortex and cerebellum in schizophrenia: relationship with monoamines and symptomatology. *J Neuroinflammation* 2021; **18**(1)**:** 198.

54.	Uranova NA, Vikhreva OV, Rakhmanova VI, Orlovskaya DD. Dystrophy of Oligodendrocytes and Adjacent Microglia in Prefrontal Gray Matter in Schizophrenia. *Front Psychiatry* 2020; **11:** 204.

55.	Ulivieri M, Wierońska JM, Lionetto L, Martinello K, Cieslik P, Chocyk A *et al.* The Trace Kynurenine, Cinnabarinic Acid, Displays Potent Antipsychotic-Like Activity in Mice and Its Levels Are Reduced in the Prefrontal Cortex of Individuals Affected by Schizophrenia. *Schizophr Bull* 2020; **46**(6)**:** 1471-1481.

56.	Sneeboer MAM, van der Doef T, Litjens M, Psy NBB, Melief J, Hol EM *et al.* Microglial activation in schizophrenia: Is translocator 18 kDa protein (TSPO) the right marker? *Schizophr Res* 2020; **215:** 167-172.

57.	Shinko Y, Otsuka I, Okazaki S, Horai T, Boku S, Takahashi M *et al.* Chemokine alterations in the postmortem brains of suicide completers. *Journal of Psychiatric Research* 2020; **120:** 29-33.

58.	Schwieler L, Trepci A, Krzyzanowski S, Hermansson S, Granqvist M, Piehl F *et al.* A novel, robust method for quantification of multiple kynurenine pathway metabolites in the cerebrospinal fluid. *Bioanalysis* 2020; **12**(6)**:** 379-392.

59.	Rey R, Suaud-Chagny MF, Bohec AL, Dorey JM, d'Amato T, Tamouza R *et al.* Overexpression of complement component C4 in the dorsolateral prefrontal cortex, parietal cortex, superior temporal gyrus and associative striatum of patients with schizophrenia. *Brain Behavior and Immunity* 2020; **90:** 216-225.

60.	Kindler J, Lim CK, Weickert CS, Boerrigter D, Galletly C, Liu D *et al.* Dysregulation of kynurenine metabolism is related to proinflammatory cytokines, attention, and prefrontal cortex volume in schizophrenia. *Mol Psychiatry* 2020; **25**(11)**:** 2860-2872.

61.	Johansson V, Erhardt S, Engberg G, Kegel M, Bhat M, Schwieler L *et al.* Twin study shows association between monocyte chemoattractant protein-1 and kynurenic acid in cerebrospinal fluid. *Eur Arch Psychiatry Clin Neurosci* 2020; **270**(7)**:** 933-938.

62.	Gerritsen C, Iyengar Y, DaSilva T, Koppel A, Rusjan P, Bagby RM *et al.* Personality traits in psychosis and psychosis risk linked to TSPO expression: a neuroimmune marker. *Personal Neurosci* 2020; **3:** e14.

63.	Cai HQ, Catts VS, Webster MJ, Galletly C, Liu D, O'Donnell M *et al.* Increased macrophages and changed brain endothelial cell gene expression in the frontal cortex of people with schizophrenia displaying inflammation. *Mol Psychiatry* 2020; **25**(4)**:** 761-775.

64.	Böttcher C, Fernandez-Zapata C, Snijders GJL, Schlickeiser S, Sneeboer MAM, Kunkel D *et al.* Single-cell mass cytometry of microglia in major depressive disorder reveals a non-inflammatory phenotype with increased homeostatic marker expression. *Transl Psychiatry* 2020; **10**(1)**:** 310.

65.	Adorjan I, Sun B, Feher V, Tyler T, Veres D, Chance SA *et al.* Evidence for Decreased Density of Calretinin-Immunopositive Neurons in the Caudate Nucleus in Patients With Schizophrenia. *Front Neuroanat* 2020; **14:** 581685.

66.	Sellgren CM, Gracias J, Watmuff B, Biag JD, Thanos JM, Whittredge PB *et al.* Increased synapse elimination by microglia in schizophrenia patient-derived models of synaptic pruning. *Nat Neurosci* 2019; **22**(3)**:** 374-385.

67.	Schifani C, Hafizi S, Tseng HH, Gerritsen C, Kenk M, Wilson AA *et al.* Preliminary data indicating a connection between stress-induced prefrontal dopamine release and hippocampal TSPO expression in the psychosis spectrum. *Schizophr Res* 2019; **213:** 80-86.

68.	De Picker L, Ottoy J, Verhaeghe J, Deleye S, Wyffels L, Fransen E *et al.* State-associated changes in longitudinal [(18)F]-PBR111 TSPO PET imaging of psychosis patients: Evidence for the accelerated ageing hypothesis? *Brain Behav Immun* 2019; **77:** 46-54.

69.	Yrondi A, Aouizerate B, El-Hage W, Moliere F, Thalamas C, Delcourt N *et al.* Assessment of Translocator Protein Density, as Marker of Neuroinflammation, in Major Depressive Disorder: A Pilot, Multicenter, Comparative, Controlled, Brain PET Study (INFLADEP Study). *Front Psychiatry* 2018; **9:** 326.

70.	Xu LZ, Qi X, Zhu C, Wan LH. Activation of IL-8 and its participation in cancer in schizophrenia patients: new evidence for the autoimmune hypothesis of schizophrenia. *Neuropsychiatric Disease and Treatment* 2018; **14:** 3393-3403.

71.	Shakory S, Watts JJ, Hafizi S, Da Silva T, Khan S, Kiang M *et al.* Hippocampal glutamate metabolites and glial activation in clinical high risk and first episode psychosis. *Neuropsychopharmacology* 2018; **43**(11)**:** 2249-2255.

72.	Setiawan E, Attwells S, Wilson AA, Mizrahi R, Rusjan PM, Miler L *et al.* Association of translocator protein total distribution volume with duration of untreated major depressive disorder: a cross-sectional study. *Lancet Psychiatry* 2018; **5**(4)**:** 339-347.

73.	Selvaraj S, Bloomfield PS, Cao B, Veronese M, Turkheimer F, Howes OD. Brain TSPO imaging and gray matter volume in schizophrenia patients and in people at ultra high risk of psychosis: An [(11)C]PBR28 study. *Schizophr Res* 2018; **195:** 206-214.

74.	Richards EM, Zanotti-Fregonara P, Fujita M, Newman L, Farmer C, Ballard ED *et al.* PET radioligand binding to translocator protein (TSPO) is increased in unmedicated depressed subjects. *EJNMMI Res* 2018; **8**(1)**:** 57.

75.	Ottoy J, De Picker L, Verhaeghe J, Deleye S, Wyffels L, Kosten L *et al.* (18)F-PBR111 PET Imaging in Healthy Controls and Schizophrenia: Test-Retest Reproducibility and Quantification of Neuroinflammation. *J Nucl Med* 2018; **59**(8)**:** 1267-1274.

76.	Li H, Sagar AP, Keri S. Translocator protein (18kDa TSPO) binding, a marker of microglia, is reduced in major depression during cognitive-behavioral therapy. *Prog Neuropsychopharmacol Biol Psychiatry* 2018; **83:** 1-7.

77.	Li H, Sagar AP, Kéri S. Microglial markers in the frontal cortex are related to cognitive dysfunctions in major depressive disorder. *J Affect Disord* 2018; **241:** 305-310.

78.	Holmes SE, Hinz R, Conen S, Gregory CJ, Matthews JC, Anton-Rodriguez JM *et al.* Elevated Translocator Protein in Anterior Cingulate in Major Depression and a Role for Inflammation in Suicidal Thinking: A Positron Emission Tomography Study. *Biol Psychiatry* 2018; **83**(1)**:** 61-69.

79.	Hafizi S, Guma E, Koppel A, Da Silva T, Kiang M, Houle S *et al.* TSPO expression and brain structure in the psychosis spectrum. *Brain Behav Immun* 2018; **74:** 79-85.

80.	Kegel ME, Johansson V, Wetterberg L, Bhat M, Schwieler L, Cannon TD *et al.* Kynurenic acid and psychotic symptoms and personality traits in twins with psychiatric morbidity. *Psychiatry Res* 2017; **247:** 105-112.

81.	Johansson V, Jakobsson J, Fortgang RG, Zetterberg H, Blennow K, Cannon TD *et al.* Cerebrospinal fluid microglia and neurodegenerative markers in twins concordant and discordant for psychotic disorders. *Eur Arch Psychiatry Clin Neurosci* 2017; **267**(5)**:** 391-402.

82.	Hestad KA, Engedal K, Whist JE, Farup PG. The Relationships among Tryptophan, Kynurenine, Indoleamine 2,3-Dioxygenase, Depression, and Neuropsychological Performance. *Front Psychol* 2017; **8:** 1561.

83.	Hafizi S, Tseng HH, Rao N, Selvanathan T, Kenk M, Bazinet RP *et al.* Imaging Microglial Activation in Untreated First-Episode Psychosis: A PET Study With [(18)F]FEPPA. *Am J Psychiatry* 2017; **174**(2)**:** 118-124.

84.	Di Biase MA, Zalesky A, O'Keefe G, Laskaris L, Baune BT, Weickert CS *et al.* PET imaging of putative microglial activation in individuals at ultra-high risk for psychosis, recently diagnosed and chronically ill with schizophrenia. *Transl Psychiatry* 2017; **7**(8)**:** e1225.

85.	Collste K, Plavén-Sigray P, Fatouros-Bergman H, Victorsson P, Schain M, Forsberg A *et al.* Lower levels of the glial cell marker TSPO in drug-naive first-episode psychosis patients as measured using PET and [(11)C]PBR28. *Mol Psychiatry* 2017; **22**(6)**:** 850-856.

86.	Brisch R, Steiner J, Mawrin C, Krzyzanowska M, Jankowski Z, Gos T. Microglia in the dorsal raphe nucleus plays a potential role in both suicide facilitation and prevention in affective disorders. *Eur Arch Psychiatry Clin Neurosci* 2017; **267**(5)**:** 403-415.

87.	van der Doef TF, de Witte LD, Sutterland AL, Jobse E, Yaqub M, Boellaard R *et al.* In vivo (R)-[(11)C]PK11195 PET imaging of 18kDa translocator protein in recent onset psychosis. *NPJ Schizophr* 2016; **2:** 16031.

88.	Su L, Faluyi YO, Hong YT, Fryer TD, Mak E, Gabel S *et al.* Neuroinflammatory and morphological changes in late-life depression: the NIMROD study. *Br J Psychiatry* 2016; **209**(6)**:** 525-526.

89.	Steiner J, Brisch R, Schiltz K, Dobrowolny H, Mawrin C, Krzyżanowska M *et al.* GABAergic system impairment in the hippocampus and superior temporal gyrus of patients with paranoid schizophrenia: A post-mortem study. *Schizophrenia Research* 2016; **177**(1-3)**:** 10-17.

90.	Holmes SE, Hinz R, Drake RJ, Gregory CJ, Conen S, Matthews JC *et al.* In vivo imaging of brain microglial activity in antipsychotic-free and medicated schizophrenia: a [(11)C](R)-PK11195 positron emission tomography study. *Mol Psychiatry* 2016; **21**(12)**:** 1672-1679.

91.	Coughlin JM, Wang Y, Ambinder EB, Ward RE, Minn I, Vranesic M *et al.* In vivo markers of inflammatory response in recent-onset schizophrenia: a combined study using [(11)C]DPA-713 PET and analysis of CSF and plasma. *Transl Psychiatry* 2016; **6**(4)**:** e777.

92.	Clark SM, Pocivavsek A, Nicholson JD, Notarangelo FM, Langenberg P, McMahon RP *et al.* Reduced kynurenine pathway metabolism and cytokine expression in the prefrontal cortex of depressed individuals. *J Psychiatry Neurosci* 2016; **41**(6)**:** 386-394.

93.	Brundin L, Sellgren CM, Lim CK, Grit J, Pålsson E, Landén M *et al.* An enzyme in the kynurenine pathway that governs vulnerability to suicidal behavior by regulating excitotoxicity and neuroinflammation. *Transl Psychiatry* 2016; **6**(8)**:** e865.

94.	Bloomfield PS, Selvaraj S, Veronese M, Rizzo G, Bertoldo A, Owen DR *et al.* Microglial Activity in People at Ultra High Risk of Psychosis and in Schizophrenia: An [(11)C]PBR28 PET Brain Imaging Study. *Am J Psychiatry* 2016; **173**(1)**:** 44-52.

95.	Setiawan E, Wilson AA, Mizrahi R, Rusjan PM, Miler L, Rajkowska G *et al.* Role of translocator protein density, a marker of neuroinflammation, in the brain during major depressive episodes. *JAMA Psychiatry* 2015; **72**(3)**:** 268-275.

96.	Selvaraj S, Bloomfield P, Veronese M, Rizzo G, Bertoldo A, Owen DR *et al.* Microglial Activity in People at Ultra High Risk of Psychosis and in Schizophrenia: An 11C PBR28 PET Brain Imaging Study. *Biological Psychiatry* 2015; **77**(9).

97.	Schwieler L, Larsson MK, Skogh E, Kegel ME, Orhan F, Abdelmoaty S *et al.* Increased levels of IL-6 in the cerebrospinal fluid of patients with chronic schizophrenia--significance for activation of the kynurenine pathway. *J Psychiatry Neurosci* 2015; **40**(2)**:** 126-133.

98.	Richards EM, Zanotti-Fregonara P, Fujita M, Walls T, Niciu MJ, Machado-Vieira R *et al.* Increased PET Radioligand Binding to Translocator Protein Suggests that Major Depressive Disorder Is Associated with Neuroinflammation. *Biological Psychiatry* 2015; **77**(9).

99.	Kenk M, Selvanathan T, Rao N, Suridjan I, Rusjan P, Remington G *et al.* Imaging neuroinflammation in gray and white matter in schizophrenia: an in-vivo PET study with [18F]-FEPPA. *Schizophr Bull* 2015; **41**(1)**:** 85-93.

100.	Busse M, Busse S, Myint AM, Gos T, Dobrowolny H, Muller UJ *et al.* Decreased quinolinic acid in the hippocampus of depressive patients: evidence for local anti-inflammatory and neuroprotective responses? *Eur Arch Psychiatry Clin Neurosci* 2015; **265**(4)**:** 321-329.

101.	Bay-Richter C, Linderholm KR, Lim CK, Samuelsson M, Träskman-Bendz L, Guillemin GJ *et al.* A role for inflammatory metabolites as modulators of the glutamate N-methyl-D-aspartate receptor in depression and suicidality. *Brain Behav Immun* 2015; **43:** 110-117.

102.	Wesseling H, Gottschalk MG, Bahn S. Targeted multiplexed selected reaction monitoring analysis evaluates protein expression changes of molecular risk factors for major psychiatric disorders. *Int J Neuropsychopharmacol* 2014; **18**(1).

103.	Torres-Platas SG, Cruceanu C, Chen GG, Turecki G, Mechawar N. Evidence for increased microglial priming and macrophage recruitment in the dorsal anterior cingulate white matter of depressed suicides. *Brain Behav Immun* 2014; **42:** 50-59.

104.	Kegel ME, Bhat M, Skogh E, Samuelsson M, Lundberg K, Dahl ML *et al.* Imbalanced kynurenine pathway in schizophrenia. *Int J Tryptophan Res* 2014; **7:** 15-22.

105.	Hercher C, Chopra V, Beasley CL. Evidence for morphological alterations in prefrontal white matter glia in schizophrenia and bipolar disorder. *J Psychiatry Neurosci* 2014; **39**(6)**:** 376-385.

106.	Gos T, Myint AM, Schiltz K, Meyer-Lotz G, Dobrowolny H, Busse S *et al.* Reduced microglial immunoreactivity for endogenous NMDA receptor agonist quinolinic acid in the hippocampus of schizophrenia patients. *Brain Behav Immun* 2014; **41:** 59-64.

107.	Sasayama D, Hattori K, Wakabayashi C, Teraishi T, Hori H, Ota M *et al.* Increased cerebrospinal fluid interleukin-6 levels in patients with schizophrenia and those with major depressive disorder. *Journal of Psychiatric Research* 2013; **47**(3)**:** 401-406.

108.	Kreisl WC, Jenko KJ, Hines CS, Lyoo CH, Corona W, Morse CL *et al.* A genetic polymorphism for translocator protein 18 kDa affects both in vitro and in vivo radioligand binding in human brain to this putative biomarker of neuroinflammation. *J Cereb Blood Flow Metab* 2013; **33**(1)**:** 53-58.

109.	Johansson AS, Owe-Larsson B, Asp L, Kocki T, Adler M, Hetta J *et al.* Activation of kynurenine pathway in ex vivo fibroblasts from patients with bipolar disorder or schizophrenia: Cytokine challenge increases production of 3-hydroxykynurenine. *Journal of Psychiatric Research* 2013; **47**(11)**:** 1815-1823.

110.	Hannestad J, DellaGioia N, Gallezot JD, Lim K, Nabulsi N, Esterlis I *et al.* The neuroinflammation marker translocator protein is not elevated in individuals with mild-to-moderate depression: a [¹¹C]PBR28 PET study. *Brain Behav Immun* 2013; **33:** 131-138.

111.	Roussos P, Katsel P, Davis KL, Siever LJ, Haroutunian V. A system-level transcriptomic analysis of schizophrenia using postmortem brain tissue samples. *Arch Gen Psychiatry* 2012; **69**(12)**:** 1205-1213.

112.	Linderholm KR, Skogh E, Olsson SK, Dahl ML, Holtze M, Engberg G *et al.* Increased levels of kynurenine and kynurenic acid in the CSF of patients with schizophrenia. *Schizophr Bull* 2012; **38**(3)**:** 426-432.

113.	Holtze M, Saetre P, Engberg G, Schwieler L, Werge T, Andreassen OA *et al.* Kynurenine 3-monooxygenase polymorphisms: relevance for kynurenic acid synthesis in patients with schizophrenia and healthy controls. *J Psychiatry Neurosci* 2012; **37**(1)**:** 53-57.

114.	Busse S, Busse M, Schiltz K, Bielau H, Gos T, Brisch R *et al.* Different distribution patterns of lymphocytes and microglia in the hippocampus of patients with residual versus paranoid schizophrenia: further evidence for disease course-related immune alterations? *Brain Behav Immun* 2012; **26**(8)**:** 1273-1279.

115.	Wonodi I, Stine OC, Sathyasaikumar KV, Roberts RC, Mitchell BD, Hong LE *et al.* Downregulated kynurenine 3-monooxygenase gene expression and enzyme activity in schizophrenia and genetic association with schizophrenia endophenotypes. *Arch Gen Psychiatry* 2011; **68**(7)**:** 665-674.

116.	Steiner J, Walter M, Gos T, Guillemin GJ, Bernstein HG, Sarnyai Z *et al.* Severe depression is associated with increased microglial quinolinic acid in subregions of the anterior cingulate gyrus: evidence for an immune-modulated glutamatergic neurotransmission? *J Neuroinflammation* 2011; **8:** 94.

117.	Schröder J, Karlsson H, Yolken R, Torrey F, Essig M. HERV-W RELATED RNA IN CEREBROSPINAL FLUID AND KYNURENIC ACID LEVELS IN PATIENTS WITH FIRST-EPISODE SCHIZOPHRENIA SPECTRUM DISORDER - IMPACT ON BRAIN CHANGES. *Schizophrenia Bulletin* 2011; **37:** 127-128.

118.	Sathyasaikumar KV, Stachowski EK, Wonodi I, Roberts RC, Rassoulpour A, McMahon RP *et al.* Impaired kynurenine pathway metabolism in the prefrontal cortex of individuals with schizophrenia. *Schizophr Bull* 2011; **37**(6)**:** 1147-1156.

119.	Takano A, Arakawa R, Ito H, Tateno A, Takahashi H, Matsumoto R *et al.* Peripheral benzodiazepine receptors in patients with chronic schizophrenia: A PET study with [11C]DAA1106. *International Journal of Neuropsychopharmacology* 2010; **13**(7)**:** 943-950.

120.	Raison CL, Dantzer R, Kelley KW, Lawson MA, Woolwine BJ, Vogt G *et al.* CSF concentrations of brain tryptophan and kynurenines during immune stimulation with IFN-alpha: relationship to CNS immune responses and depression. *Mol Psychiatry* 2010; **15**(4)**:** 393-403.

121.	Doorduin J, de Vries EF, Willemsen AT, de Groot JC, Dierckx RA, Klein HC. Neuroinflammation in schizophrenia-related psychosis: a PET study. *J Nucl Med* 2009; **50**(11)**:** 1801-1807.

122.	Barry S, Clarke G, Scully P, Dinan TG. Kynurenine pathway in psychosis: evidence of increased tryptophan degradation. *J Psychopharmacol* 2009; **23**(3)**:** 287-294.

123.	van Berckel BN, Bossong MG, Boellaard R, Kloet R, Schuitemaker A, Caspers E *et al.* Microglia activation in recent-onset schizophrenia: a quantitative (R)-[11C]PK11195 positron emission tomography study. *Biol Psychiatry* 2008; **64**(9)**:** 820-822.

124.	Steiner J, Bielau H, Brisch R, Danos P, Ullrich O, Mawrin C *et al.* Immunological aspects in the neurobiology of suicide: elevated microglial density in schizophrenia and depression is associated with suicide. *J Psychiatr Res* 2008; **42**(2)**:** 151-157.

125.	Miller CL, Llenos IC, Cwik M, Walkup J, Weis S. Alterations in kynurenine precursor and product levels in schizophrenia and bipolar disorder. *Neurochem Int* 2008; **52**(6)**:** 1297-1303.

126.	Karperien AL, Jelinek HF, Buchan AM. Box-counting analysis of microglia form in schizophrenia, alzheimer's disease and affective disorder. *Fractals* 2008; **16**(2)**:** 103-107.

127.	Steiner J, Mawrin C, Ziegeler A, Bielau H, Ullrich O, Bernstein HG *et al.* Distribution of HLA-DR-positive microglia in schizophrenia reflects impaired cerebral lateralization. *Acta Neuropathol* 2006; **112**(3)**:** 305-316.

128.	Miller CL, Llenos IC, Dulay JR, Weis S. Upregulation of the initiating step of the kynurenine pathway in postmortem anterior cingulate cortex from individuals with schizophrenia and bipolar disorder. *Brain Res* 2006; **1073-1074:** 25-37.

129.	Kapoor R, Lim KS, Cheng A, Garrick T, Kapoor V. Preliminary evidence for a link between schizophrenia and NMDA-glycine site receptor ligand metabolic enzymes, d-amino acid oxidase (DAAO) and kynurenine aminotransferase-1 (KAT-1). *Brain Res* 2006; **1106**(1)**:** 205-210.

130.	Wierzba-Bobrowicz T, Lewandowska E, Lechowicz W, Stepien T, Pasennik E. Quantitative analysis of activated microglia, ramified and damage of processes in the frontal and temporal lobes of chronic schizophrenics. *Folia Neuropathol* 2005; **43**(2)**:** 81-89.

131.	Nilsson LK, Linderholm KR, Engberg G, Paulson L, Blennow K, Lindström LH *et al.* Elevated levels of kynurenic acid in the cerebrospinal fluid of male patients with schizophrenia. *Schizophr Res* 2005; **80**(2-3)**:** 315-322.

132.	Miller CL, Llenos IC, Dulay JR, Barillo MM, Yolken RH, Weis S. Expression of the kynurenine pathway enzyme tryptophan 2,3-dioxygenase is increased in the frontal cortex of individuals with schizophrenia. *Neurobiol Dis* 2004; **15**(3)**:** 618-629.

133.	Hamidi M, Drevets WC, Price JL. Glial reduction in amygdala in major depressive disorder is due to oligodendrocytes. *Biol Psychiatry* 2004; **55**(6)**:** 563-569.

134.	Thomas AJ, Perry R, Barber R, Kalaria RN, O'Brien JT. Pathologies and pathological mechanisms for white matter hyperintensities in depression. *Alzheimer's Disease: Vascular Etiology and Pathology* 2002; **977:** 333-339.

135.	Thomas AJ, O'Brien JT, Davis S, Ballard C, Barber R, Kalaria RN *et al.* Ischemic basis for deep white matter hyperintensities in major depression: a neuropathological study. *Arch Gen Psychiatry* 2002; **59**(9)**:** 785-792.

136.	Schwarcz R, Rassoulpour A, Wu HQ, Medoff D, Tamminga CA, Roberts RC. Increased cortical kynurenate content in schizophrenia. *Biol Psychiatry* 2001; **50**(7)**:** 521-530.

137.	Erhardt S, Blennow K, Nordin C, Skogh E, Lindström LH, Engberg G. Kynurenic acid levels are elevated in the cerebrospinal fluid of patients with schizophrenia. *Neurosci Lett* 2001; **313**(1-2)**:** 96-98.

138.	Radewicz K, Garey LJ, Gentleman SM, Reynolds R. Increase in HLA-DR immunoreactive microglia in frontal and temporal cortex of chronic schizophrenics. *J Neuropathol Exp Neurol* 2000; **59**(2)**:** 137-150.

139.	Falke E, Han LY, Arnold SE. Absence of neurodegeneration in the thalamus and caudate of elderly patients with schizophrenia. *Psychiatry Research* 2000; **93**(2)**:** 103-110.

140.	Torrey EF, Yolken RH, Zito M, Heyes M. Increased CSF and brain quinolinic acid in schizophrenia and bipolar disorder. *Schizophrenia Research* 1998; **29**(1-2)**:** 91-92.

141.	Schwarcz R, Wu HQ, Rassoulpour A, Tamminga CA, Medoff DR, Roberts RC. Post-mortem determination of kynurenine and kynurenic acid levels in the brains of schizophrenic patients. *Schizophrenia Research* 1997; **24**(1-2)**:** 36-36.

142.	Jospeh MH, Baker HF, Crow TJ, Riley GJ, Risby D. Brain tryptophan metabolism in schizophrenia: a post mortem study of metabolites of the serotonin and kynurenine pathways in schizophrenic and control subjects. *Psychopharmacology (Berl)* 1979; **62**(3)**:** 279-285.

143.	Yamamoto M, Sakai M, Yu Z, Nakanishi M, Yoshii H. Glial Markers of Suicidal Behavior in the Human Brain-A Systematic Review of Postmortem Studies. *Int J Mol Sci* 2024; **25**(11).

144.	Wang L, Feng Z, Zheng T, Dai G, Wang M, Zhou L *et al.* Associations between the kynurenine pathway and the brain in patients with major depressive disorder-A systematic review of neuroimaging studies. *Prog Neuropsychopharmacol Biol Psychiatry* 2023; **121:** 110675.

145.	Sales PMG, Schrage E, Coico R, Pato M. Linking nervous and immune systems in psychiatric illness: A meta-analysis of the kynurenine pathway. *Brain Research* 2023; **1800**.

146.	Rømer TB, Jeppesen R, Christensen RHB, Benros ME. Biomarkers in the cerebrospinal fluid of patients with psychotic disorders compared to healthy controls: a systematic review and meta-analysis. *Mol Psychiatry* 2023; **28**(6)**:** 2277-2290.

147.	Inam ME, Fernandes BS, Salagre E, Grande I, Vieta E, Quevedo J *et al.* The kynurenine pathway in major depressive disorder, bipolar disorder, and schizophrenia: a systematic review and meta-analysis of cerebrospinal fluid studies. *Braz J Psychiatry* 2023; **45**(4)**:** 343-355.

148.	De Picker LJ, Morrens M, Branchi I, Haarman BCM, Terada T, Kang MS *et al.* TSPO PET brain inflammation imaging: A transdiagnostic systematic review and meta-analysis of 156 case-control studies. *Brain Behav Immun* 2023; **113:** 415-431.

149.	Liu SH, Du Y, Chen L, Cheng Y. Glial Cell Abnormalities in Major Psychiatric Diseases: A Systematic Review of Postmortem Brain Studies. *Mol Neurobiol* 2022; **59**(3)**:** 1665-1692.

150.	Eggerstorfer B, Kim JH, Cumming P, Lanzenberger R, Gryglewski G. Meta-analysis of molecular imaging of translocator protein in major depression. *Front Mol Neurosci* 2022; **15:** 981442.

151.	de Bartolomeis A, Barone A, Vellucci L, Mazza B, Austin MC, Iasevoli F *et al.* Linking Inflammation, Aberrant Glutamate-Dopamine Interaction, and Post-synaptic Changes: Translational Relevance for Schizophrenia and Antipsychotic Treatment: a Systematic Review. *Mol Neurobiol* 2022; **59**(10)**:** 6460-6501.

152.	Almulla AF, Vasupanrajit A, Tunvirachaisakul C, Al-Hakeim HK, Solmi M, Verkerk R *et al.* The tryptophan catabolite or kynurenine pathway in schizophrenia: meta-analysis reveals dissociations between central, serum, and plasma compartments. *Molecular Psychiatry* 2022.

153.	Almulla AF, Thipakorn Y, Vasupanrajit A, Tunvirachaisakul C, Oxenkrug G, Al-Hakeim HK *et al.* The Tryptophan Catabolite or Kynurenine Pathway in a Major Depressive Episode with Melancholia, Psychotic Features and Suicidal Behaviors: A Systematic Review and Meta-Analysis. *Cells* 2022; **11**(19).

154.	Almulla AF, Thipakorn Y, Vasupanrajit A, Abo Algon AA, Tunvirachaisakul C, Aljanabi AA *et al.* The tryptophan catabolite or kynurenine pathway in major depressive and bipolar disorder: A systematic review and meta-analysis. *Brain, Behavior, and Immunity - Health* 2022; **26**.

155.	Snijders G, van Zuiden W, Sneeboer MAM, Berdenis van Berlekom A, van der Geest AT, Schnieder T *et al.* A loss of mature microglial markers without immune activation in schizophrenia. *Glia* 2021; **69**(5)**:** 1251-1267.

156.	Skorobogatov K, De Picker L, Verkerk R, Coppens V, Leboyer M, Muller N *et al.* Brain Versus Blood: A Systematic Review on the Concordance Between Peripheral and Central Kynurenine Pathway Measures in Psychiatric Disorders. *Front Immunol* 2021; **12:** 716980.

157.	Plaven-Sigray P, Matheson GJ, Coughlin JM, Hafizi S, Laurikainen H, Ottoy J *et al.* Meta-analysis of the Glial Marker TSPO in Psychosis Revisited: Reconciling Inconclusive Findings of Patient-Control Differences. *Biol Psychiatry* 2021; **89**(3)**:** e5-e8.

158.	Marx W, McGuinness AJ, Rocks T, Ruusunen A, Cleminson J, Walker AJ *et al.* The kynurenine pathway in major depressive disorder, bipolar disorder, and schizophrenia: a meta-analysis of 101 studies. *Molecular Psychiatry* 2021; **26**(8)**:** 4158-4178.

159.	Chauveau F, Becker G, Boutin H. Have (R)-[11C]PK11195 challengers fulfilled the promise? A scoping review of clinical TSPO PET studies. *European Journal of Nuclear Medicine and Molecular Imaging* 2021; **49**(1)**:** 201-220.

160.	Cao B, Chen Y, Ren Z, Pan Z, McIntyre RS, Wang D. Dysregulation of kynurenine pathway and potential dynamic changes of kynurenine in schizophrenia: A systematic review and meta-analysis. *Neurosci Biobehav Rev* 2021; **123:** 203-214.

161.	Plavén-Sigray P, Matheson G, Coughlin J, Hafizi S, Laurikainen H, Ottoy J *et al.* UPDATED INDIVIDUAL PARTICIPANT DATA META-ANALYSIS CONFIRMS LOWER LEVELS OF THE GLIAL MARKER TSPO IN PSYCHOSIS PATIENTS. *Schizophrenia Bulletin* 2020; **46:** S233-S233.

162.	Pedraz-Petrozzi B, Elyamany O, Rummel C, Mulert C. Effects of inflammation on the kynurenine pathway in schizophrenia - a systematic review. *J Neuroinflammation* 2020; **17**(1)**:** 56.

163.	Plavén-Sigray P, Cervenka S. Meta-analytic studies of the glial cell marker TSPO in psychosis - a question of apples and pears? A commentary on 'Neuroinflammation in schizophrenia: metaanalysis of in-vivo microglial imaging' by Marques et al. *Psychological Medicine* 2019; **49**(10)**:** 1624-1628.

164.	Marques TR, Ashok AH, Pillinger T, Veronese M, Turkheimer FE, Dazzan P *et al.* Neuroinflammation in schizophrenia: meta-analysis of in vivo microglial imaging studies. *Psychol Med* 2019; **49**(13)**:** 2186-2196.

165.	Enache D, Pariante CM, Mondelli V. Markers of central inflammation in major depressive disorder: A systematic review and meta-analysis of studies examining cerebrospinal fluid, positron emission tomography and post-mortem brain tissue. *Brain Behav Immun* 2019; **81:** 24-40.

166.	Wang AK, Miller BJ. Meta-analysis of Cerebrospinal Fluid Cytokine and Tryptophan Catabolite Alterations in Psychiatric Patients: Comparisons Between Schizophrenia, Bipolar Disorder, and Depression. *Schizophr Bull* 2018; **44**(1)**:** 75-83.

167.	Plaven-Sigray P, Matheson GJ, Collste K, Ashok AH, Coughlin JM, Howes OD *et al.* Positron Emission Tomography Studies of the Glial Cell Marker Translocator Protein in Patients With Psychosis: A Meta-analysis Using Individual Participant Data. *Biol Psychiatry* 2018; **84**(6)**:** 433-442.

168.	Ogyu K, Kubo K, Noda Y, Iwata Y, Tsugawa S, Omura Y *et al.* Kynurenine pathway in depression: A systematic review and meta-analysis. *Neuroscience and Biobehavioral Reviews* 2018; **90:** 16-25.

169.	van Kesteren CF, Gremmels H, de Witte LD, Hol EM, Van Gool AR, Falkai PG *et al.* Immune involvement in the pathogenesis of schizophrenia: a meta-analysis on postmortem brain studies. *Transl Psychiatry* 2017; **7**(3)**:** e1075.

170.	Plitman E, Iwata Y, Caravaggio F, Nakajima S, Chung JK, Gerretsen P *et al.* Kynurenic Acid in Schizophrenia: A Systematic Review and Meta-analysis. *Schizophr Bull* 2017; **43**(4)**:** 764-777.

171.	Trépanier MO, Hopperton KE, Mizrahi R, Mechawar N, Bazinet RP. Postmortem evidence of cerebral inflammation in schizophrenia: a systematic review. *Mol Psychiatry* 2016; **21**(8)**:** 1009-1026.

172.	Byrne ML, Whittle S, Allen NB. The Role of Brain Structure and Function in the Association Between Inflammation and Depressive Symptoms: A Systematic Review. *Psychosomatic Medicine* 2016; **78**(4)**:** 389-400.

173.	Bergon A, Belzeaux R, Comte M, Pelletier F, Hervé M, Gardiner EJ *et al.* CX3CR1 is dysregulated in blood and brain from schizophrenia patients. *Schizophr Res* 2015; **168**(1-2)**:** 434-443.

174.	Hannestad J, DellaGioia N, Gallezot JD, Lim K, Nabulsi N, Esterlis I *et al.* The neuroinflammation marker translocator protein is not elevated in individuals with mild-to-moderate depression: a [(1)(1)C]PBR28 PET study. *Brain Behav Immun* 2013; **33:** 131-138.

175.	Gritti D, Delvecchio G, Ferro A, Bressi C, Brambilla P. Neuroinflammation in Major Depressive Disorder: A Review of PET Imaging Studies Examining the 18-kDa Translocator Protein. *J Affect Disord* 2021; **292:** 642-651.

176.	Attwells S, Setiawan E, Rusjan PM, Xu C, Hutton C, Rafiei D *et al.* Translocator Protein Distribution Volume Predicts Reduction of Symptoms During Open-Label Trial of Celecoxib in Major Depressive Disorder. *Biol Psychiatry* 2020; **88**(8)**:** 649-656.

177.	Collste K, Plaven-Sigray P, Fatouros-Bergman H, Victorsson P, Schain M, Forsberg A *et al.* Lower levels of the glial cell marker TSPO in drug-naive first-episode psychosis patients as measured using PET and [(11)C]PBR28. *Mol Psychiatry* 2017; **22**(6)**:** 850-856.

178.	Laurikainen H, Vuorela A, Toivonen A, Reinert-Hartwall L, Trontti K, Lindgren M *et al.* Elevated serum chemokine CCL22 levels in first-episode psychosis: associations with symptoms, peripheral immune state and in vivo brain glial cell function. *Transl Psychiatry* 2020; **10**(1)**:** 94.

179.	Schubert JJ, Veronese M, Fryer TD, Manavaki R, Kitzbichler MG, Nettis MA *et al.* A Modest Increase in (11)C-PK11195-Positron Emission Tomography TSPO Binding in Depression Is Not Associated With Serum C-Reactive Protein or Body Mass Index. *Biol Psychiatry Cogn Neurosci Neuroimaging* 2021; **6**(7)**:** 716-724.

180.	J. SH, E. VG, T. HJP, N. S, J. DJ, P. G *et al.* Interpreting results and drawing conclusions [last updated August 2023]. In: T. HJP, J. T, J. C, M. C, T. L, J. PM *et al.* (eds). *Cochrane Handbook for Systematic Reviews of Interventions*, version 6.5. edn. Cochrane2024, p Available from cochrane.org/handbook.

181.	Borenstein MH, Larry V.; Higgins, Julian P. T.; Rothstein, Hannah R. Subgroup analyses. *Introduction to meta-analysis*. John Wiley & Sons Inc: Hoboken, NJ, 2009, pp 149-186.

182.	Foster R, Kandanearatchi A, Beasley C, Williams B, Khan N, Fagerhol MK *et al.* Calprotectin in microglia from frontal cortex is up-regulated in schizophrenia: evidence for an inflammatory process? *Eur J Neurosci* 2006; **24**(12)**:** 3561-3566.

183.	Dean B, Gibbons AS, Tawadros N, Brooks L, Everall IP, Scarr E. Different changes in cortical tumor necrosis factor-alpha-related pathways in schizophrenia and mood disorders. *Mol Psychiatry* 2013; **18**(7)**:** 767-773.

184.	Loef D, Tendolkar I, van Eijndhoven PFP, Hoozemans JJM, Oudega ML, Rozemuller AJM *et al.* Electroconvulsive therapy is associated with increased immunoreactivity of neuroplasticity markers in the hippocampus of depressed patients. *Transl Psychiatry* 2023; **13**(1)**:** 355.

185.	Pandey HS, Kapoor R, Bindu, Seth P. Coronin 1A facilitates calcium mobilization and promotes astrocyte reactivity in HIV-1 neuropathogenesis. *FASEB Bioadv* 2022; **4**(4)**:** 254-272.

186.	Gatfield J, Albrecht I, Zanolari B, Steinmetz MO, Pieters J. Association of the leukocyte plasma membrane with the actin cytoskeleton through coiled coil-mediated trimeric coronin 1 molecules. *Mol Biol Cell* 2005; **16**(6)**:** 2786-2798.

187.	Naggan L, Robinson E, Dinur E, Goldenberg H, Kozela E, Yirmiya R. Suicide in bipolar disorder patients is associated with hippocampal microglia activation and reduction of lymphocytes-activation gene 3 (LAG3) microglial checkpoint expression. *Brain Behav Immun* 2023; **110:** 185-194.

188.	Petrasch-Parwez E, Schobel A, Benali A, Moinfar Z, Forster E, Brune M *et al.* Lateralization of increased density of Iba1-immunopositive microglial cells in the anterior midcingulate cortex of schizophrenia and bipolar disorder. *Eur Arch Psychiatry Clin Neurosci* 2020; **270**(7)**:** 819-828.

189.	Rao JS, Harry GJ, Rapoport SI, Kim HW. Increased excitotoxicity and neuroinflammatory markers in postmortem frontal cortex from bipolar disorder patients. *Mol Psychiatry* 2010; **15**(4)**:** 384-392.

190.	Seredenina T, Sorce S, Herrmann FR, Ma Mulone XJ, Plastre O, Aguzzi A *et al.* Decreased NOX2 expression in the brain of patients with bipolar disorder: association with valproic acid prescription and substance abuse. *Transl Psychiatry* 2017; **7**(8)**:** e1206.

191.	Sneeboer MAM, Snijders G, Berdowski WM, Fernandez-Andreu A, Psychiatric Donor Program of the Netherlands Brain B, van Mierlo HC *et al.* Microglia in post-mortem brain tissue of patients with bipolar disorder are not immune activated. *Transl Psychiatry* 2019; **9**(1)**:** 153.

192.	Zhang L, Verwer RWH, Lucassen PJ, Huitinga I, Swaab DF. Sex difference in glia gene expression in the dorsolateral prefrontal cortex in bipolar disorder: Relation to psychotic features. *J Psychiatr Res* 2020; **125:** 66-74.

193.	Connor CM, Guo Y, Akbarian S. Cingulate white matter neurons in schizophrenia and bipolar disorder. *Biol Psychiatry* 2009; **66**(5)**:** 486-493.

194.	Lavebratt C, Olsson S, Backlund L, Frisen L, Sellgren C, Priebe L *et al.* The KMO allele encoding Arg452 is associated with psychotic features in bipolar disorder type 1, and with increased CSF KYNA level and reduced KMO expression. *Mol Psychiatry* 2014; **19**(3)**:** 334-341.

195.	Afia AB, Vila E, MacDowell KS, Ormazabal A, Leza JC, Haro JM *et al.* Kynurenine pathway in post-mortem prefrontal cortex and cerebellum in schizophrenia: relationship with monoamines and symptomatology. *J Neuroinflammation* 2021; **18**(1)**:** 198.

196.	Dean B, Gibbons AS, Tawadros N, Brooks L, Everall IP, Scarr E. Different changes in cortical tumor necrosis factor-α-related pathways in schizophrenia and mood disorders. *Molecular Psychiatry* 2013; **18**(7)**:** 767-773.

197.	Foster R, Kandanearatchi A, Beasley CL, Williams B, Khan N, Fagerhol MK *et al.* Calprotectin in microglia from frontal cortex is up-regulated in schizophrenia: Evidence for an inflammatory process? *European Journal of Neuroscience* 2006; **24**(12)**:** 3561-3566.

198.	Haarman BC, Riemersma-Van der Lek RF, de Groot JC, Ruhe HG, Klein HC, Zandstra TE *et al.* Neuroinflammation in bipolar disorder - A [(11)C]-(R)-PK11195 positron emission tomography study. *Brain Behav Immun* 2014; **40:** 219-225.

199.	Loef D, Tendolkar I, Eijndhoven PFP, Hoozemans JJM, Oudega ML, Rozemuller AJM *et al.* Electroconvulsive therapy is associated with increased immunoreactivity of neuroplasticity markers in the hippocampus of depressed patients. *Translational Psychiatry* 2023; **13**(1).

200.	Olsson SK, Samuelsson M, Saetre P, Lindström L, Jönsson EG, Nordin C *et al.* Elevated levels of kynurenic acid in the cerebrospinal fluid of patients with bipolar disorder. *J Psychiatry Neurosci* 2010; **35**(3)**:** 195-199.

201.	Sellgren CM, Gracias J, Jungholm O, Perlis RH, Engberg G, Schwieler L *et al.* Peripheral and central levels of kynurenic acid in bipolar disorder subjects and healthy controls. *Transl Psychiatry* 2019; **9**(1)**:** 37.

202.	Sellgren CM, Kegel ME, Bergen SE, Ekman CJ, Olsson S, Larsson M *et al.* A genome-wide association study of kynurenic acid in cerebrospinal fluid: implications for psychosis and cognitive impairment in bipolar disorder. *Mol Psychiatry* 2016; **21**(10)**:** 1342-1350.

203.	Sneeboer MAM, Snijders G, Berdowski WM, Fernández-Andreu A, van Mierlo HC, Berdenis van Berlekom A *et al.* Microglia in post-mortem brain tissue of patients with bipolar disorder are not immune activated. *Transl Psychiatry* 2019; **9**(1)**:** 153.

204.	Trepci A, Sellgren CM, Pålsson E, Brundin L, Khanlarkhani N, Schwieler L *et al.* Central levels of tryptophan metabolites in subjects with bipolar disorder. *Eur Neuropsychopharmacol* 2021; **43:** 52-62.

205.	Gigase FAJ, Snijders G, Boks MP, de Witte LD. Neurons and glial cells in bipolar disorder: A systematic review of postmortem brain studies of cell number and size. *Neurosci Biobehav Rev* 2019; **103:** 150-162.

206.	Giridharan VV, Sayana P, Pinjari OF, Ahmad N, da Rosa MI, Quevedo J *et al.* Postmortem evidence of brain inflammatory markers in bipolar disorder: a systematic review. *Mol Psychiatry* 2020; **25**(1)**:** 94-113.

207.	Hebbrecht K, Skorobogatov K, Giltay EJ, Coppens V, De Picker L, Morrens M. Tryptophan Catabolites in Bipolar Disorder: A Meta-Analysis. *Front Immunol* 2021; **12:** 667179.

208.	Inam ME, Fernandes BS, Salagre E, Grande I, Vieta E, Quevedo J *et al.* The kynurenine pathway in major depressive disorder, bipolar disorder, and schizophrenia: a systematic review and meta-analysis of cerebrospinal fluid studies. *Braz J Psychiatry* 2023; **45**(4)**:** 343-355.
